# Supplementary figures and images for: Epidermal PAR-6 and PKC-3 are essential for larval development of C. elegans and organize non-centrosomal microtubules
Source: eLife. 2020 Dec 10;9:e62067. doi: 10.7554/eLife.62067 (PMC7755398; doi:10.7554/eLife.62067)

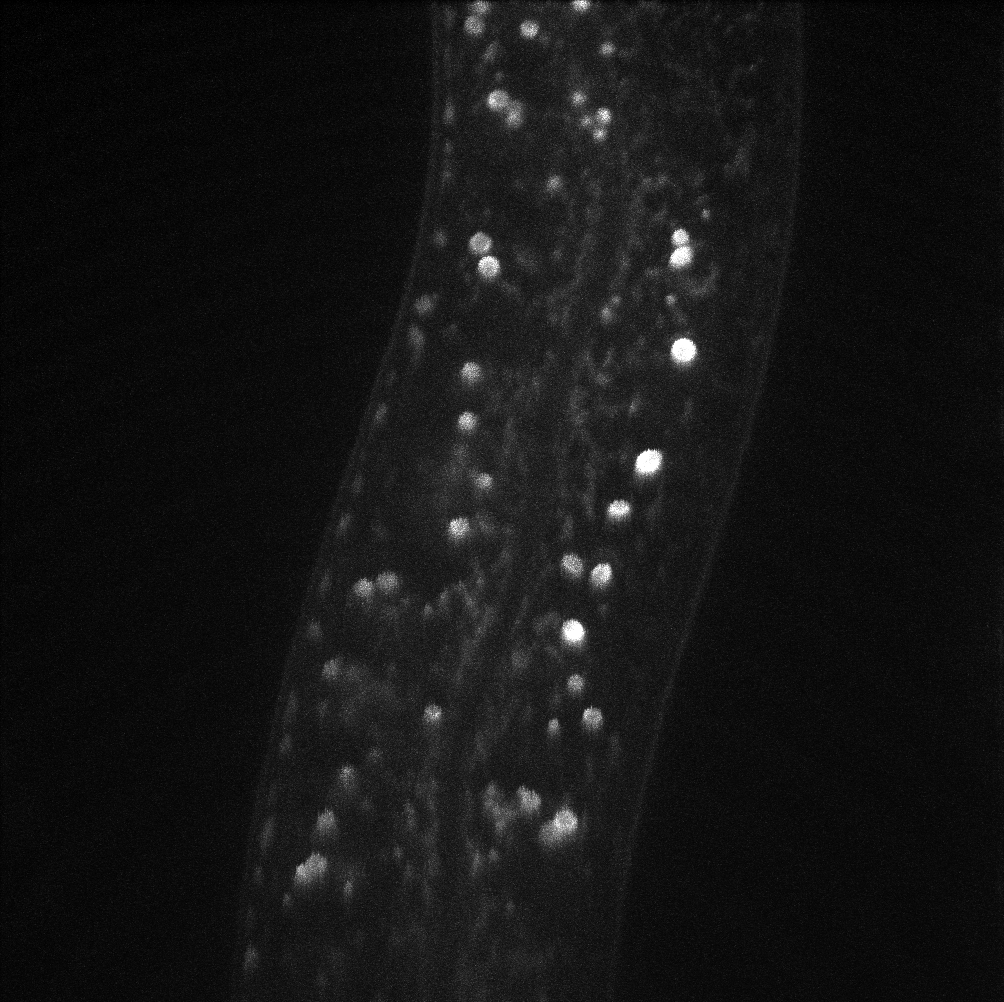

Supplement: Figure 1—source data 1. — This zip archive contains the data graphed in panels C–E, and the microscopy images shown in panel G. The raw graph data are in Microsoft Excel format, and the summary data and actual graph in Graphpad Prism format. The images are in TIFF and Adobe Photoshop format. The TIFF file is the unadjusted grayscale maximum intensity projection image generated in ImageJ from an image stack. The Photoshop file contains the original image with the adjustment layers used to arrive at the final image displayed in the main figure. [file elife-62067-fig1-data1.zip › Fig 1/G/PAR-3/+ auxin/MAX_200701_P3aid_peft3_short_A_11-2-to-7.tif]

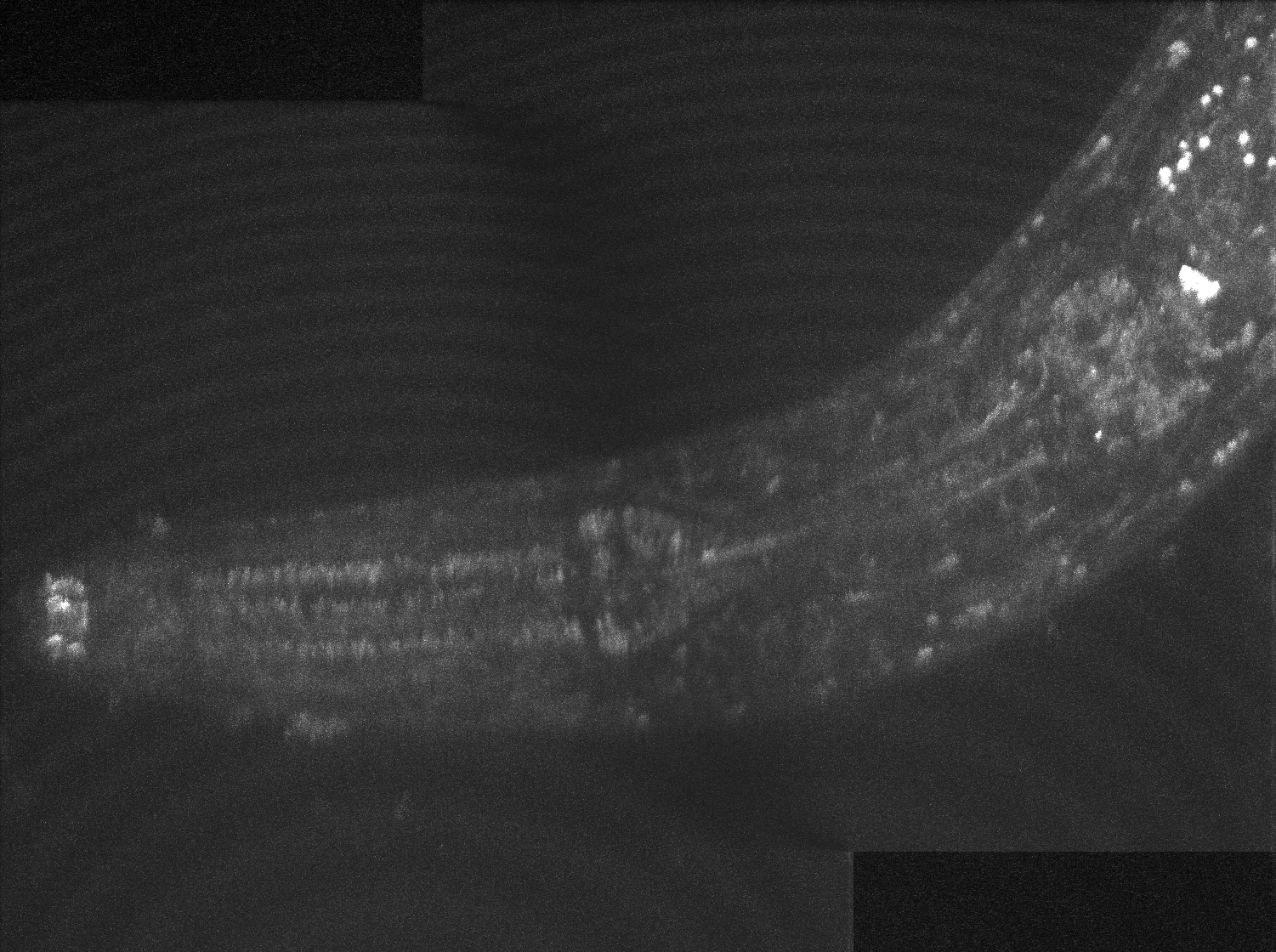

Supplement: Figure 1—source data 1. — This zip archive contains the data graphed in panels C–E, and the microscopy images shown in panel G. The raw graph data are in Microsoft Excel format, and the summary data and actual graph in Graphpad Prism format. The images are in TIFF and Adobe Photoshop format. The TIFF file is the unadjusted grayscale maximum intensity projection image generated in ImageJ from an image stack. The Photoshop file contains the original image with the adjustment layers used to arrive at the final image displayed in the main figure. [file elife-62067-fig1-data1.zip › Fig 1/G/PAR-3/+ auxin/MAX_200701_P3aid_peft3_short_A_5_6-1-to-29.tif]

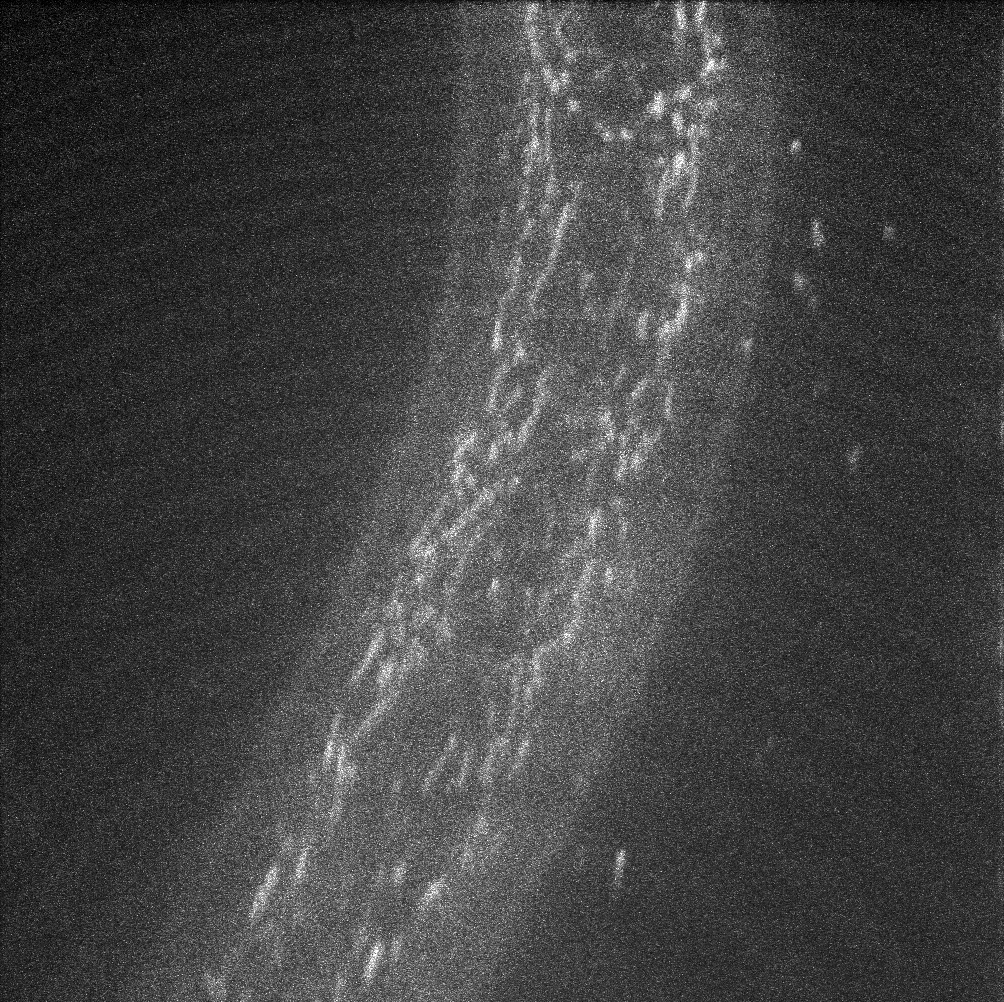

Supplement: Figure 1—source data 1. — This zip archive contains the data graphed in panels C–E, and the microscopy images shown in panel G. The raw graph data are in Microsoft Excel format, and the summary data and actual graph in Graphpad Prism format. The images are in TIFF and Adobe Photoshop format. The TIFF file is the unadjusted grayscale maximum intensity projection image generated in ImageJ from an image stack. The Photoshop file contains the original image with the adjustment layers used to arrive at the final image displayed in the main figure. [file elife-62067-fig1-data1.zip › Fig 1/G/PAR-3/+ auxin/MAX_200701_P3aid_peft3_short_A_8-8-to-10.tif]

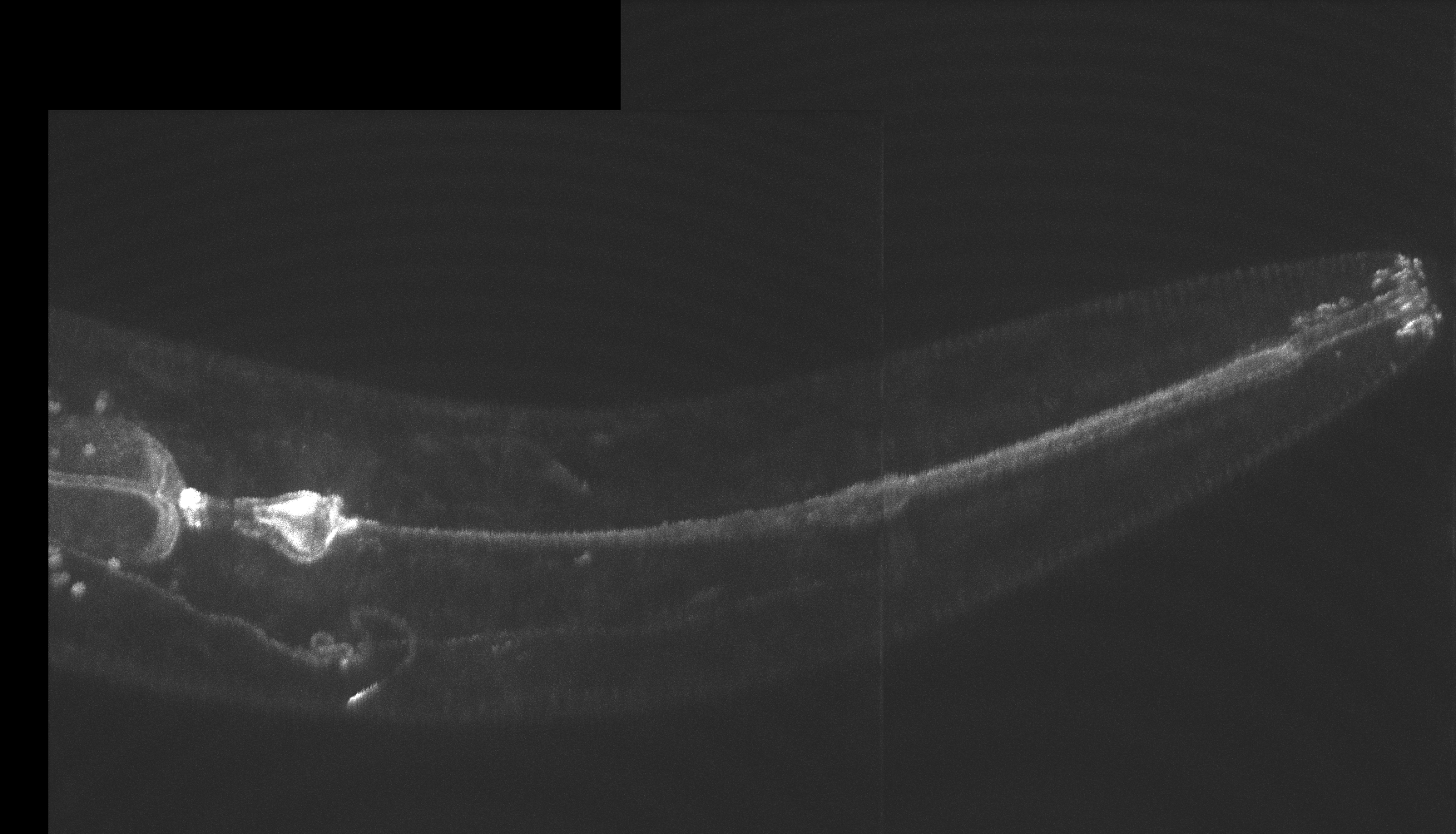

Supplement: Figure 1—source data 1. — This zip archive contains the data graphed in panels C–E, and the microscopy images shown in panel G. The raw graph data are in Microsoft Excel format, and the summary data and actual graph in Graphpad Prism format. The images are in TIFF and Adobe Photoshop format. The TIFF file is the unadjusted grayscale maximum intensity projection image generated in ImageJ from an image stack. The Photoshop file contains the original image with the adjustment layers used to arrive at the final image displayed in the main figure. [file elife-62067-fig1-data1.zip › Fig 1/G/PAR-3/- auxin/MAX_200701_P3aid_peft3_short_C_1_2-1-to-25.tif]

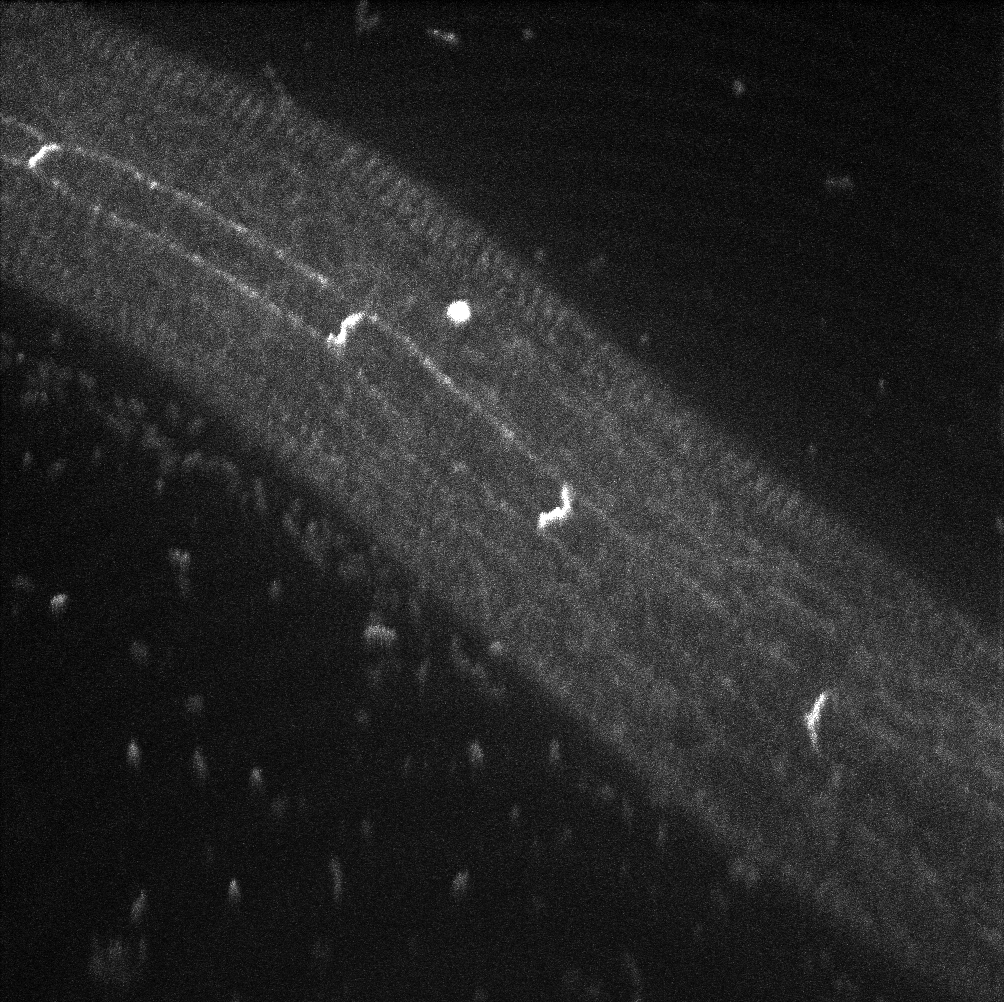

Supplement: Figure 1—source data 1. — This zip archive contains the data graphed in panels C–E, and the microscopy images shown in panel G. The raw graph data are in Microsoft Excel format, and the summary data and actual graph in Graphpad Prism format. The images are in TIFF and Adobe Photoshop format. The TIFF file is the unadjusted grayscale maximum intensity projection image generated in ImageJ from an image stack. The Photoshop file contains the original image with the adjustment layers used to arrive at the final image displayed in the main figure. [file elife-62067-fig1-data1.zip › Fig 1/G/PAR-3/- auxin/MAX_200701_P3aid_peft3_short_C_4-3-to-5.tif]

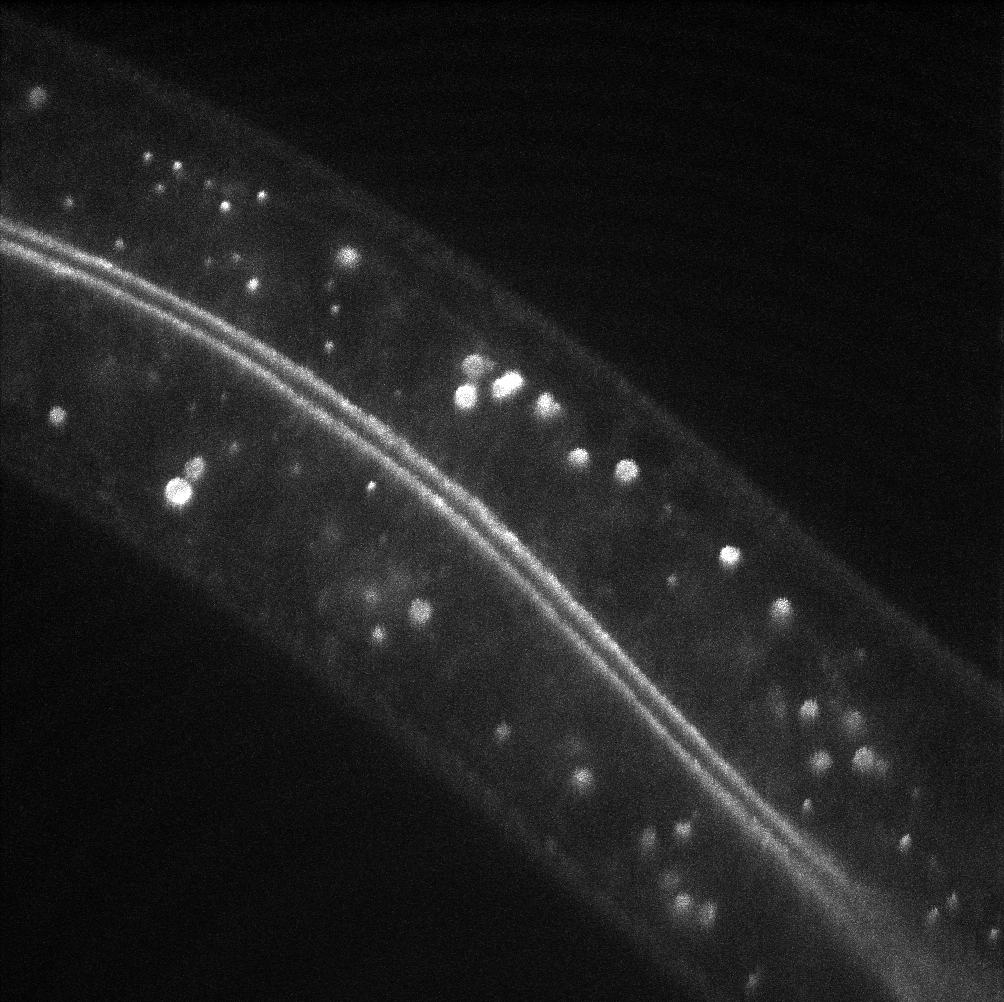

Supplement: Figure 1—source data 1. — This zip archive contains the data graphed in panels C–E, and the microscopy images shown in panel G. The raw graph data are in Microsoft Excel format, and the summary data and actual graph in Graphpad Prism format. The images are in TIFF and Adobe Photoshop format. The TIFF file is the unadjusted grayscale maximum intensity projection image generated in ImageJ from an image stack. The Photoshop file contains the original image with the adjustment layers used to arrive at the final image displayed in the main figure. [file elife-62067-fig1-data1.zip › Fig 1/G/PAR-3/- auxin/MAX_200701_P3aid_peft3_short_C_7-4-to-9.tif]

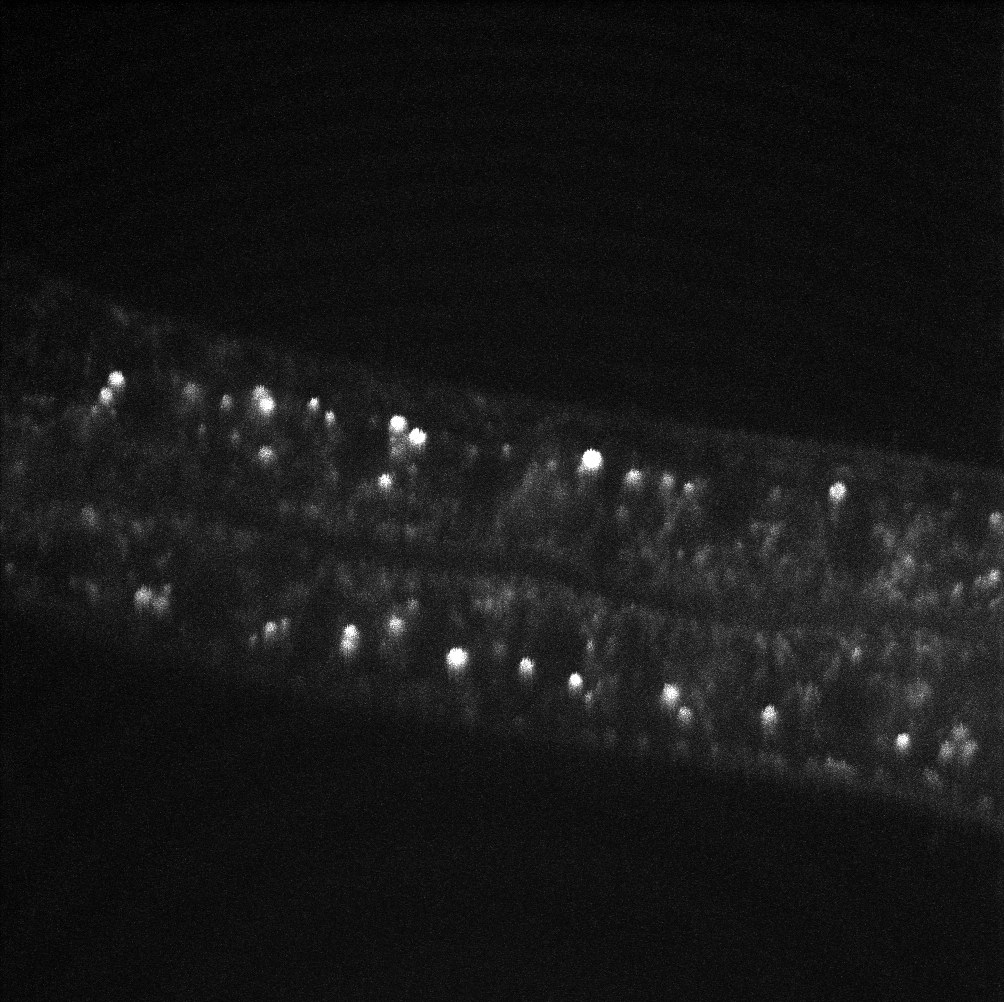

Supplement: Figure 1—source data 1. — This zip archive contains the data graphed in panels C–E, and the microscopy images shown in panel G. The raw graph data are in Microsoft Excel format, and the summary data and actual graph in Graphpad Prism format. The images are in TIFF and Adobe Photoshop format. The TIFF file is the unadjusted grayscale maximum intensity projection image generated in ImageJ from an image stack. The Photoshop file contains the original image with the adjustment layers used to arrive at the final image displayed in the main figure. [file elife-62067-fig1-data1.zip › Fig 1/G/PAR-6/+ auxin/MAX_200319_par6aid_peft3tir1_A_2-1-to-5.tif]

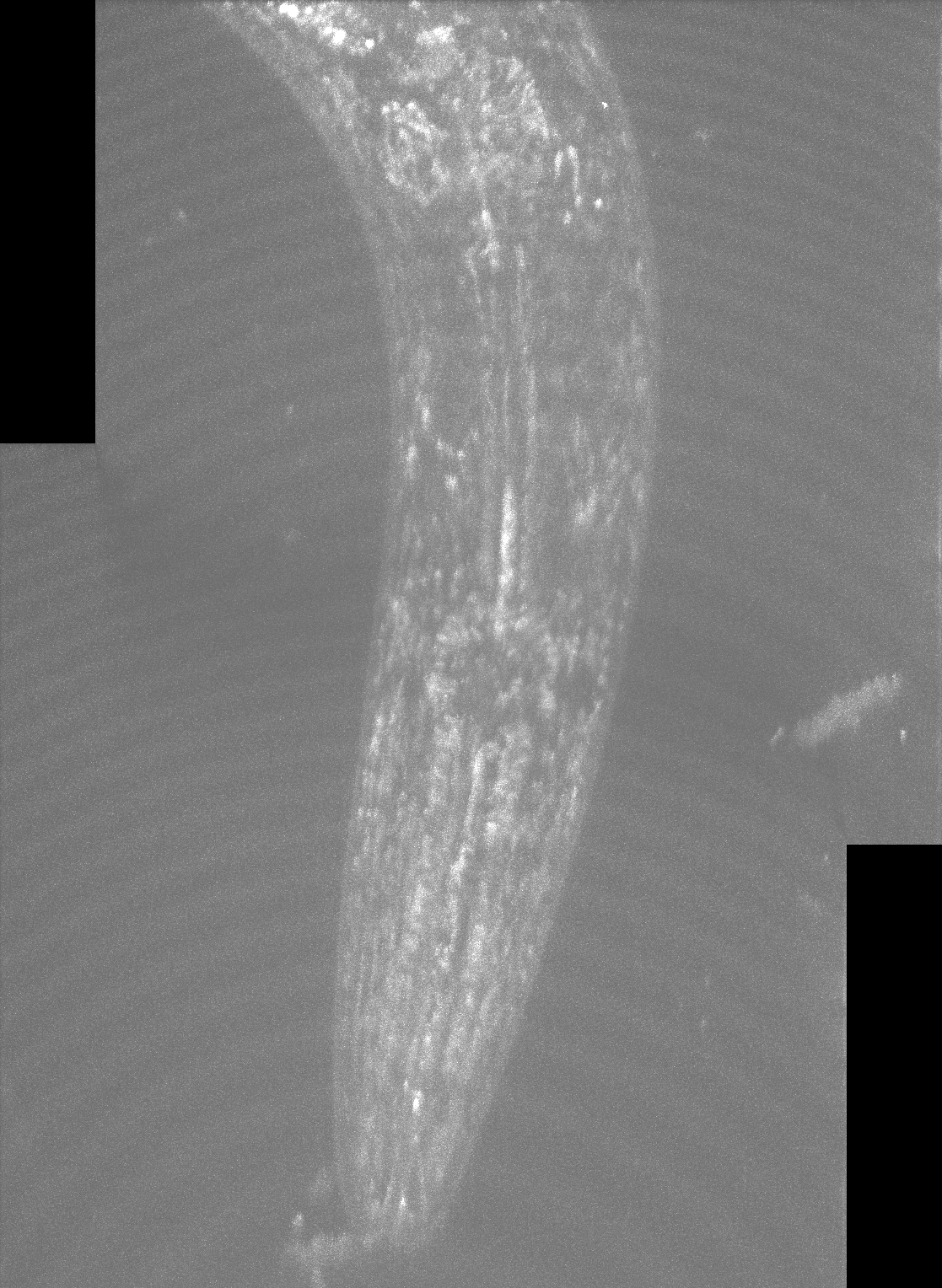

Supplement: Figure 1—source data 1. — This zip archive contains the data graphed in panels C–E, and the microscopy images shown in panel G. The raw graph data are in Microsoft Excel format, and the summary data and actual graph in Graphpad Prism format. The images are in TIFF and Adobe Photoshop format. The TIFF file is the unadjusted grayscale maximum intensity projection image generated in ImageJ from an image stack. The Photoshop file contains the original image with the adjustment layers used to arrive at the final image displayed in the main figure. [file elife-62067-fig1-data1.zip › Fig 1/G/PAR-6/+ auxin/MAX_200319_par6aid_peft3tir1_A_3_4-1-to-19.tif]

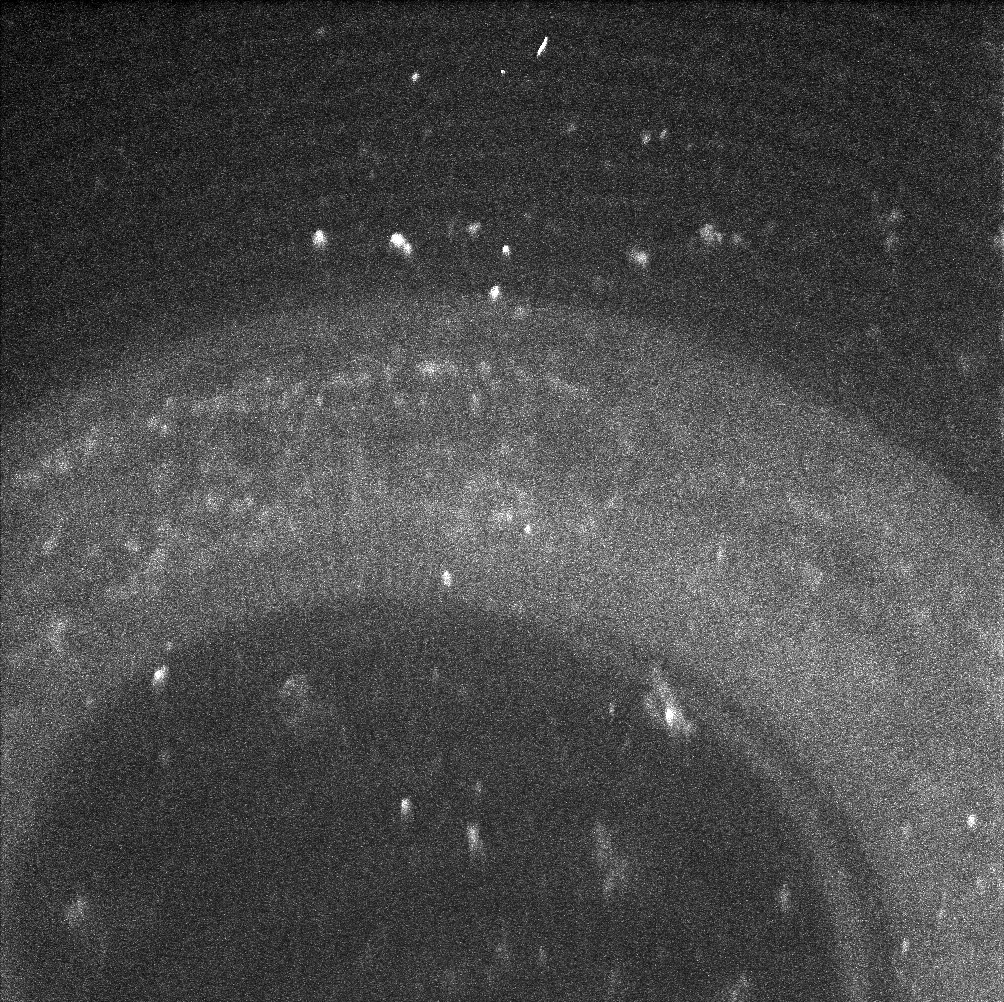

Supplement: Figure 1—source data 1. — This zip archive contains the data graphed in panels C–E, and the microscopy images shown in panel G. The raw graph data are in Microsoft Excel format, and the summary data and actual graph in Graphpad Prism format. The images are in TIFF and Adobe Photoshop format. The TIFF file is the unadjusted grayscale maximum intensity projection image generated in ImageJ from an image stack. The Photoshop file contains the original image with the adjustment layers used to arrive at the final image displayed in the main figure. [file elife-62067-fig1-data1.zip › Fig 1/G/PAR-6/+ auxin/MAX_200319_par6aid_peft3tir1_A_7-7-to-9.tif]

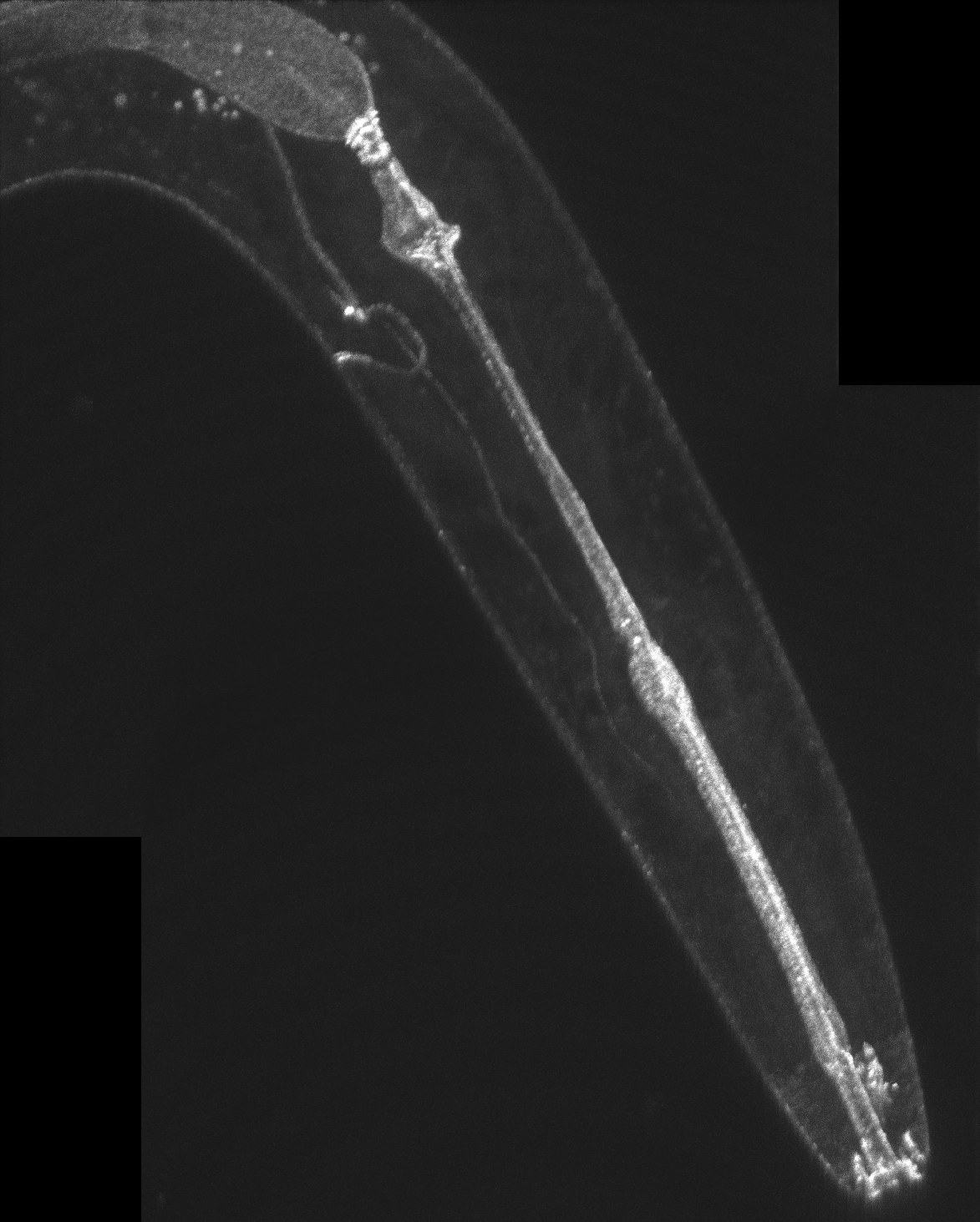

Supplement: Figure 1—source data 1. — This zip archive contains the data graphed in panels C–E, and the microscopy images shown in panel G. The raw graph data are in Microsoft Excel format, and the summary data and actual graph in Graphpad Prism format. The images are in TIFF and Adobe Photoshop format. The TIFF file is the unadjusted grayscale maximum intensity projection image generated in ImageJ from an image stack. The Photoshop file contains the original image with the adjustment layers used to arrive at the final image displayed in the main figure. [file elife-62067-fig1-data1.zip › Fig 1/G/PAR-6/- auxin/MAX_200319_par6aid_peft3tir1_C_3_4-1-to-19.tif]

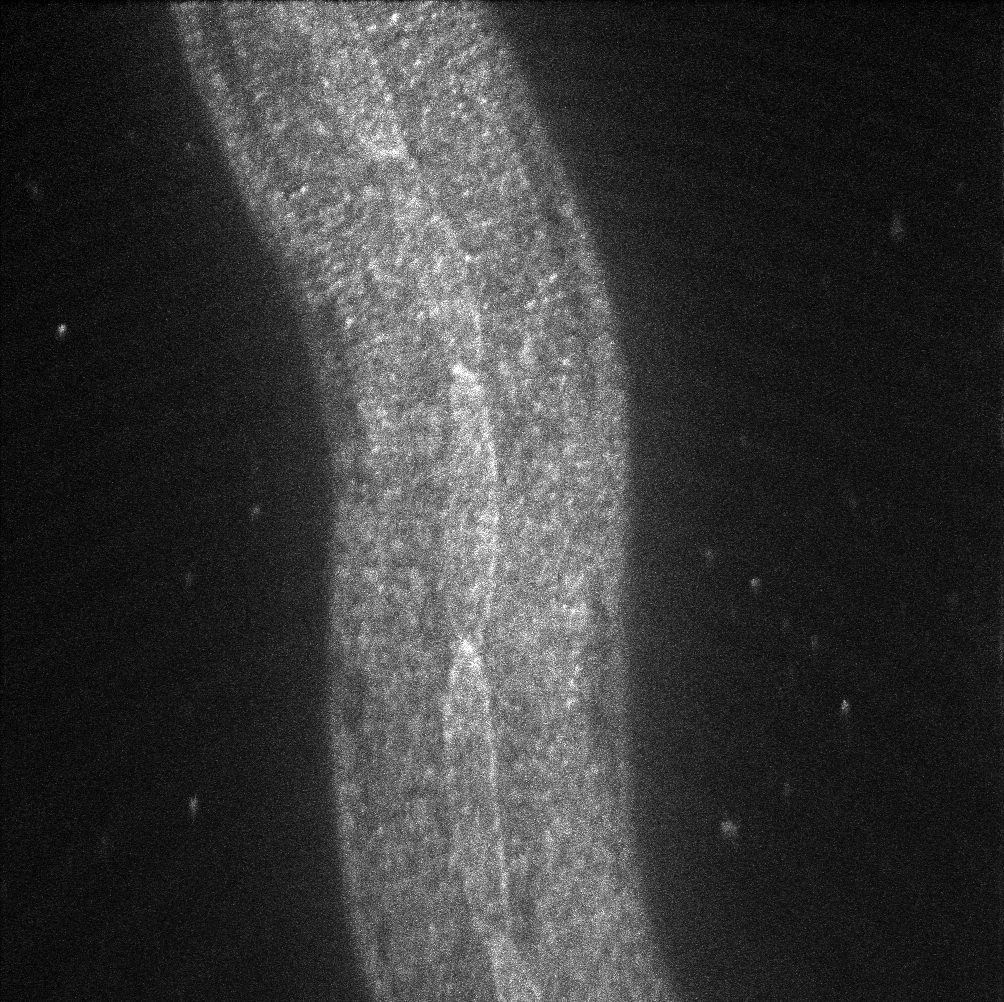

Supplement: Figure 1—source data 1. — This zip archive contains the data graphed in panels C–E, and the microscopy images shown in panel G. The raw graph data are in Microsoft Excel format, and the summary data and actual graph in Graphpad Prism format. The images are in TIFF and Adobe Photoshop format. The TIFF file is the unadjusted grayscale maximum intensity projection image generated in ImageJ from an image stack. The Photoshop file contains the original image with the adjustment layers used to arrive at the final image displayed in the main figure. [file elife-62067-fig1-data1.zip › Fig 1/G/PAR-6/- auxin/MAX_200319_par6aid_peft3tir1_C_5-3-to-5.tif]

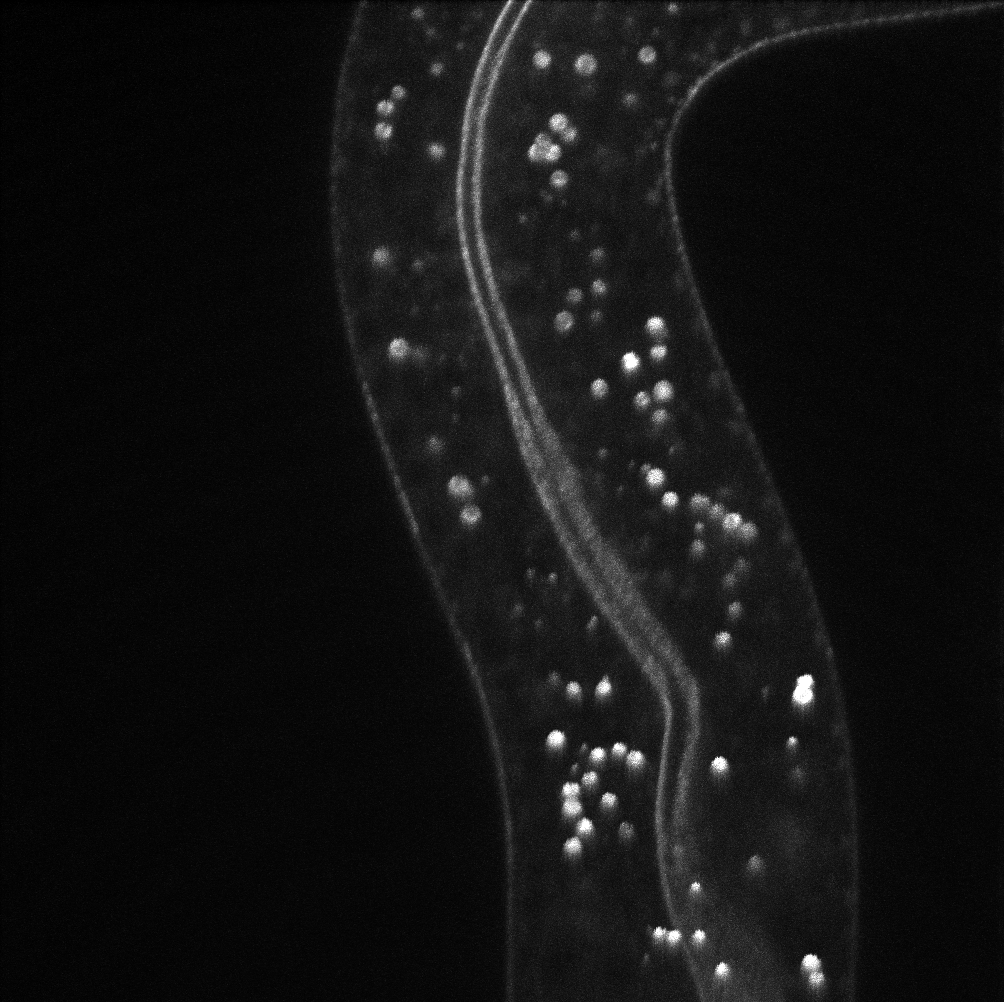

Supplement: Figure 1—source data 1. — This zip archive contains the data graphed in panels C–E, and the microscopy images shown in panel G. The raw graph data are in Microsoft Excel format, and the summary data and actual graph in Graphpad Prism format. The images are in TIFF and Adobe Photoshop format. The TIFF file is the unadjusted grayscale maximum intensity projection image generated in ImageJ from an image stack. The Photoshop file contains the original image with the adjustment layers used to arrive at the final image displayed in the main figure. [file elife-62067-fig1-data1.zip › Fig 1/G/PAR-6/- auxin/MAX_200319_par6aid_peft3tir1_C_7-3-to-7.tif]

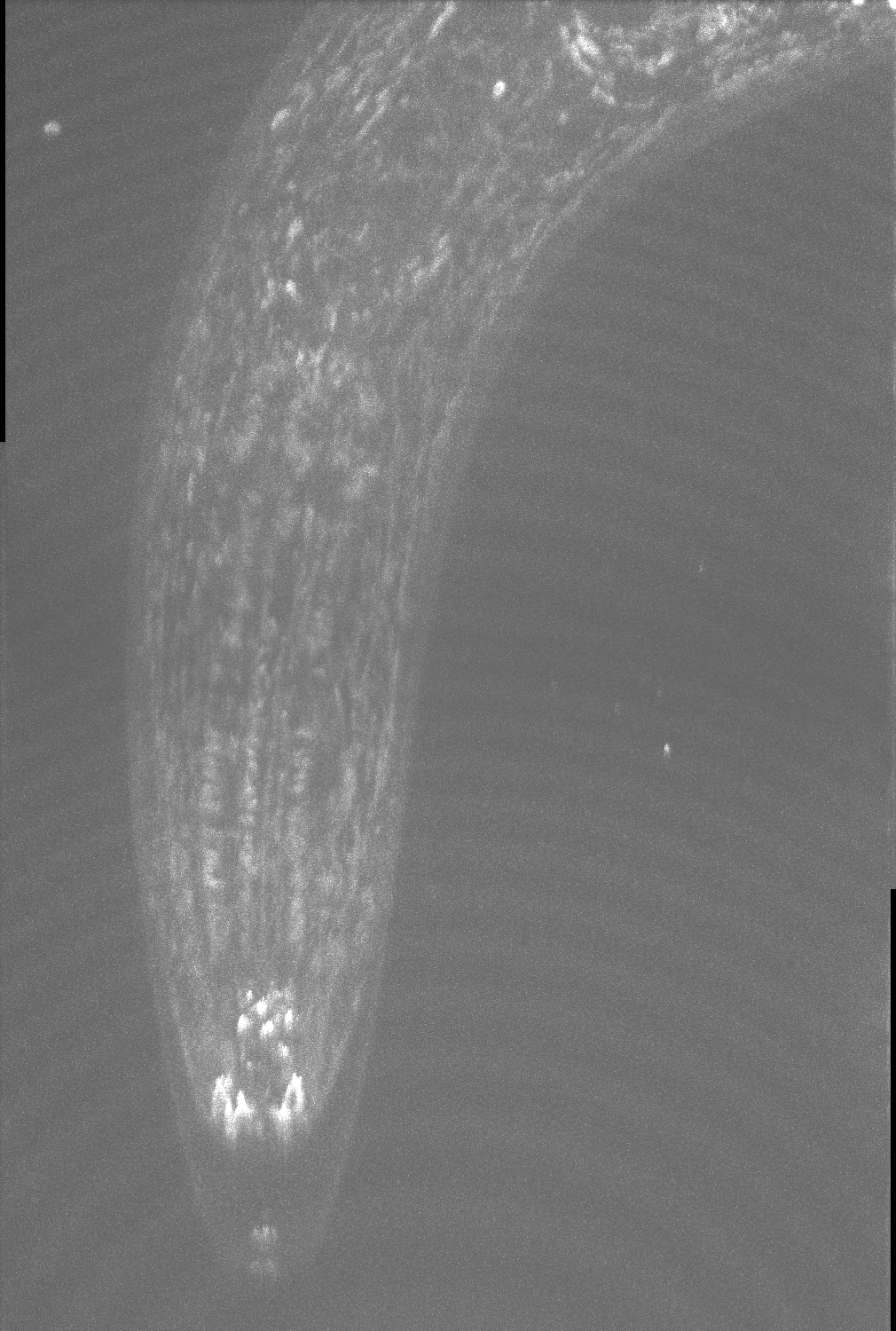

Supplement: Figure 1—source data 1. — This zip archive contains the data graphed in panels C–E, and the microscopy images shown in panel G. The raw graph data are in Microsoft Excel format, and the summary data and actual graph in Graphpad Prism format. The images are in TIFF and Adobe Photoshop format. The TIFF file is the unadjusted grayscale maximum intensity projection image generated in ImageJ from an image stack. The Photoshop file contains the original image with the adjustment layers used to arrive at the final image displayed in the main figure. [file elife-62067-fig1-data1.zip › Fig 1/G/PKC-3/+ auxin/MAX_200319_pkc3aid_peft3tir1_13_14-1-to-19.tif]

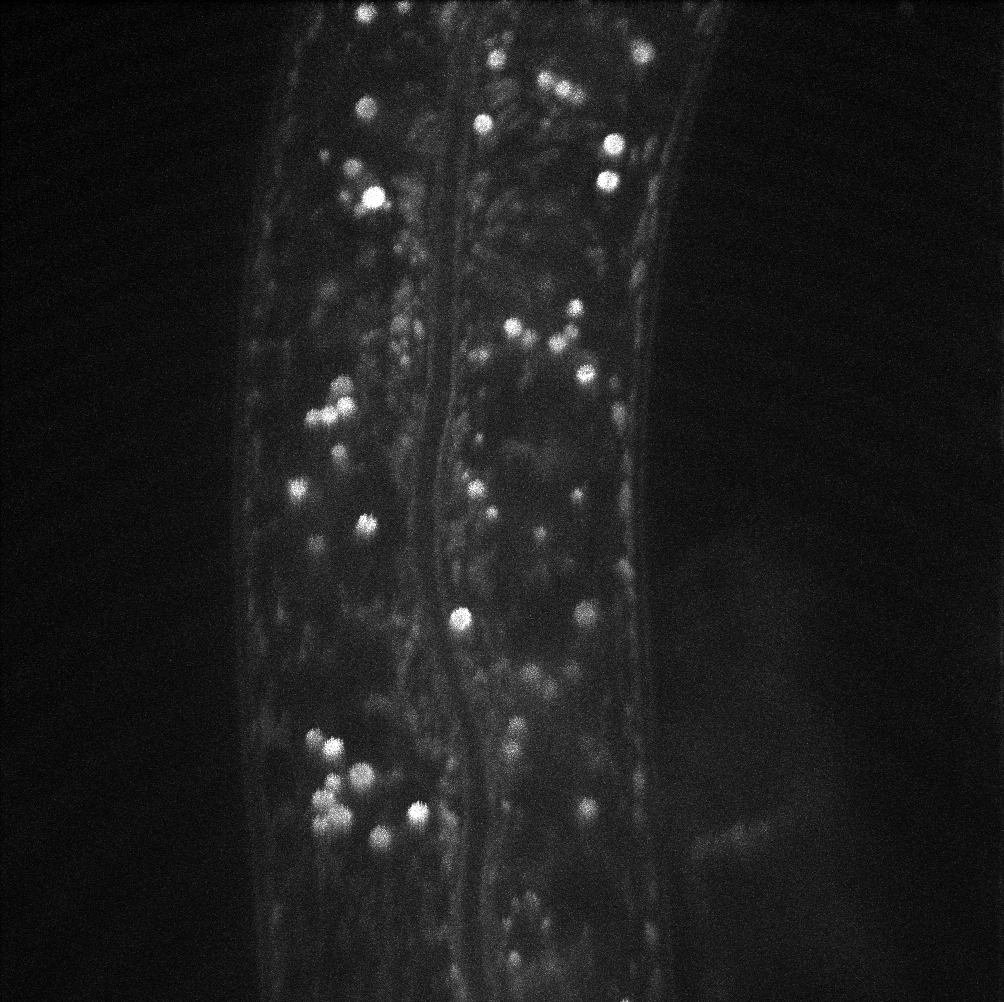

Supplement: Figure 1—source data 1. — This zip archive contains the data graphed in panels C–E, and the microscopy images shown in panel G. The raw graph data are in Microsoft Excel format, and the summary data and actual graph in Graphpad Prism format. The images are in TIFF and Adobe Photoshop format. The TIFF file is the unadjusted grayscale maximum intensity projection image generated in ImageJ from an image stack. The Photoshop file contains the original image with the adjustment layers used to arrive at the final image displayed in the main figure. [file elife-62067-fig1-data1.zip › Fig 1/G/PKC-3/+ auxin/MAX_200319_pkc3aid_peft3tir1_A_19-4-to-12.tif]

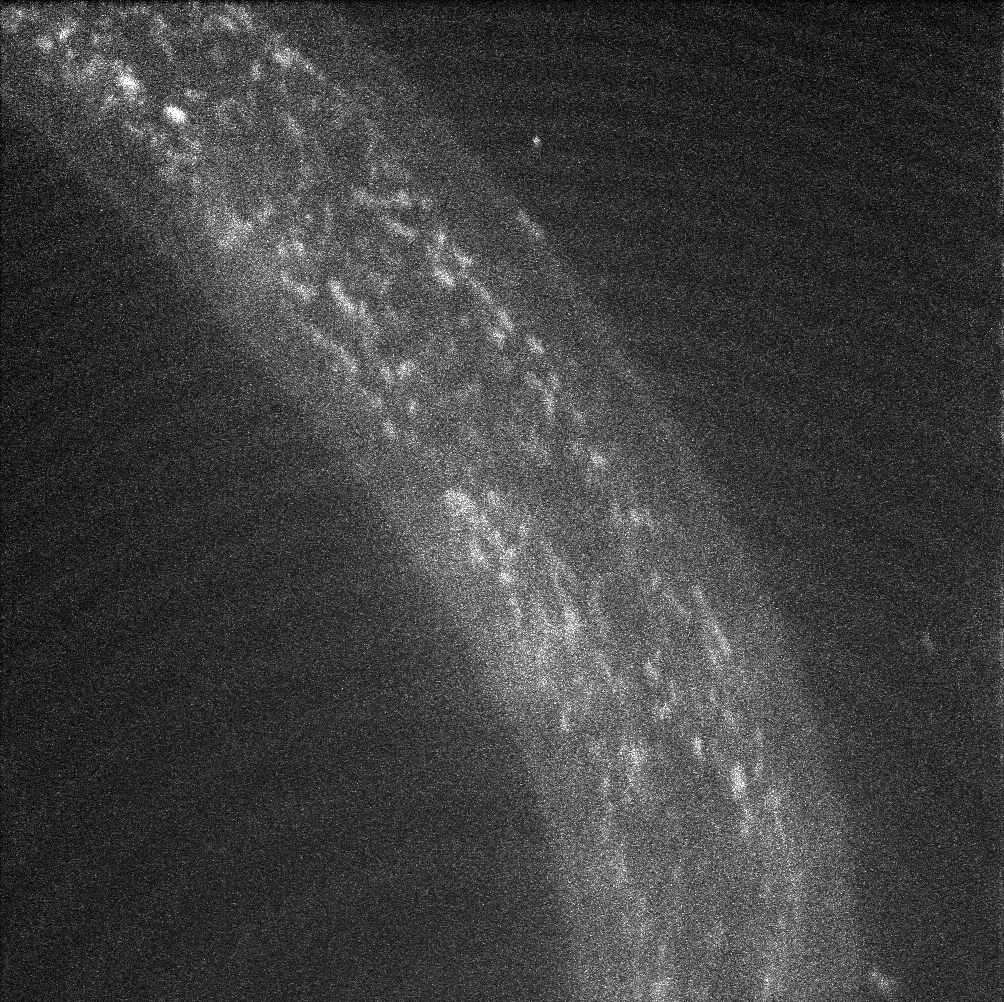

Supplement: Figure 1—source data 1. — This zip archive contains the data graphed in panels C–E, and the microscopy images shown in panel G. The raw graph data are in Microsoft Excel format, and the summary data and actual graph in Graphpad Prism format. The images are in TIFF and Adobe Photoshop format. The TIFF file is the unadjusted grayscale maximum intensity projection image generated in ImageJ from an image stack. The Photoshop file contains the original image with the adjustment layers used to arrive at the final image displayed in the main figure. [file elife-62067-fig1-data1.zip › Fig 1/G/PKC-3/+ auxin/MAX_200319_pkc3aid_peft3tir1_A_24-7-to-9.tif]

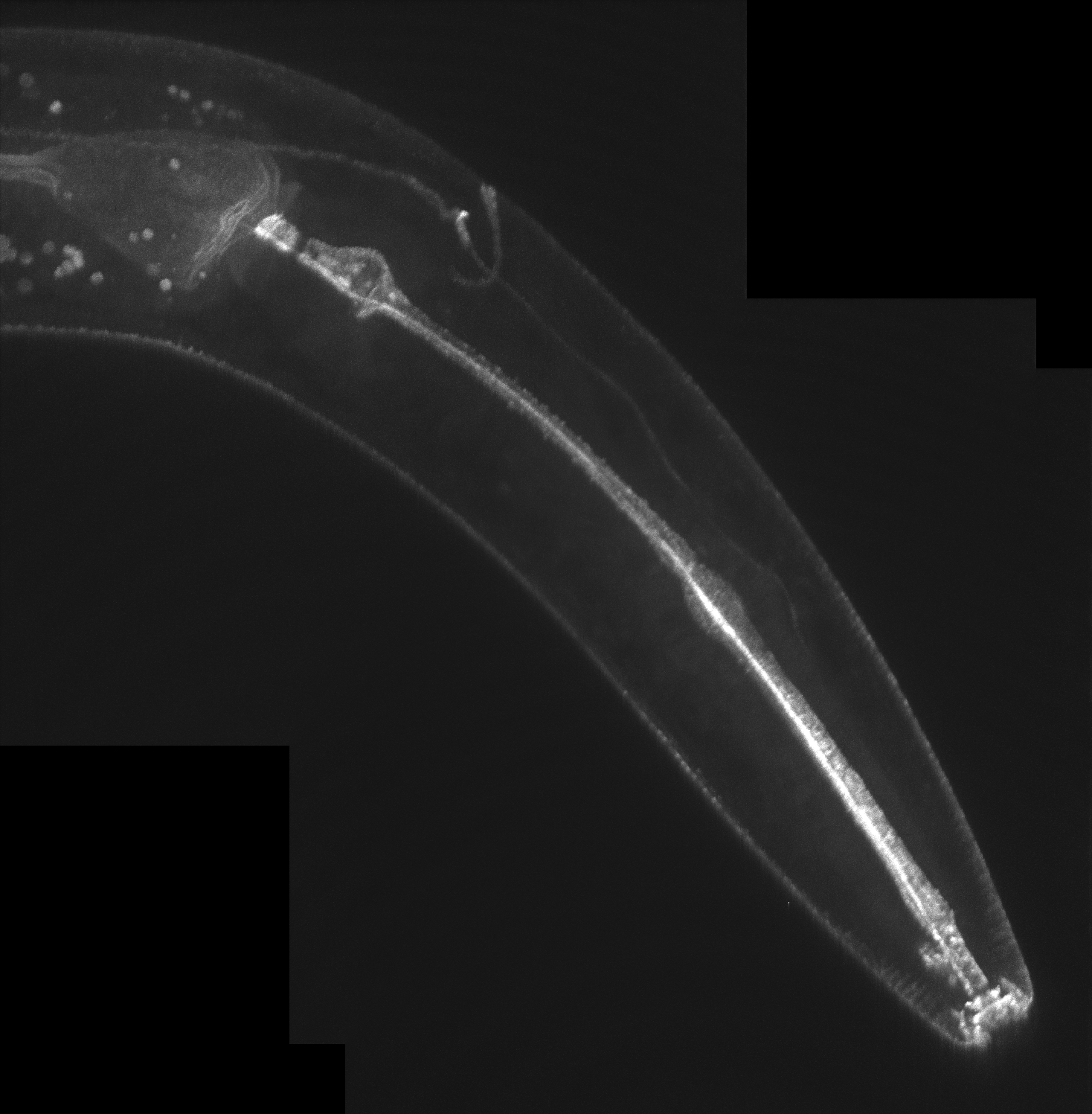

Supplement: Figure 1—source data 1. — This zip archive contains the data graphed in panels C–E, and the microscopy images shown in panel G. The raw graph data are in Microsoft Excel format, and the summary data and actual graph in Graphpad Prism format. The images are in TIFF and Adobe Photoshop format. The TIFF file is the unadjusted grayscale maximum intensity projection image generated in ImageJ from an image stack. The Photoshop file contains the original image with the adjustment layers used to arrive at the final image displayed in the main figure. [file elife-62067-fig1-data1.zip › Fig 1/G/PKC-3/- auxin/200319_pkc3aid_peft3tir1_C_5-7-allstacks.tif]

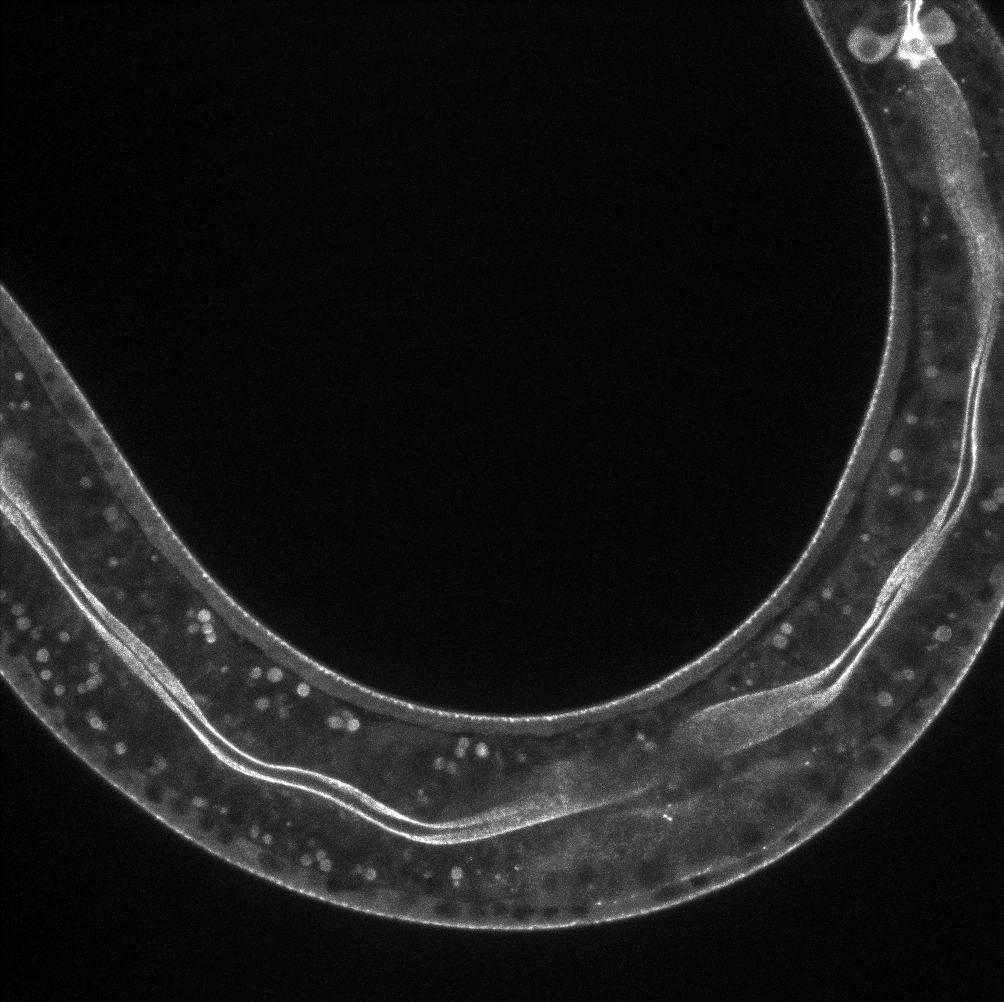

Supplement: Figure 1—source data 1. — This zip archive contains the data graphed in panels C–E, and the microscopy images shown in panel G. The raw graph data are in Microsoft Excel format, and the summary data and actual graph in Graphpad Prism format. The images are in TIFF and Adobe Photoshop format. The TIFF file is the unadjusted grayscale maximum intensity projection image generated in ImageJ from an image stack. The Photoshop file contains the original image with the adjustment layers used to arrive at the final image displayed in the main figure. [file elife-62067-fig1-data1.zip › Fig 1/G/PKC-3/- auxin/MAX_190613_3.18.2.2_Control_9_w1SpinningDisc - Green-22-to-30.tif]

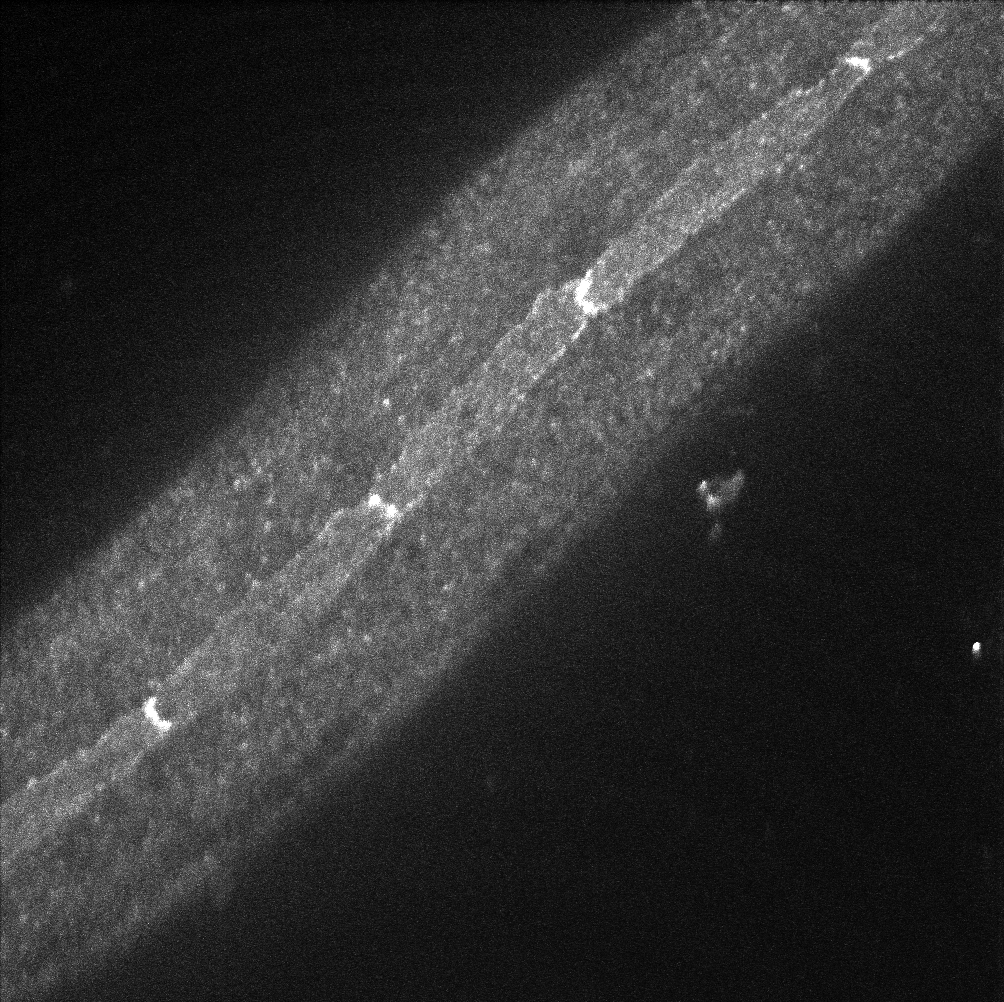

Supplement: Figure 1—source data 1. — This zip archive contains the data graphed in panels C–E, and the microscopy images shown in panel G. The raw graph data are in Microsoft Excel format, and the summary data and actual graph in Graphpad Prism format. The images are in TIFF and Adobe Photoshop format. The TIFF file is the unadjusted grayscale maximum intensity projection image generated in ImageJ from an image stack. The Photoshop file contains the original image with the adjustment layers used to arrive at the final image displayed in the main figure. [file elife-62067-fig1-data1.zip › Fig 1/G/PKC-3/- auxin/MAX_200319_pkc3aid_peft3tir1_C_9-4-to-6.tif]

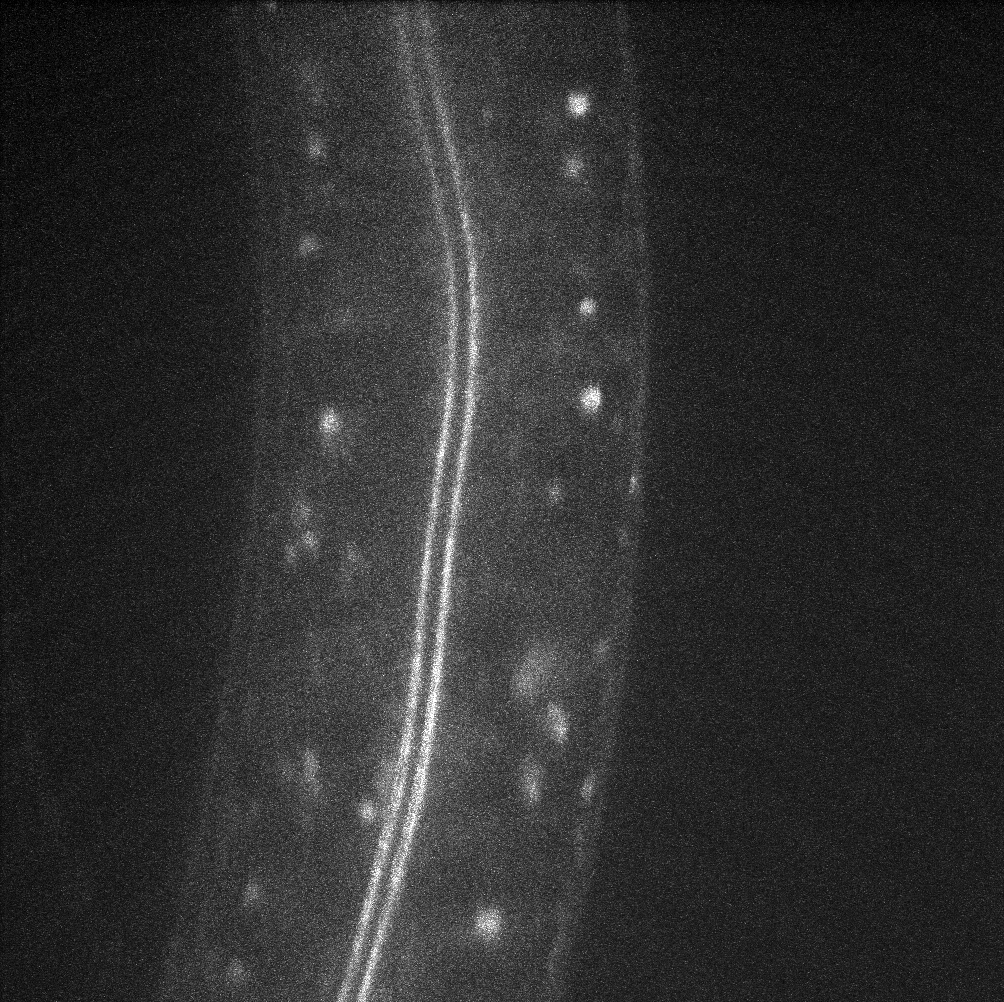

Supplement: Figure 1—figure supplement 1—source data 1. — This zip archive contains the microscopy images shown in panels D and E. The images are in TIFF and Adobe Photoshop format. The TIFF file is the unadjusted grayscale maximum intensity projection image generated in ImageJ from an image stack. The Photoshop file contains the original image with the adjustment layers used to arrive at the final image displayed in the main figure. [file elife-62067-fig1-figsupp1-data1.zip › Fig 1S1/D/MAX_200903_P3aid_long_NGM_5-11-to-13.tif]

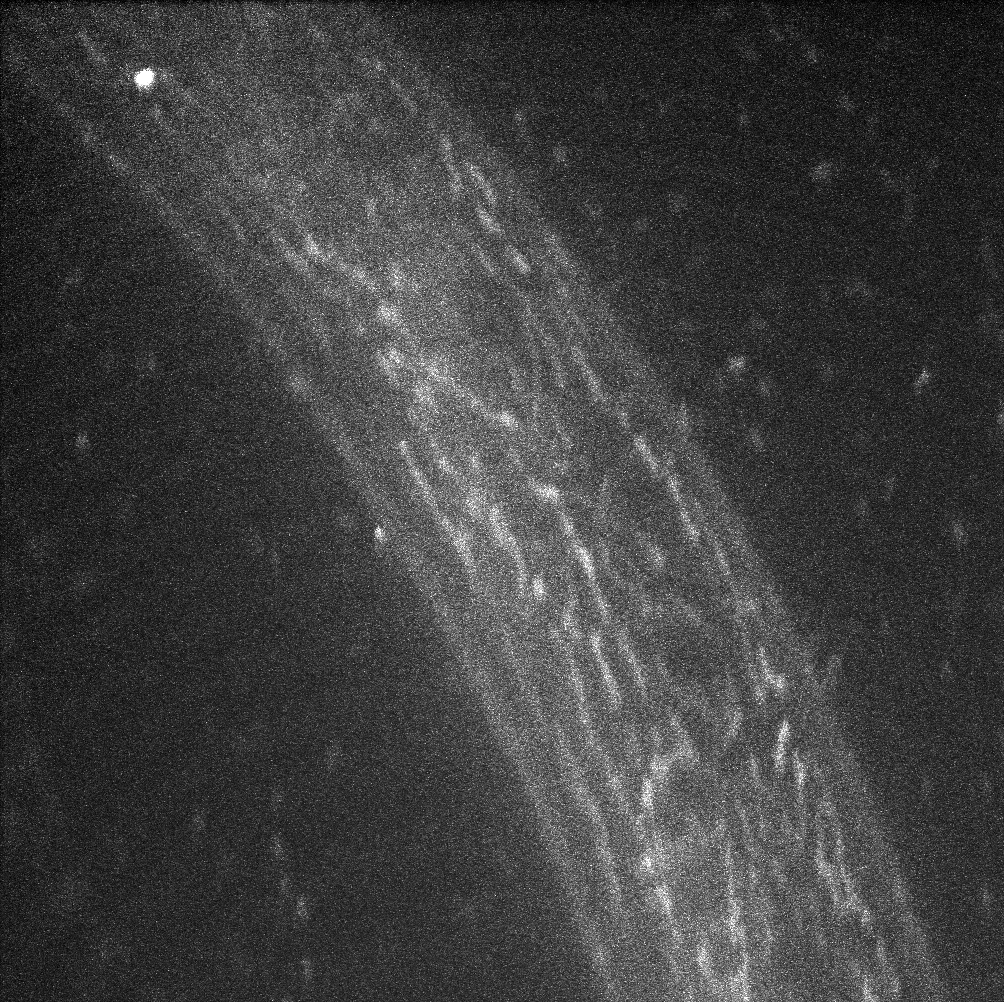

Supplement: Figure 1—figure supplement 1—source data 1. — This zip archive contains the microscopy images shown in panels D and E. The images are in TIFF and Adobe Photoshop format. The TIFF file is the unadjusted grayscale maximum intensity projection image generated in ImageJ from an image stack. The Photoshop file contains the original image with the adjustment layers used to arrive at the final image displayed in the main figure. [file elife-62067-fig1-figsupp1-data1.zip › Fig 1S1/D/MAX_200903_P3aid_long_NGM_6-11-to-13.tif]

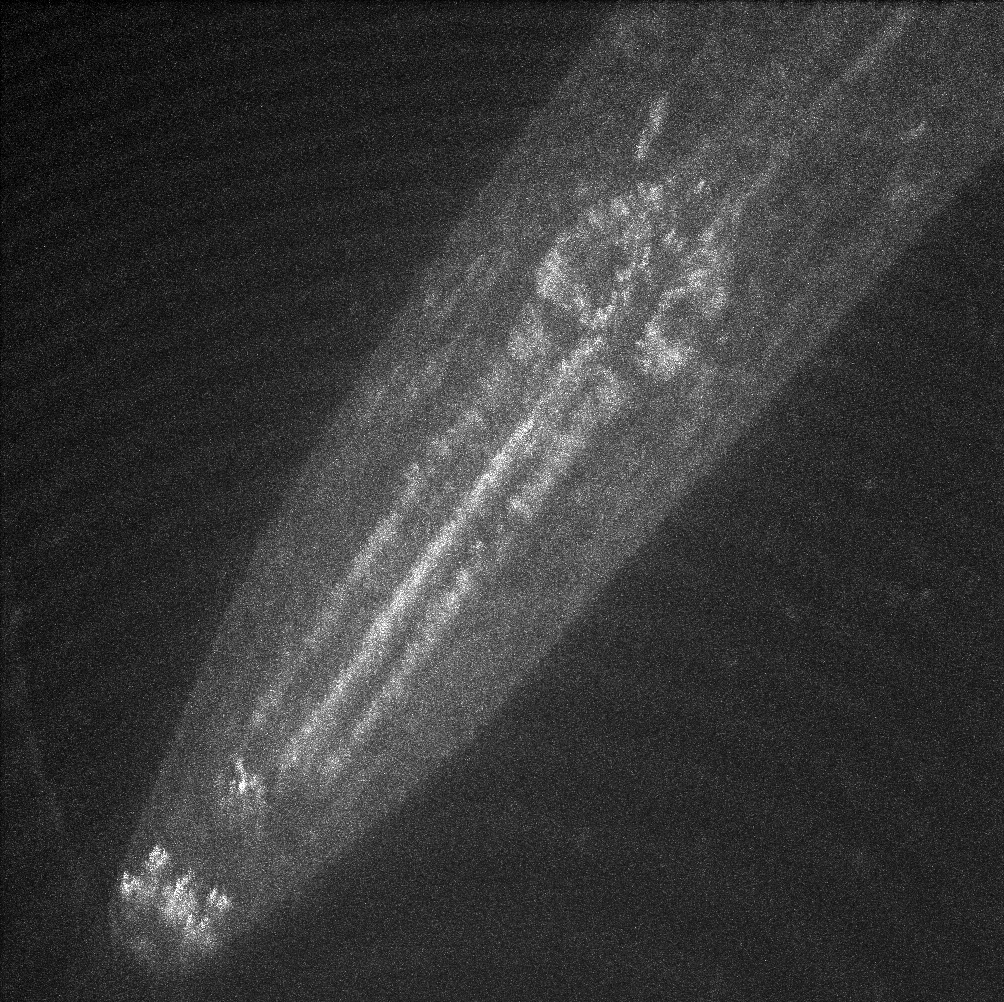

Supplement: Figure 1—figure supplement 1—source data 1. — This zip archive contains the microscopy images shown in panels D and E. The images are in TIFF and Adobe Photoshop format. The TIFF file is the unadjusted grayscale maximum intensity projection image generated in ImageJ from an image stack. The Photoshop file contains the original image with the adjustment layers used to arrive at the final image displayed in the main figure. [file elife-62067-fig1-figsupp1-data1.zip › Fig 1S1/D/MAX_200903_P3aid_single_NGM_7.tif]

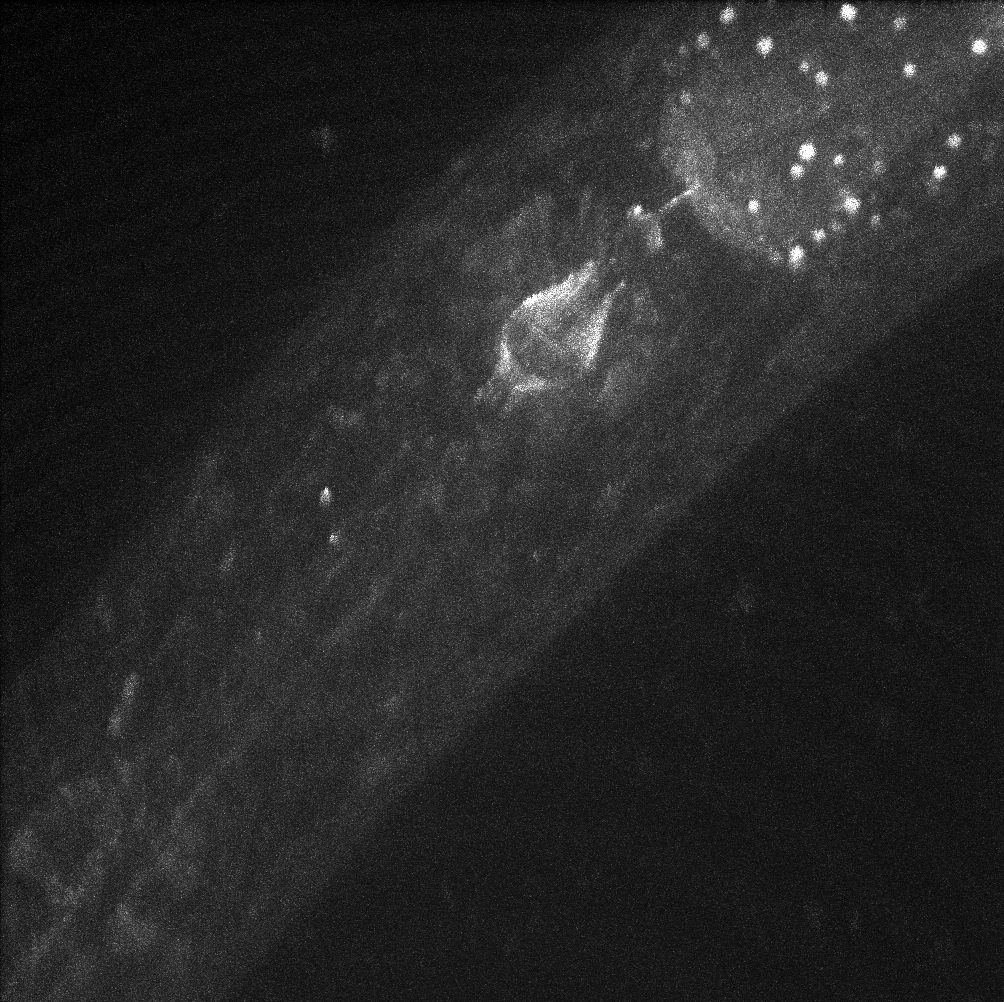

Supplement: Figure 1—figure supplement 1—source data 1. — This zip archive contains the microscopy images shown in panels D and E. The images are in TIFF and Adobe Photoshop format. The TIFF file is the unadjusted grayscale maximum intensity projection image generated in ImageJ from an image stack. The Photoshop file contains the original image with the adjustment layers used to arrive at the final image displayed in the main figure. [file elife-62067-fig1-figsupp1-data1.zip › Fig 1S1/D/MAX_200903_P3aid_single_NGM_8.tif]

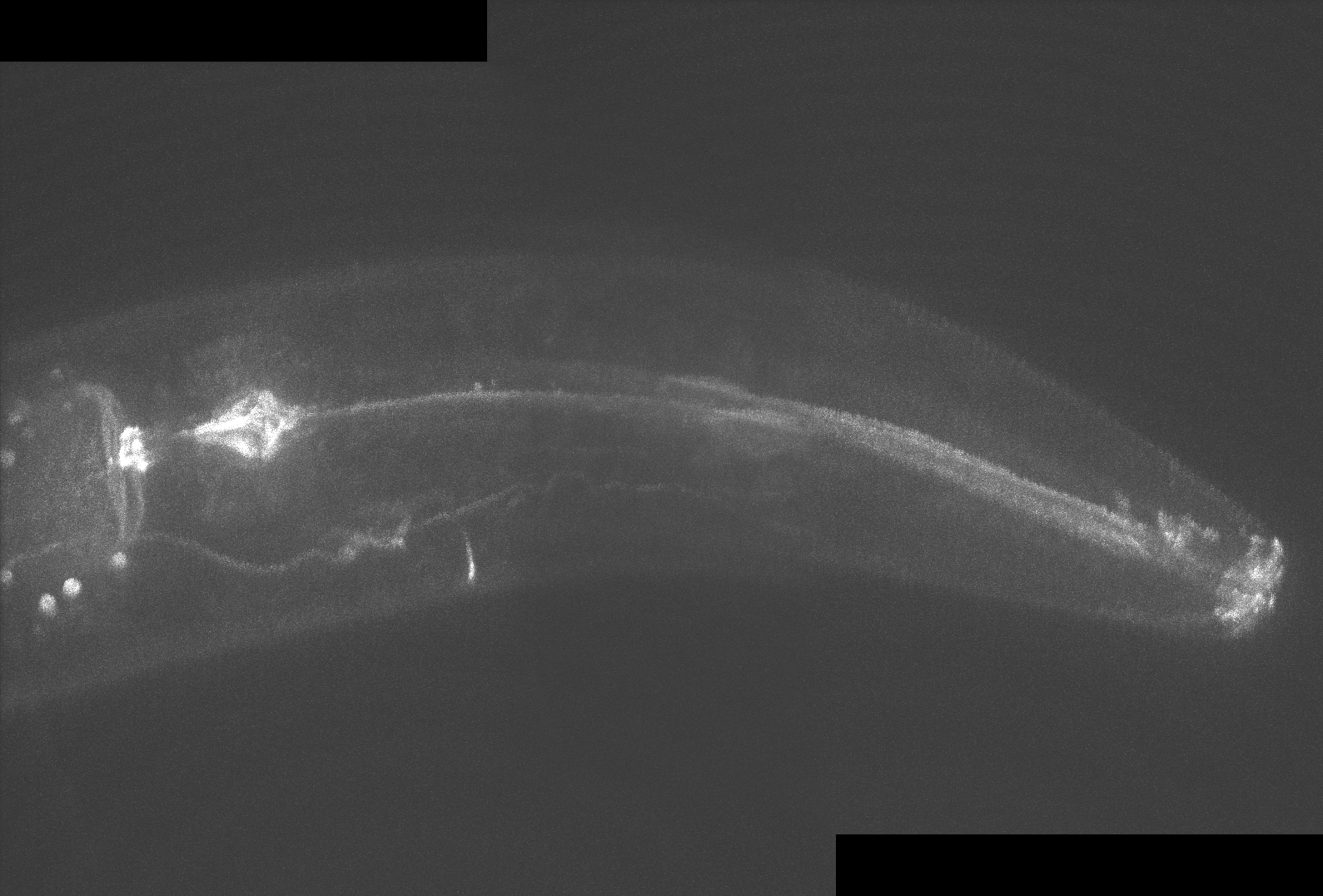

Supplement: Figure 1—figure supplement 1—source data 1. — This zip archive contains the microscopy images shown in panels D and E. The images are in TIFF and Adobe Photoshop format. The TIFF file is the unadjusted grayscale maximum intensity projection image generated in ImageJ from an image stack. The Photoshop file contains the original image with the adjustment layers used to arrive at the final image displayed in the main figure. [file elife-62067-fig1-figsupp1-data1.zip › Fig 1S1/E/MAX_200903_P3aid_double_NGM_1-2.tif]

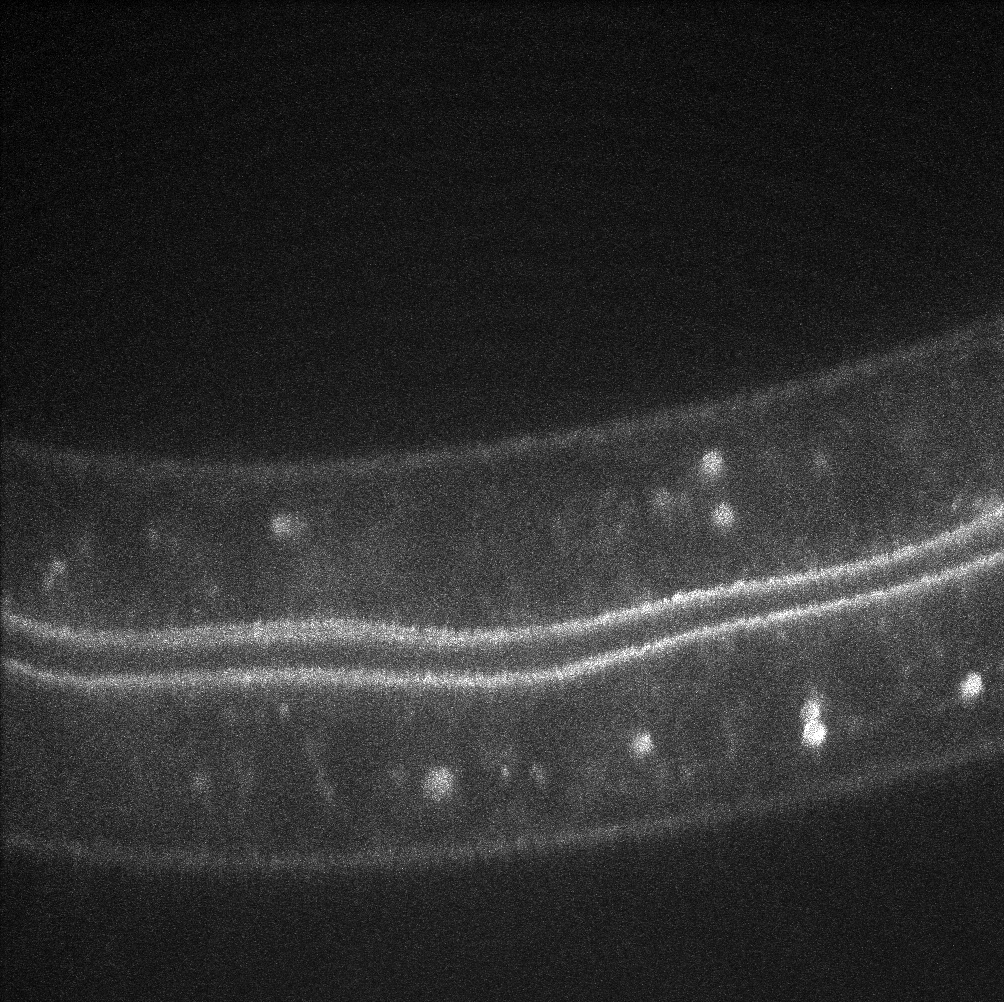

Supplement: Figure 1—figure supplement 1—source data 1. — This zip archive contains the microscopy images shown in panels D and E. The images are in TIFF and Adobe Photoshop format. The TIFF file is the unadjusted grayscale maximum intensity projection image generated in ImageJ from an image stack. The Photoshop file contains the original image with the adjustment layers used to arrive at the final image displayed in the main figure. [file elife-62067-fig1-figsupp1-data1.zip › Fig 1S1/E/MAX_200903_P3aid_double_NGM_3-4-to-6.tif]

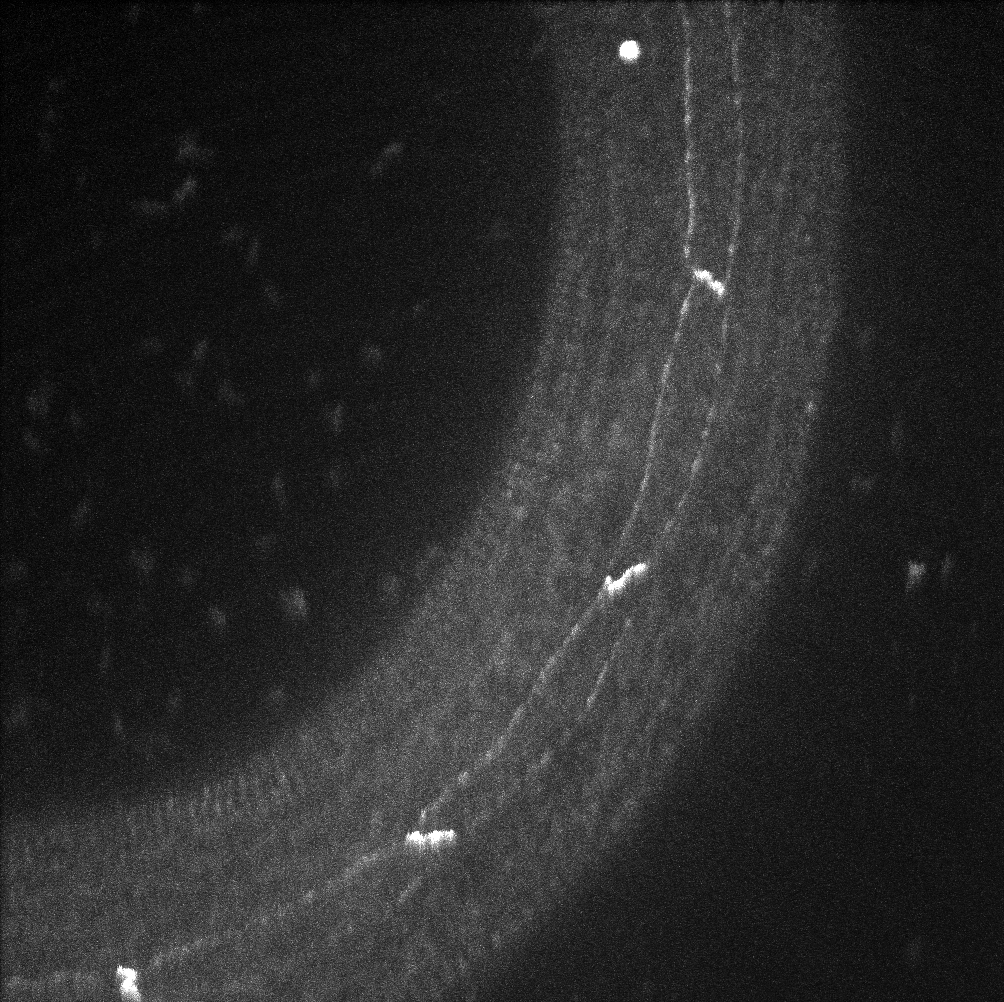

Supplement: Figure 1—figure supplement 1—source data 1. — This zip archive contains the microscopy images shown in panels D and E. The images are in TIFF and Adobe Photoshop format. The TIFF file is the unadjusted grayscale maximum intensity projection image generated in ImageJ from an image stack. The Photoshop file contains the original image with the adjustment layers used to arrive at the final image displayed in the main figure. [file elife-62067-fig1-figsupp1-data1.zip › Fig 1S1/E/MAX_200903_P3aid_double_NGM_7-1-to-5.tif]

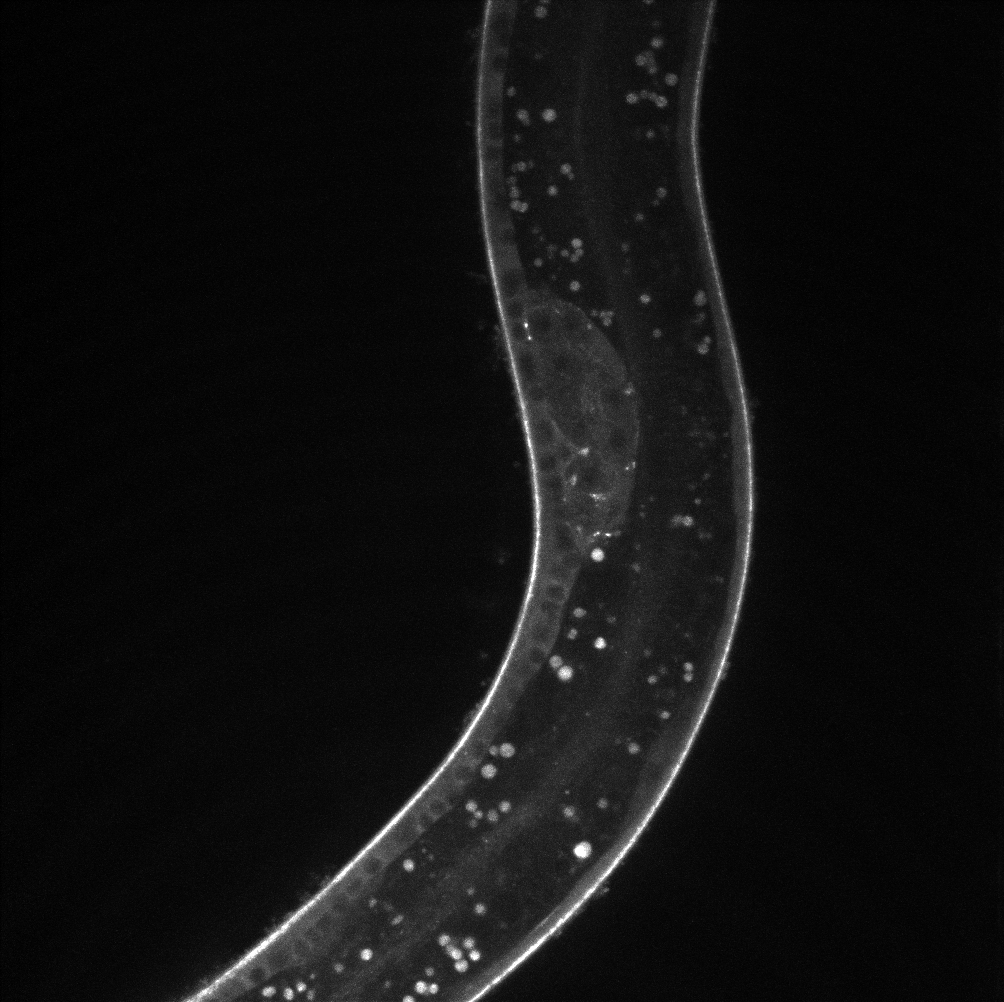

Supplement: Figure 2—source data 1. — This zip archive contains the microscopy images shown in panel A and B, and the data graphed in panels C–J. The images are in TIFF and Adobe Photoshop format. The TIFF file is the unadjusted grayscale maximum intensity projection image generated in ImageJ from an image stack. The Photoshop file contains the original image with the adjustment layers used to arrive at the final image displayed in the main figure. The raw graph data are in Microsoft Excel format, and the summary data and actual graph in Graphpad Prism format. [file elife-62067-fig2-data1.zip › Fig 2/A/MAX_190116_P6deg_eGFP_pelt2_TIR_Auxin_l30-17-to-23.tif]

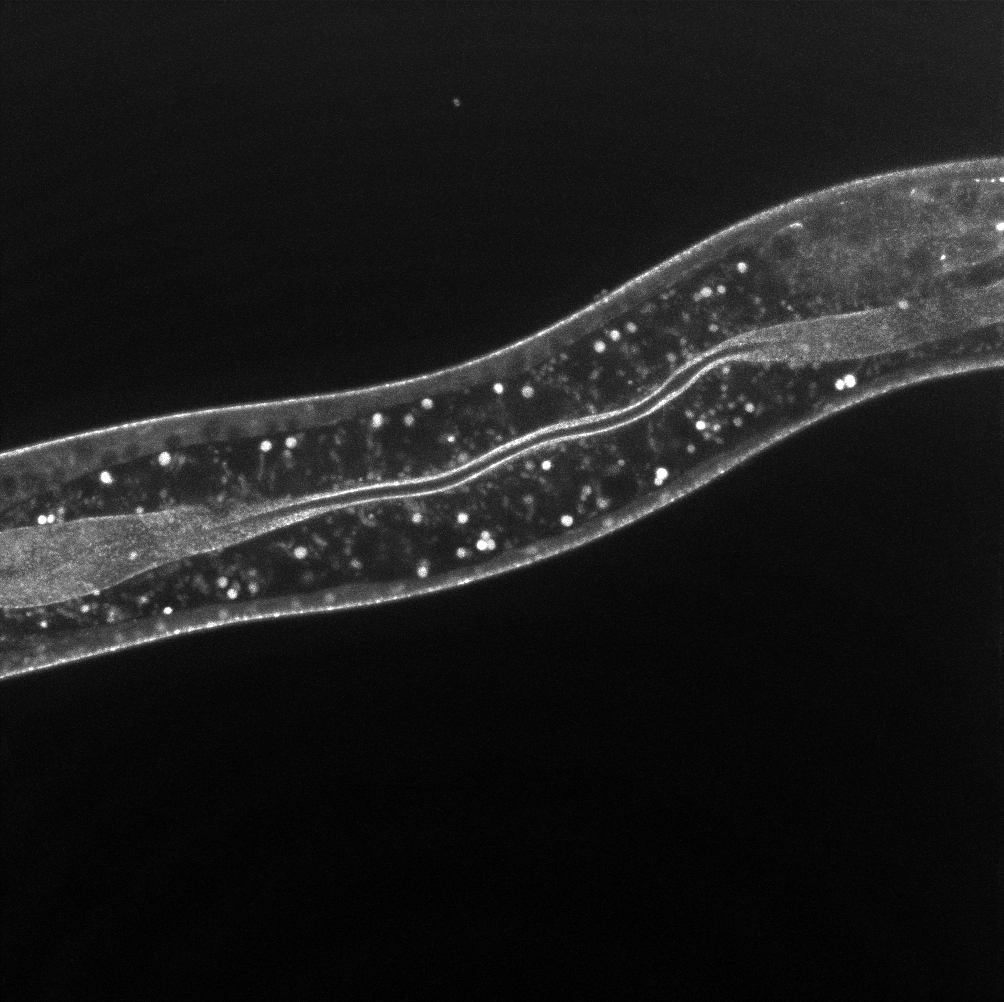

Supplement: Figure 2—source data 1. — This zip archive contains the microscopy images shown in panel A and B, and the data graphed in panels C–J. The images are in TIFF and Adobe Photoshop format. The TIFF file is the unadjusted grayscale maximum intensity projection image generated in ImageJ from an image stack. The Photoshop file contains the original image with the adjustment layers used to arrive at the final image displayed in the main figure. The raw graph data are in Microsoft Excel format, and the summary data and actual graph in Graphpad Prism format. [file elife-62067-fig2-data1.zip › Fig 2/A/MAX_190116_P6deg_eGFP_pelt2_TIR_Contro_l1-20-to-28.tif]

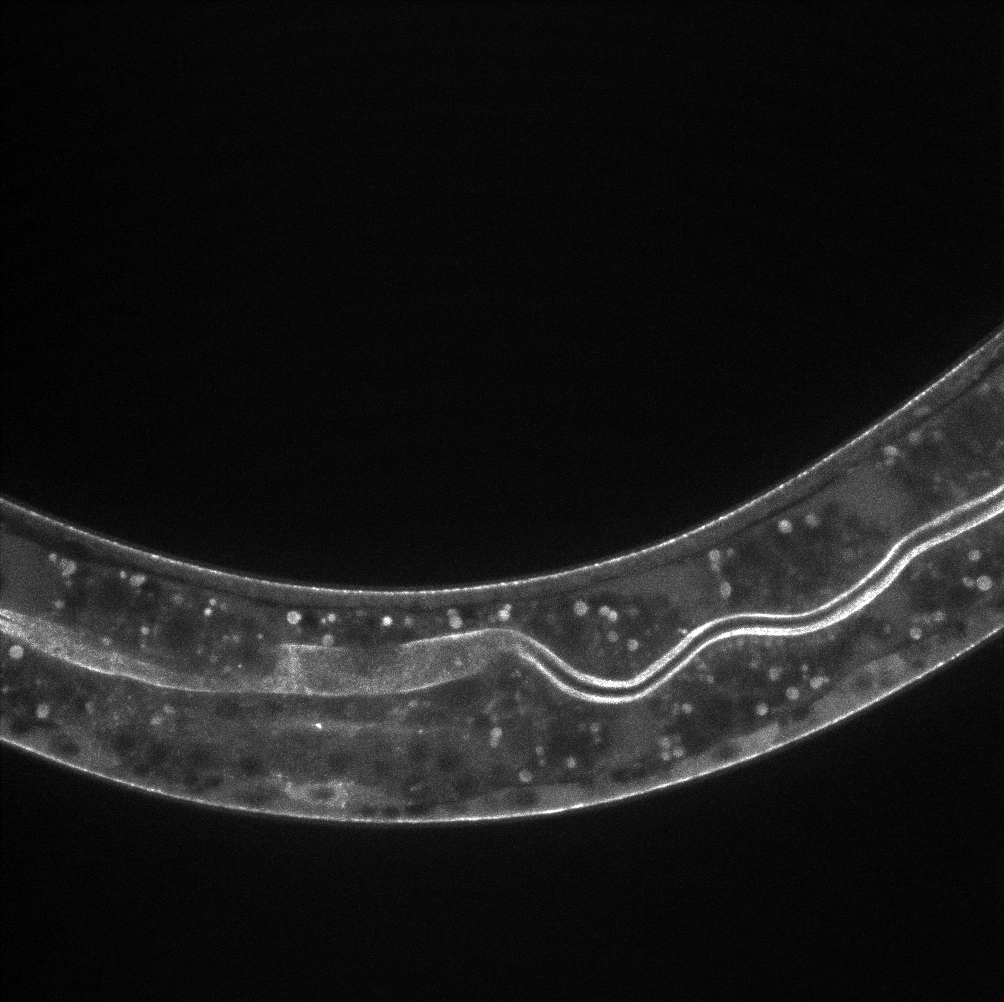

Supplement: Figure 2—source data 1. — This zip archive contains the microscopy images shown in panel A and B, and the data graphed in panels C–J. The images are in TIFF and Adobe Photoshop format. The TIFF file is the unadjusted grayscale maximum intensity projection image generated in ImageJ from an image stack. The Photoshop file contains the original image with the adjustment layers used to arrive at the final image displayed in the main figure. The raw graph data are in Microsoft Excel format, and the summary data and actual graph in Graphpad Prism format. [file elife-62067-fig2-data1.zip › Fig 2/A/MAX_190613_3.18.2.2_Control_3_w1SpinningDisc - Green-18-to-26.tif]

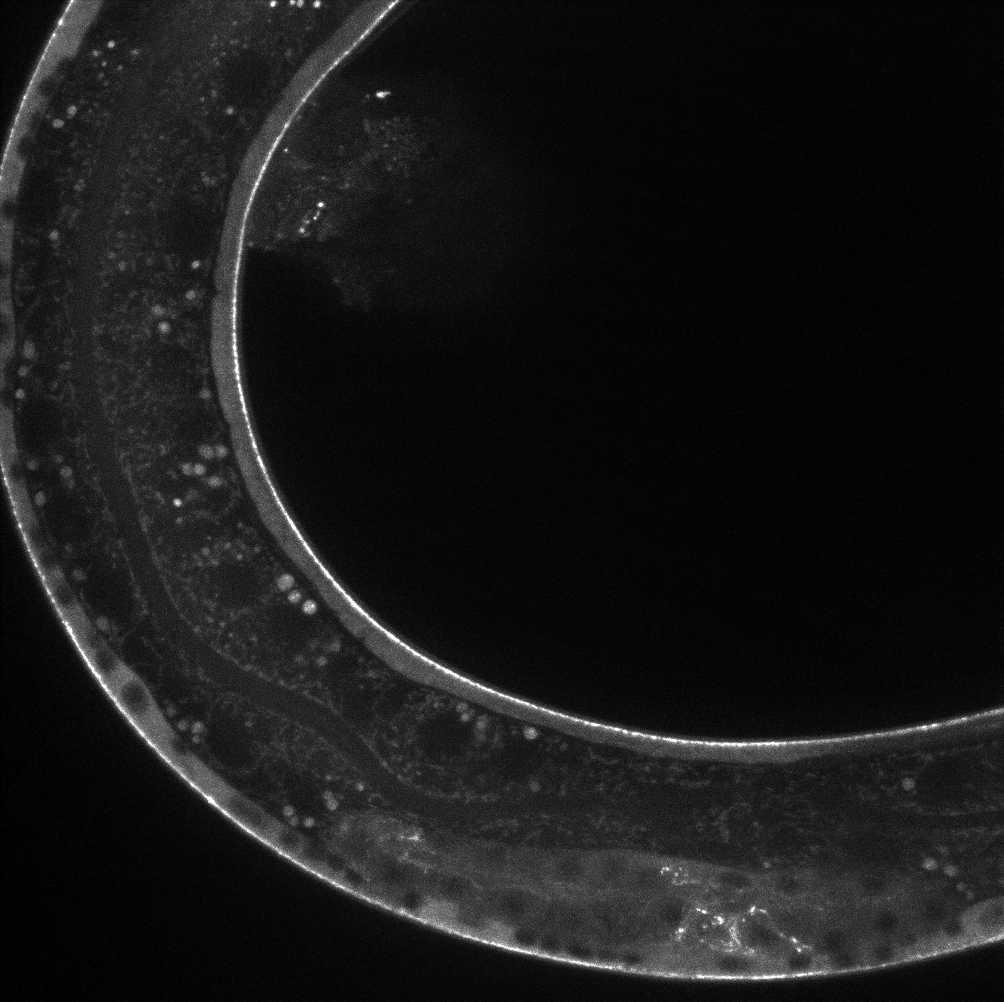

Supplement: Figure 2—source data 1. — This zip archive contains the microscopy images shown in panel A and B, and the data graphed in panels C–J. The images are in TIFF and Adobe Photoshop format. The TIFF file is the unadjusted grayscale maximum intensity projection image generated in ImageJ from an image stack. The Photoshop file contains the original image with the adjustment layers used to arrive at the final image displayed in the main figure. The raw graph data are in Microsoft Excel format, and the summary data and actual graph in Graphpad Prism format. [file elife-62067-fig2-data1.zip › Fig 2/A/MAX_190613_3.18.2.3_Auxin_9_w1SpinningDisc - Green-22-to-30.tif]

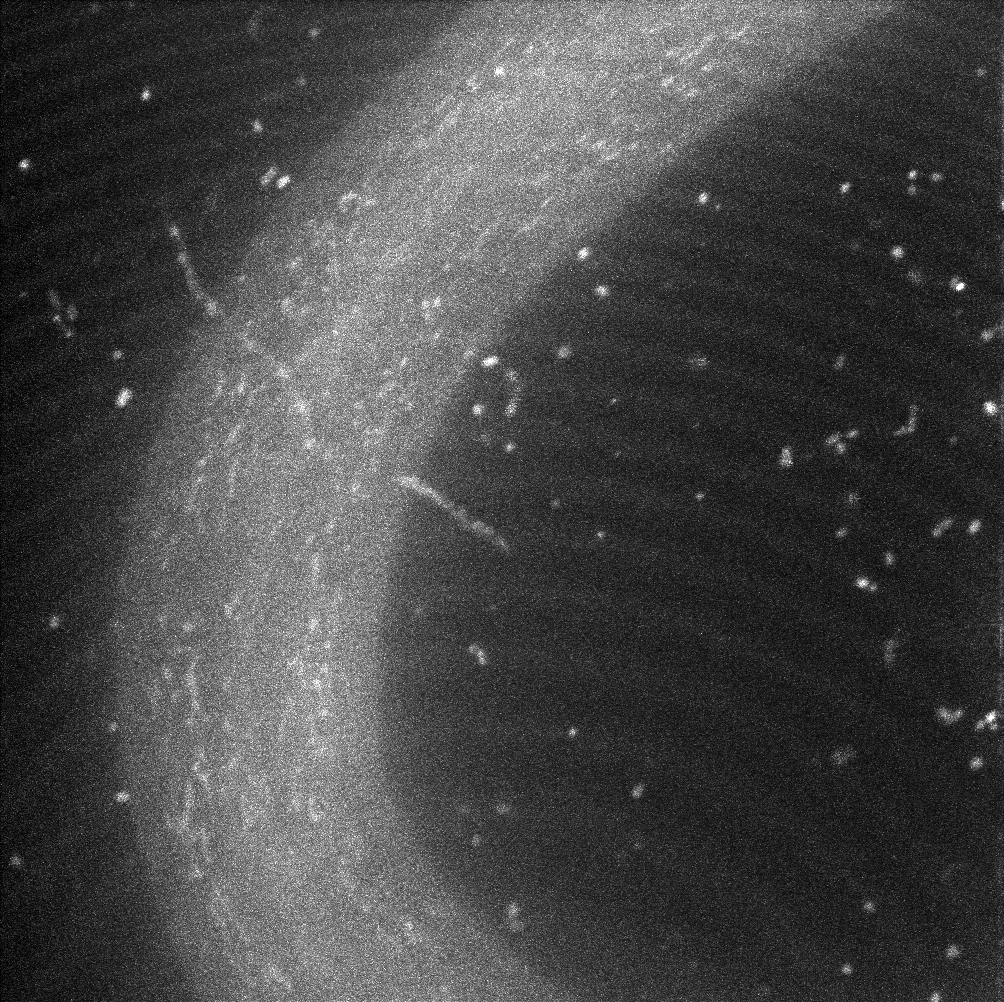

Supplement: Figure 2—source data 1. — This zip archive contains the microscopy images shown in panel A and B, and the data graphed in panels C–J. The images are in TIFF and Adobe Photoshop format. The TIFF file is the unadjusted grayscale maximum intensity projection image generated in ImageJ from an image stack. The Photoshop file contains the original image with the adjustment layers used to arrive at the final image displayed in the main figure. The raw graph data are in Microsoft Excel format, and the summary data and actual graph in Graphpad Prism format. [file elife-62067-fig2-data1.zip › Fig 2/B/MAX_190605_P6deg_ActinRED_L3_AII_4_w1SpinningDisc - Green-8-to-12.tif]

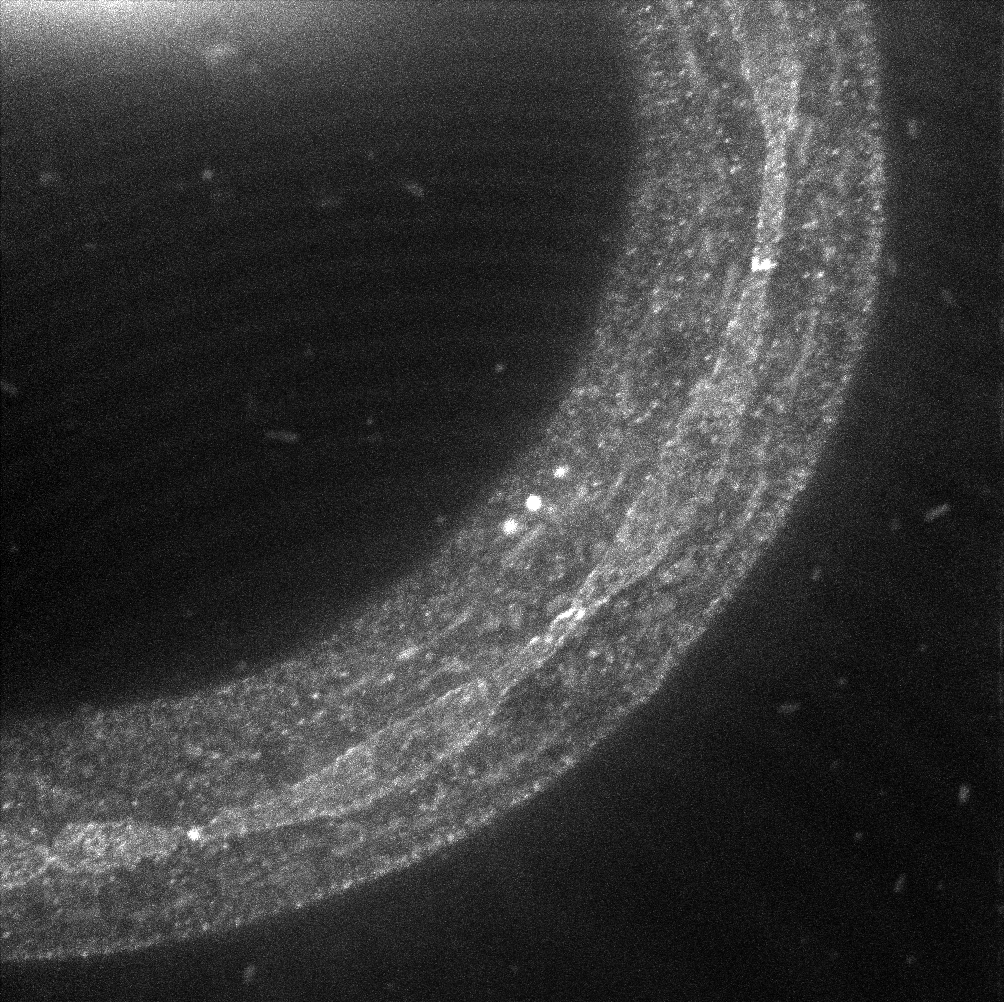

Supplement: Figure 2—source data 1. — This zip archive contains the microscopy images shown in panel A and B, and the data graphed in panels C–J. The images are in TIFF and Adobe Photoshop format. The TIFF file is the unadjusted grayscale maximum intensity projection image generated in ImageJ from an image stack. The Photoshop file contains the original image with the adjustment layers used to arrive at the final image displayed in the main figure. The raw graph data are in Microsoft Excel format, and the summary data and actual graph in Graphpad Prism format. [file elife-62067-fig2-data1.zip › Fig 2/B/MAX_190605_P6deg_ActinRED_L3_CII_8_w1SpinningDisc - Green-8-to-12.tif]

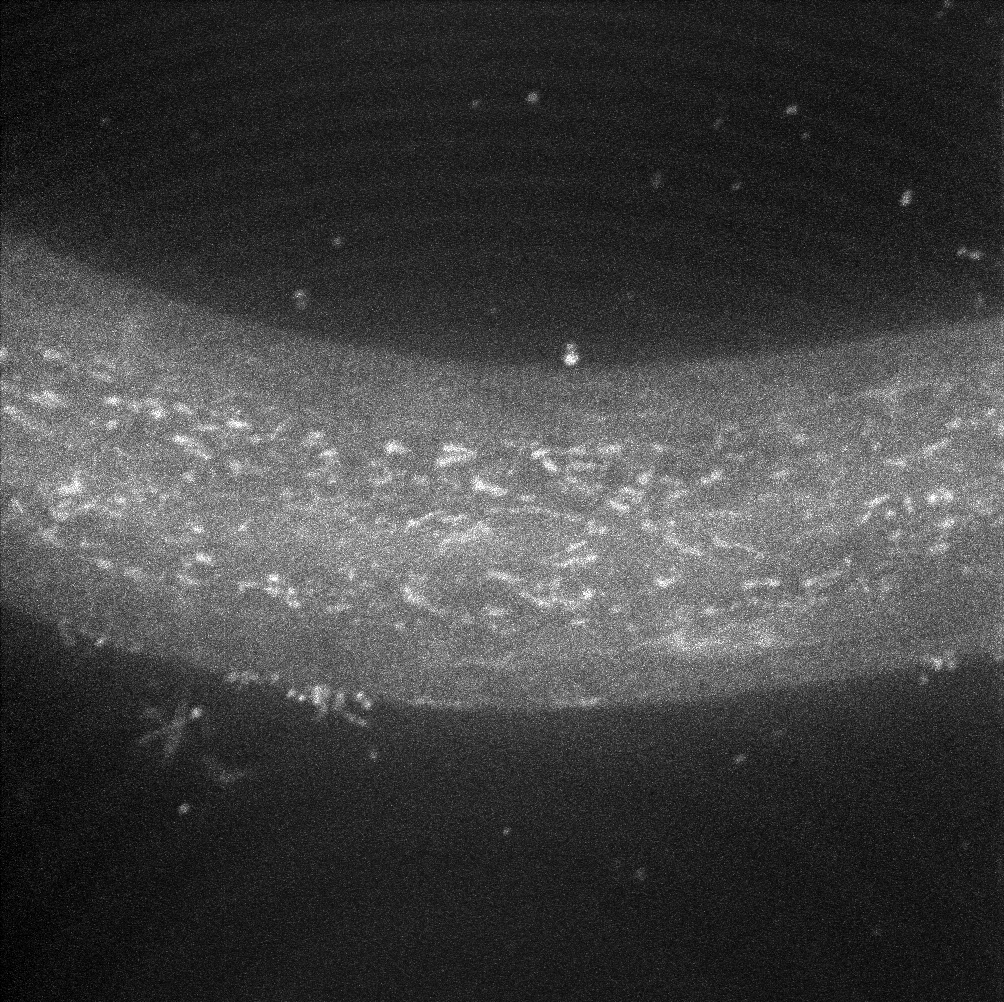

Supplement: Figure 2—source data 1. — This zip archive contains the microscopy images shown in panel A and B, and the data graphed in panels C–J. The images are in TIFF and Adobe Photoshop format. The TIFF file is the unadjusted grayscale maximum intensity projection image generated in ImageJ from an image stack. The Photoshop file contains the original image with the adjustment layers used to arrive at the final image displayed in the main figure. The raw graph data are in Microsoft Excel format, and the summary data and actual graph in Graphpad Prism format. [file elife-62067-fig2-data1.zip › Fig 2/B/MAX_191030_PKC3AID_P6mCh_L3_A_8_w1SpinningDisc - Green-8-to-12.tif]

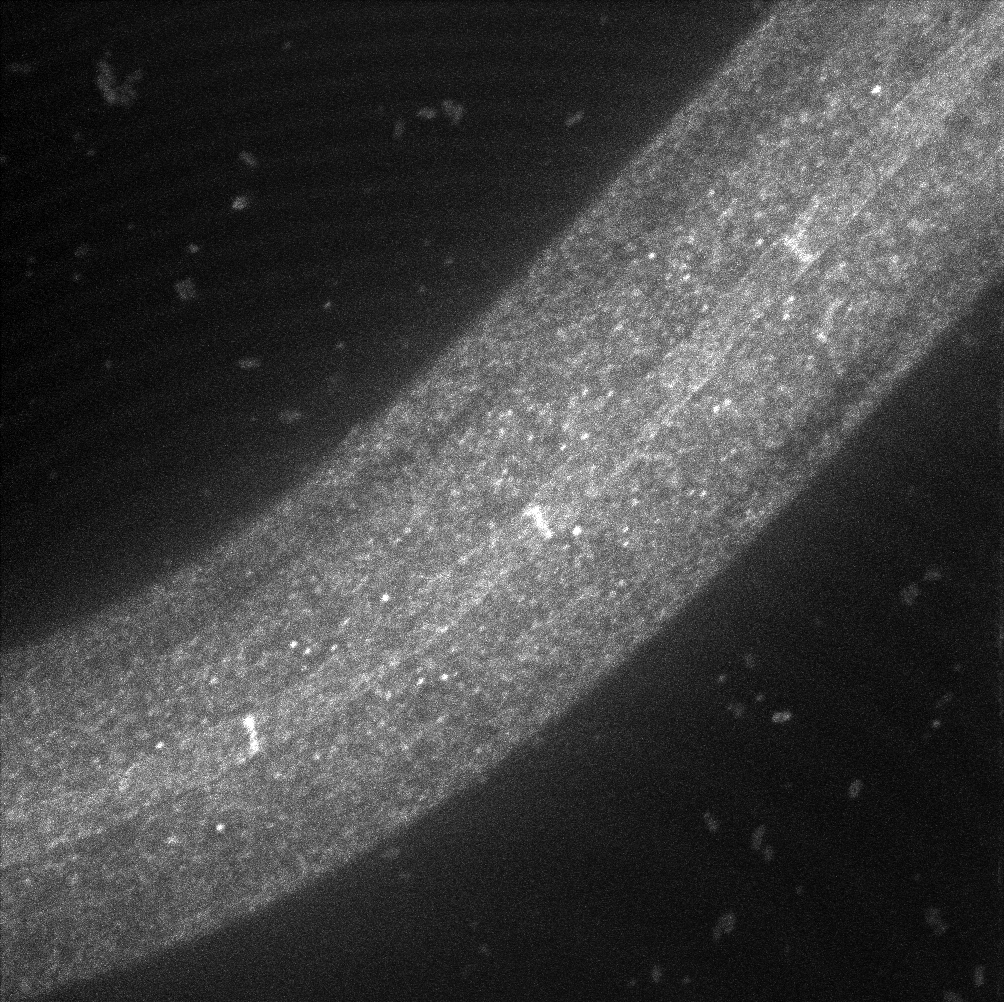

Supplement: Figure 2—source data 1. — This zip archive contains the microscopy images shown in panel A and B, and the data graphed in panels C–J. The images are in TIFF and Adobe Photoshop format. The TIFF file is the unadjusted grayscale maximum intensity projection image generated in ImageJ from an image stack. The Photoshop file contains the original image with the adjustment layers used to arrive at the final image displayed in the main figure. The raw graph data are in Microsoft Excel format, and the summary data and actual graph in Graphpad Prism format. [file elife-62067-fig2-data1.zip › Fig 2/B/MAX_191030_PKC3AID_P6mCh_L3_C_8_w1SpinningDisc - Green-5-to-9.tif]

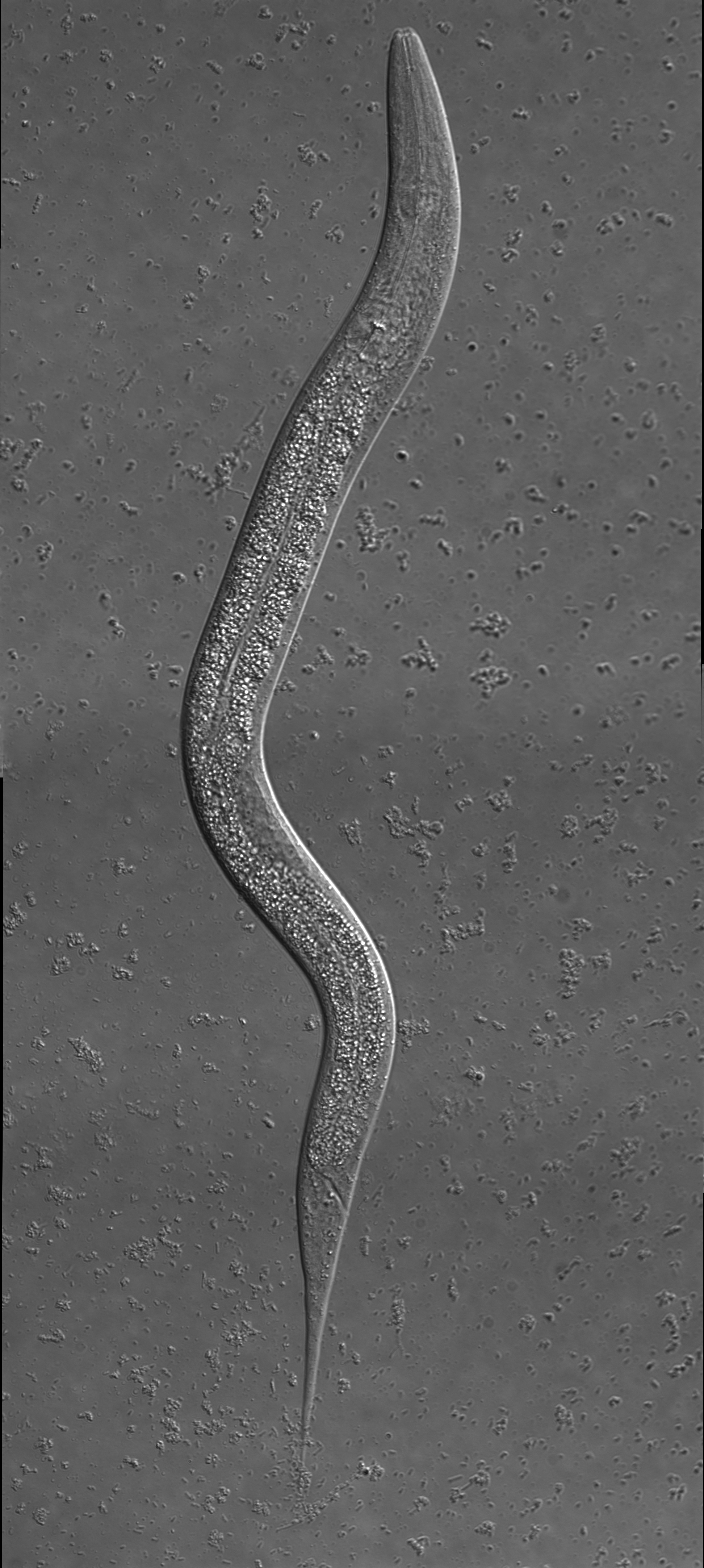

Supplement: Figure 3—figure supplement 2—source data 1. — This zip archive contains the microscopy images shown in panels A, C, and E, and the data graphed in panels B, D, and F. The images are in TIFF and Adobe Photoshop format. The TIFF file is the unadjusted grayscale image (A) or the unadjusted grayscale maximum intensity projection image generated in ImageJ from an image stack (C and E). The Photoshop file contains the original image with the adjustment layers used to arrive at the final image displayed in the main figure. The raw graph data are in Microsoft Excel format, and the summary data and actual graph in Graphpad Prism format. [file elife-62067-fig3-figsupp2-data1.zip › Fig 3S2/A/200319_P6aid_Pdpy7_2-to-5_rescued.tif]

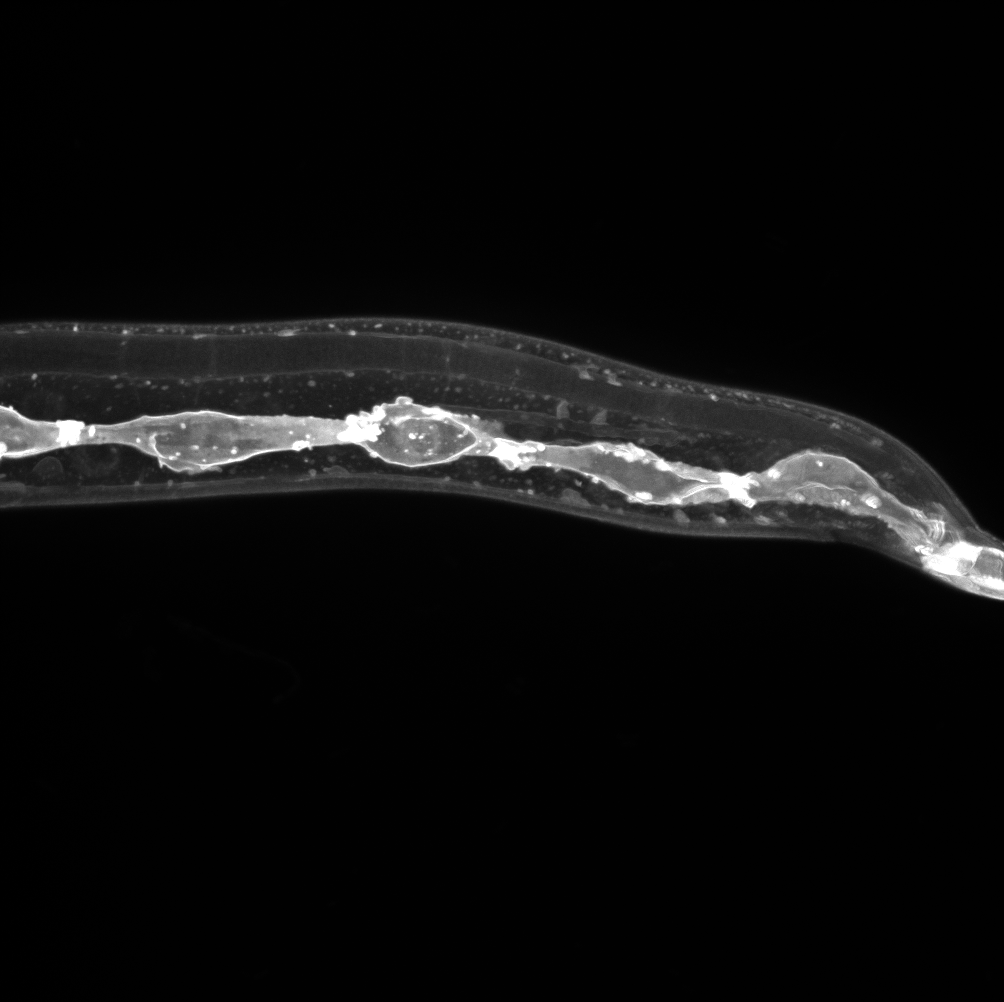

Supplement: Figure 3—figure supplement 2—source data 1. — This zip archive contains the microscopy images shown in panels A, C, and E, and the data graphed in panels B, D, and F. The images are in TIFF and Adobe Photoshop format. The TIFF file is the unadjusted grayscale image (A) or the unadjusted grayscale maximum intensity projection image generated in ImageJ from an image stack (C and E). The Photoshop file contains the original image with the adjustment layers used to arrive at the final image displayed in the main figure. The raw graph data are in Microsoft Excel format, and the summary data and actual graph in Graphpad Prism format. [file elife-62067-fig3-figsupp2-data1.zip › Fig 3S2/C/MAX_200124_P6aid_Pdpy7par6_rescue_A2_1_w1SpinningDisc - Green_s3-1-to-6.tif]

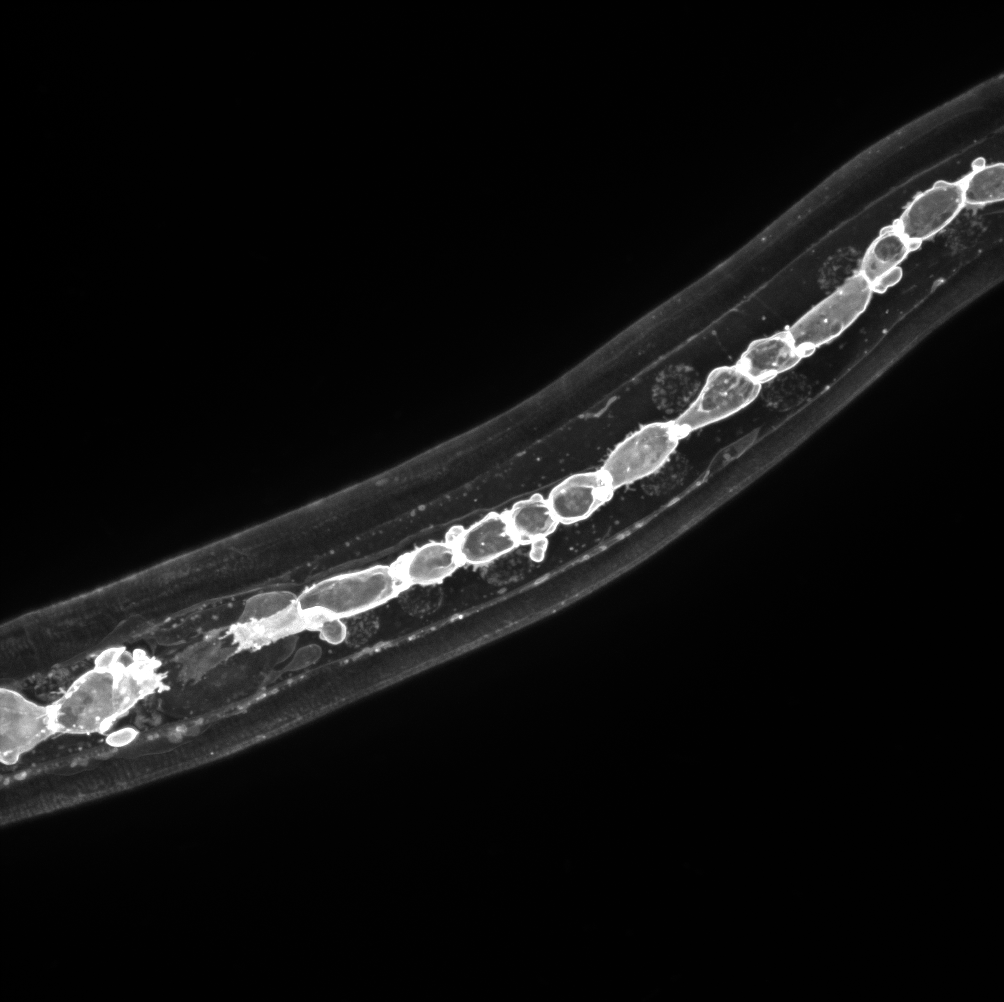

Supplement: Figure 3—figure supplement 2—source data 1. — This zip archive contains the microscopy images shown in panels A, C, and E, and the data graphed in panels B, D, and F. The images are in TIFF and Adobe Photoshop format. The TIFF file is the unadjusted grayscale image (A) or the unadjusted grayscale maximum intensity projection image generated in ImageJ from an image stack (C and E). The Photoshop file contains the original image with the adjustment layers used to arrive at the final image displayed in the main figure. The raw graph data are in Microsoft Excel format, and the summary data and actual graph in Graphpad Prism format. [file elife-62067-fig3-figsupp2-data1.zip › Fig 3S2/C/MAX_200124_P6aid_Pdpy7par6_rescue_A3_2_w1SpinningDisc - Green_s3-1-to-8.tif]

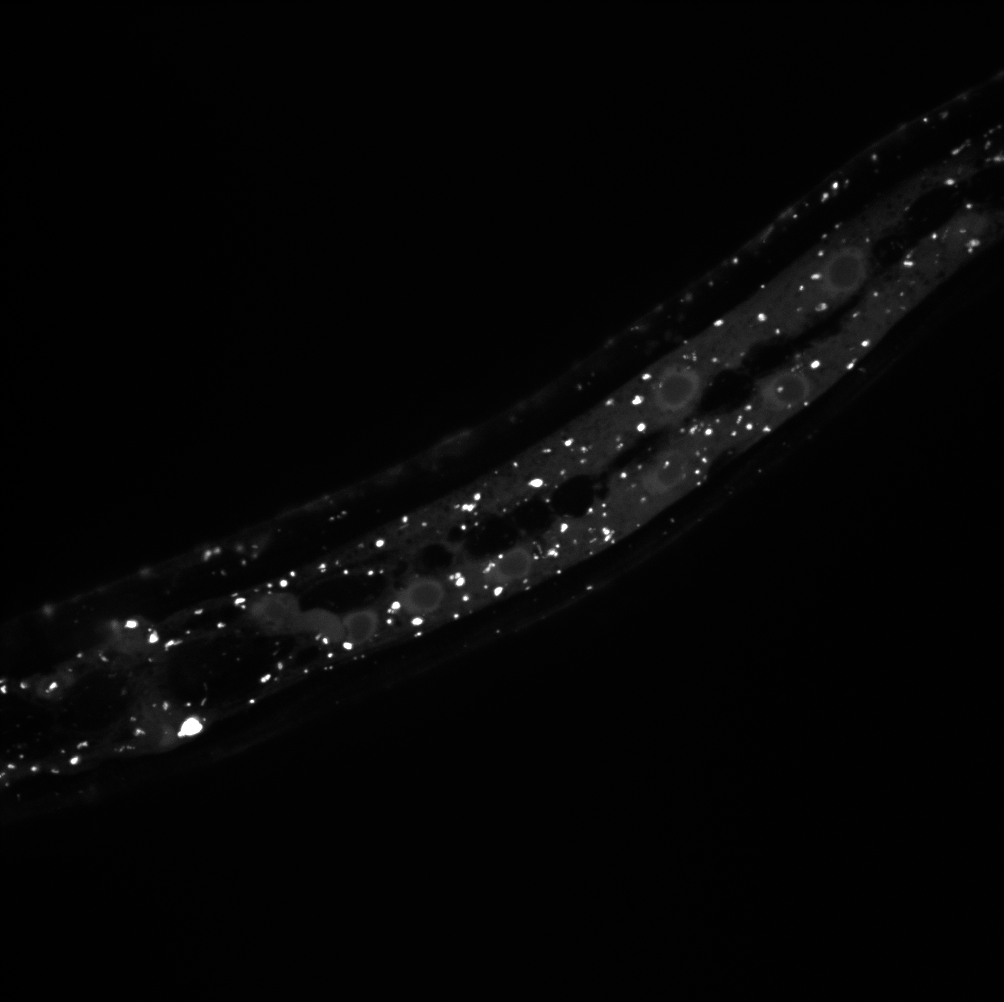

Supplement: Figure 3—figure supplement 2—source data 1. — This zip archive contains the microscopy images shown in panels A, C, and E, and the data graphed in panels B, D, and F. The images are in TIFF and Adobe Photoshop format. The TIFF file is the unadjusted grayscale image (A) or the unadjusted grayscale maximum intensity projection image generated in ImageJ from an image stack (C and E). The Photoshop file contains the original image with the adjustment layers used to arrive at the final image displayed in the main figure. The raw graph data are in Microsoft Excel format, and the summary data and actual graph in Graphpad Prism format. [file elife-62067-fig3-figsupp2-data1.zip › Fig 3S2/C/MAX_200124_P6aid_Pdpy7par6_rescue_A3_2_w2SpinningDisc - Red_s3-1-to-8.tif]

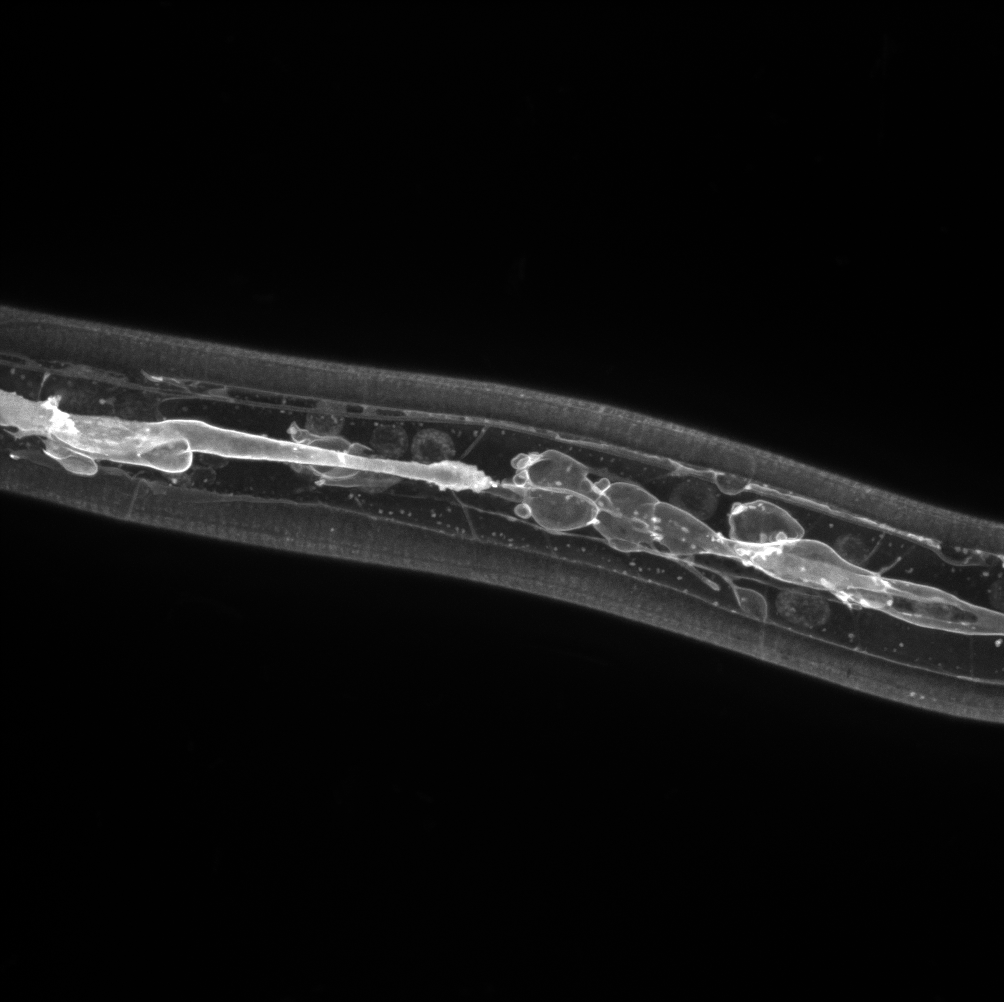

Supplement: Figure 3—figure supplement 2—source data 1. — This zip archive contains the microscopy images shown in panels A, C, and E, and the data graphed in panels B, D, and F. The images are in TIFF and Adobe Photoshop format. The TIFF file is the unadjusted grayscale image (A) or the unadjusted grayscale maximum intensity projection image generated in ImageJ from an image stack (C and E). The Photoshop file contains the original image with the adjustment layers used to arrive at the final image displayed in the main figure. The raw graph data are in Microsoft Excel format, and the summary data and actual graph in Graphpad Prism format. [file elife-62067-fig3-figsupp2-data1.zip › Fig 3S2/C/MAX_200124_P6aid_Pdpy7par6_rescue_A4_2_w1SpinningDisc - Green_s2-1-to-8.tif]

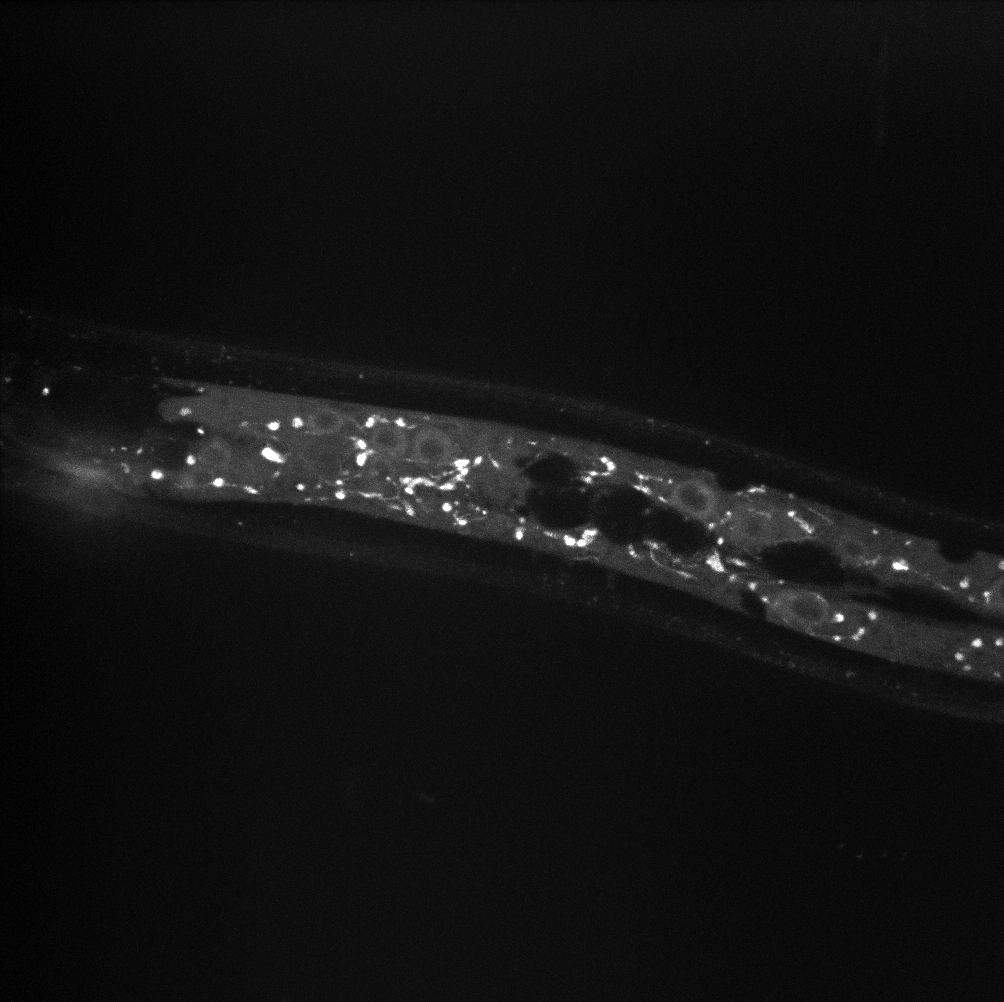

Supplement: Figure 3—figure supplement 2—source data 1. — This zip archive contains the microscopy images shown in panels A, C, and E, and the data graphed in panels B, D, and F. The images are in TIFF and Adobe Photoshop format. The TIFF file is the unadjusted grayscale image (A) or the unadjusted grayscale maximum intensity projection image generated in ImageJ from an image stack (C and E). The Photoshop file contains the original image with the adjustment layers used to arrive at the final image displayed in the main figure. The raw graph data are in Microsoft Excel format, and the summary data and actual graph in Graphpad Prism format. [file elife-62067-fig3-figsupp2-data1.zip › Fig 3S2/C/MAX_200124_P6aid_Pdpy7par6_rescue_A4_2_w2SpinningDisc - Red_s2-1-to-8.tif]

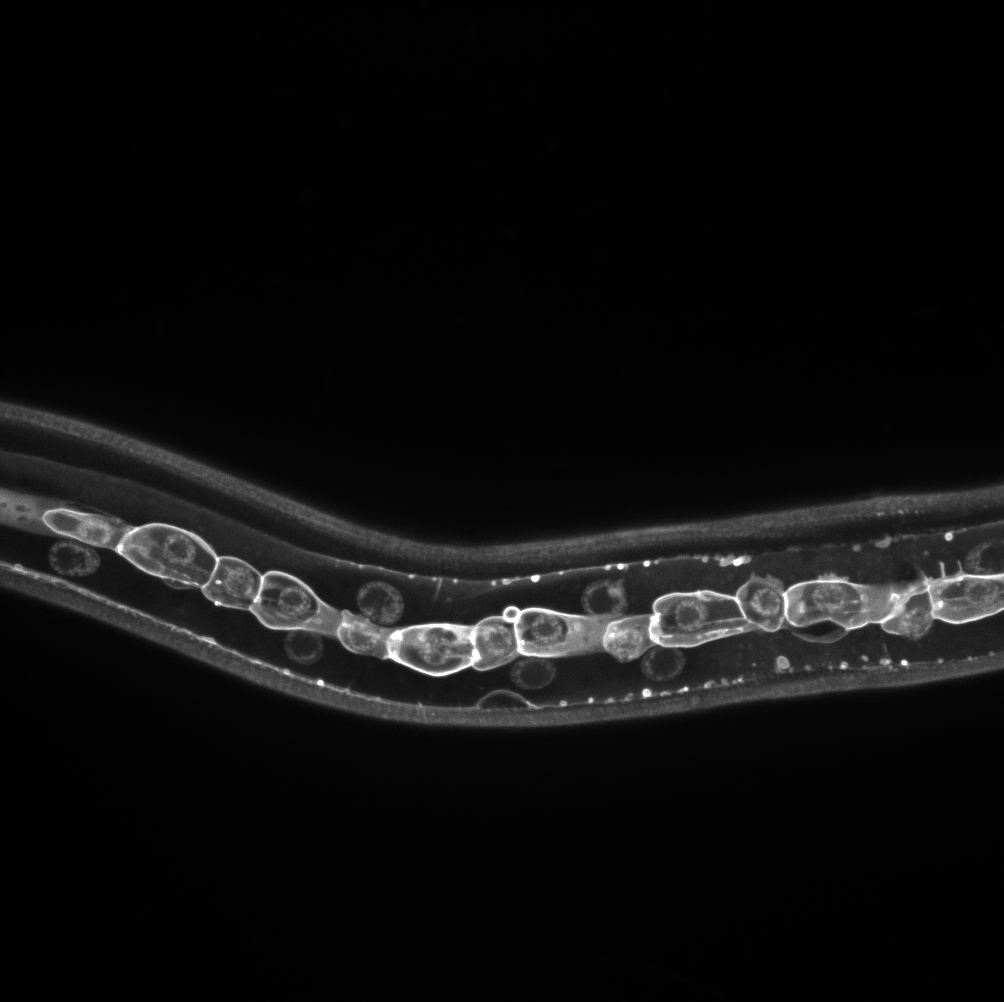

Supplement: Figure 3—figure supplement 2—source data 1. — This zip archive contains the microscopy images shown in panels A, C, and E, and the data graphed in panels B, D, and F. The images are in TIFF and Adobe Photoshop format. The TIFF file is the unadjusted grayscale image (A) or the unadjusted grayscale maximum intensity projection image generated in ImageJ from an image stack (C and E). The Photoshop file contains the original image with the adjustment layers used to arrive at the final image displayed in the main figure. The raw graph data are in Microsoft Excel format, and the summary data and actual graph in Graphpad Prism format. [file elife-62067-fig3-figsupp2-data1.zip › Fig 3S2/C/MAX_200124_P6aid_Pdpy7par6_rescue_C_SV_2_s2.tif]

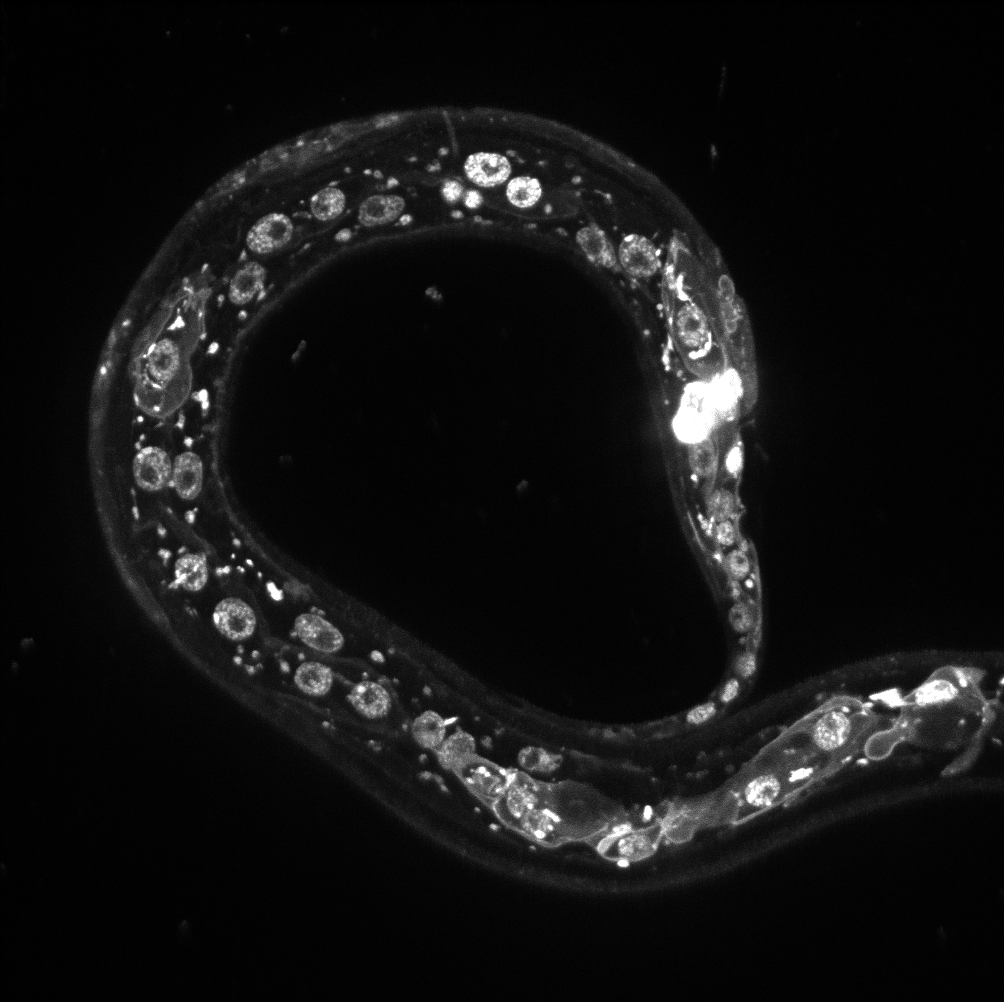

Supplement: Figure 3—figure supplement 2—source data 1. — This zip archive contains the microscopy images shown in panels A, C, and E, and the data graphed in panels B, D, and F. The images are in TIFF and Adobe Photoshop format. The TIFF file is the unadjusted grayscale image (A) or the unadjusted grayscale maximum intensity projection image generated in ImageJ from an image stack (C and E). The Photoshop file contains the original image with the adjustment layers used to arrive at the final image displayed in the main figure. The raw graph data are in Microsoft Excel format, and the summary data and actual graph in Graphpad Prism format. [file elife-62067-fig3-figsupp2-data1.zip › Fig 3S2/E/MAX_200609_neklaid_chc1gfp_seam_L2_A_20-1-to-6.tif]

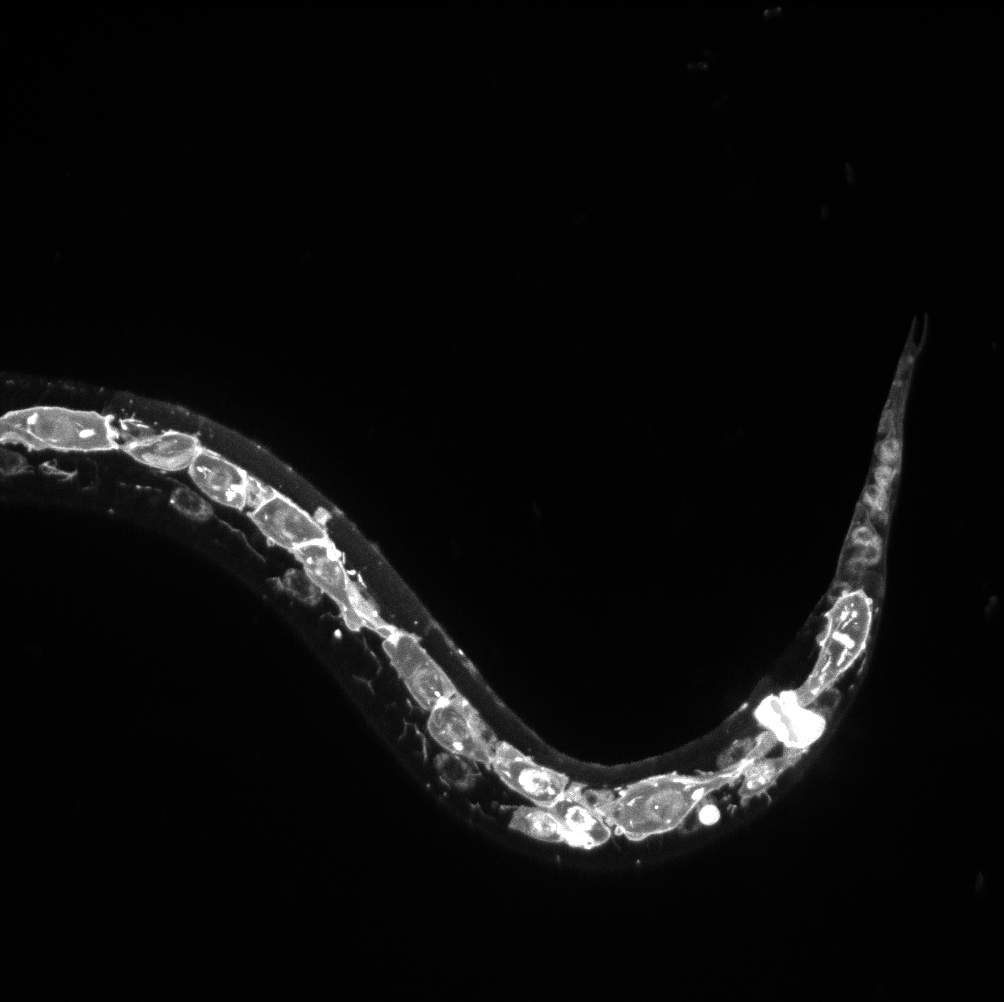

Supplement: Figure 3—figure supplement 2—source data 1. — This zip archive contains the microscopy images shown in panels A, C, and E, and the data graphed in panels B, D, and F. The images are in TIFF and Adobe Photoshop format. The TIFF file is the unadjusted grayscale image (A) or the unadjusted grayscale maximum intensity projection image generated in ImageJ from an image stack (C and E). The Photoshop file contains the original image with the adjustment layers used to arrive at the final image displayed in the main figure. The raw graph data are in Microsoft Excel format, and the summary data and actual graph in Graphpad Prism format. [file elife-62067-fig3-figsupp2-data1.zip › Fig 3S2/E/MAX_200609_neklaid_chc1gfp_seam_L2_A_28-2-to-9.tif]

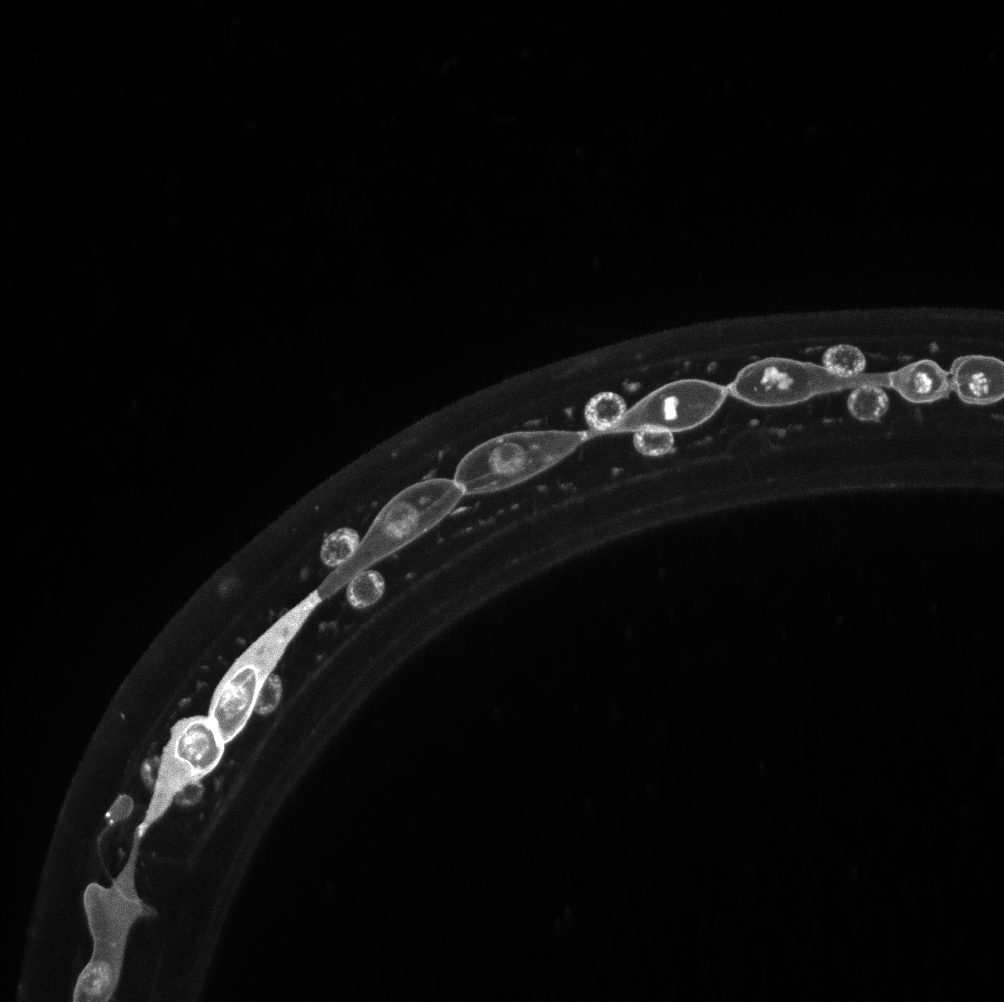

Supplement: Figure 3—figure supplement 2—source data 1. — This zip archive contains the microscopy images shown in panels A, C, and E, and the data graphed in panels B, D, and F. The images are in TIFF and Adobe Photoshop format. The TIFF file is the unadjusted grayscale image (A) or the unadjusted grayscale maximum intensity projection image generated in ImageJ from an image stack (C and E). The Photoshop file contains the original image with the adjustment layers used to arrive at the final image displayed in the main figure. The raw graph data are in Microsoft Excel format, and the summary data and actual graph in Graphpad Prism format. [file elife-62067-fig3-figsupp2-data1.zip › Fig 3S2/E/MAX_200609_neklaid_chc1gfp_seam_L2_C_26-3-to-12.tif]

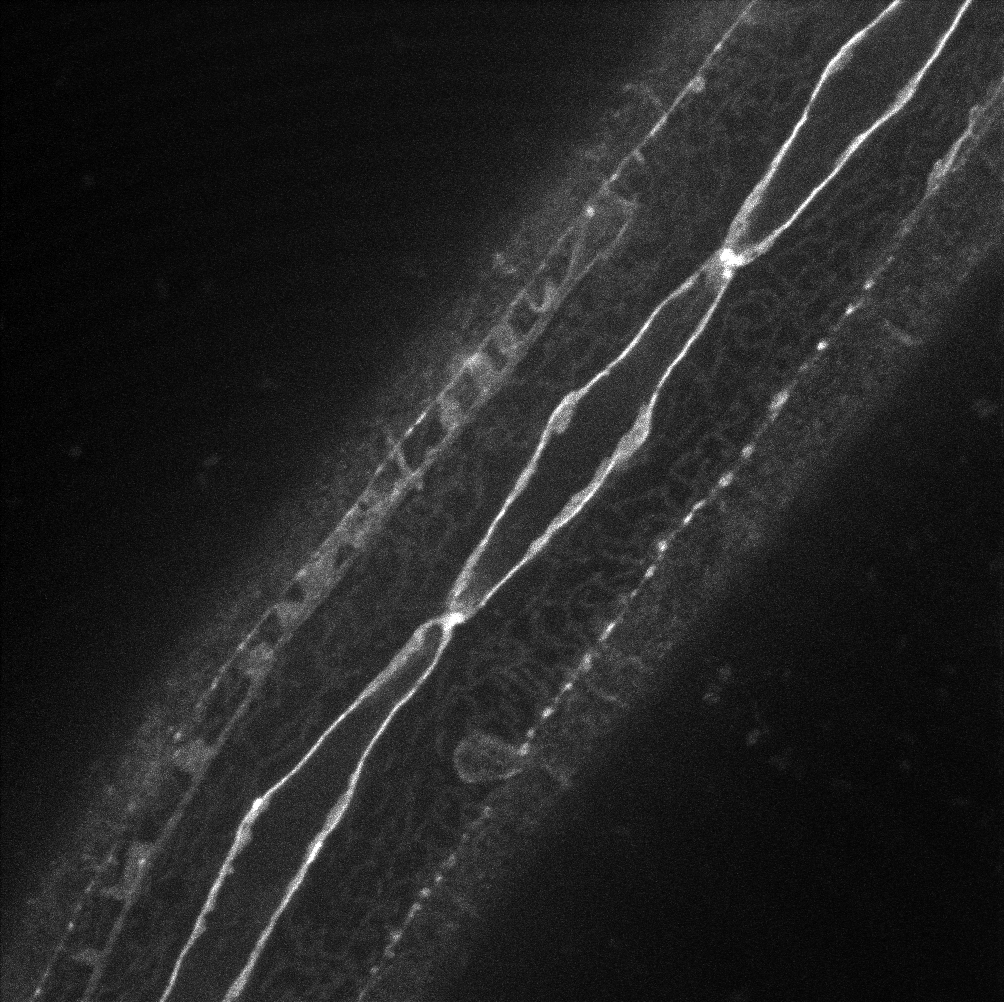

Supplement: Figure 4—source data 1. — This zip archive contains the microscopy images shown in panels A, C, E, and F, and the data graphed in panels B, D. The images are in TIFF and Adobe Photoshop format. The TIFF file is the unadjusted grayscale maximum intensity projection image generated in ImageJ from an image stack. The Photoshop file contains the original image with the adjustment layers used to arrive at the final image displayed in the main figure. The raw graph data are in Microsoft Excel format, and the summary data and actual graph in Graphpad Prism format. [file elife-62067-fig4-data1.zip › Fig 4/A/MAX_200205_lgl1gfp_L3_C_13-16-to-18.tif]

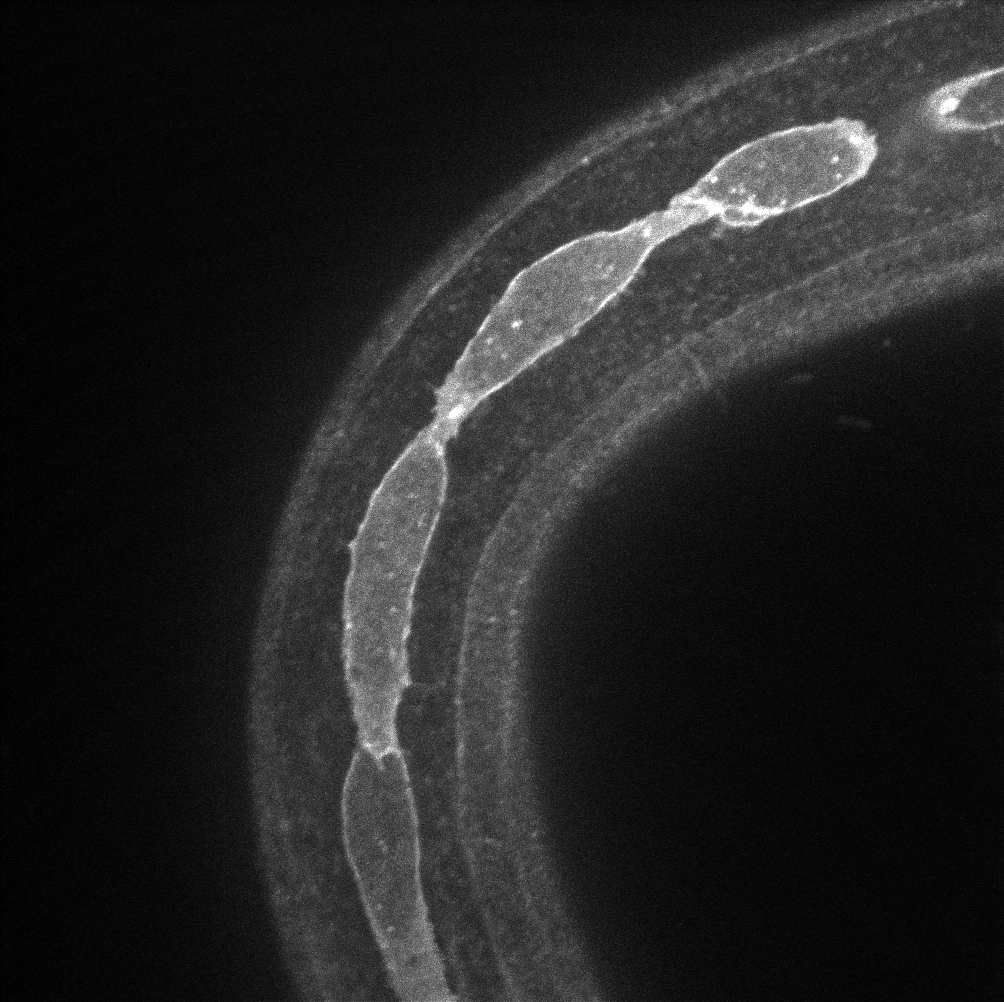

Supplement: Figure 4—source data 1. — This zip archive contains the microscopy images shown in panels A, C, E, and F, and the data graphed in panels B, D. The images are in TIFF and Adobe Photoshop format. The TIFF file is the unadjusted grayscale maximum intensity projection image generated in ImageJ from an image stack. The Photoshop file contains the original image with the adjustment layers used to arrive at the final image displayed in the main figure. The raw graph data are in Microsoft Excel format, and the summary data and actual graph in Graphpad Prism format. [file elife-62067-fig4-data1.zip › Fig 4/A/MAX_200211_pkc3aid_lgl1gfp_L3_A_7h_3-17-to-19.tif]

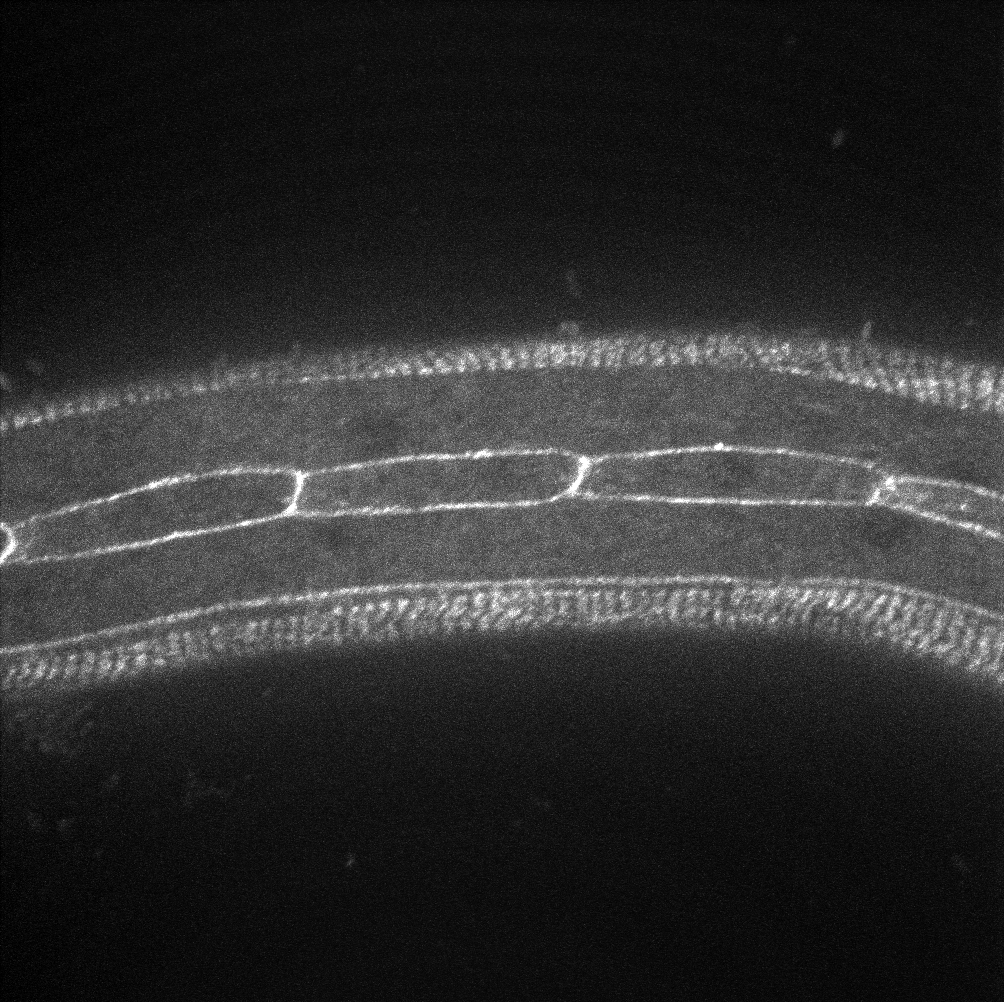

Supplement: Figure 4—source data 1. — This zip archive contains the microscopy images shown in panels A, C, E, and F, and the data graphed in panels B, D. The images are in TIFF and Adobe Photoshop format. The TIFF file is the unadjusted grayscale maximum intensity projection image generated in ImageJ from an image stack. The Photoshop file contains the original image with the adjustment layers used to arrive at the final image displayed in the main figure. The raw graph data are in Microsoft Excel format, and the summary data and actual graph in Graphpad Prism format. [file elife-62067-fig4-data1.zip › Fig 4/C/MAX_200211_par1gfp_L3_C_2-15-to-17.tif]

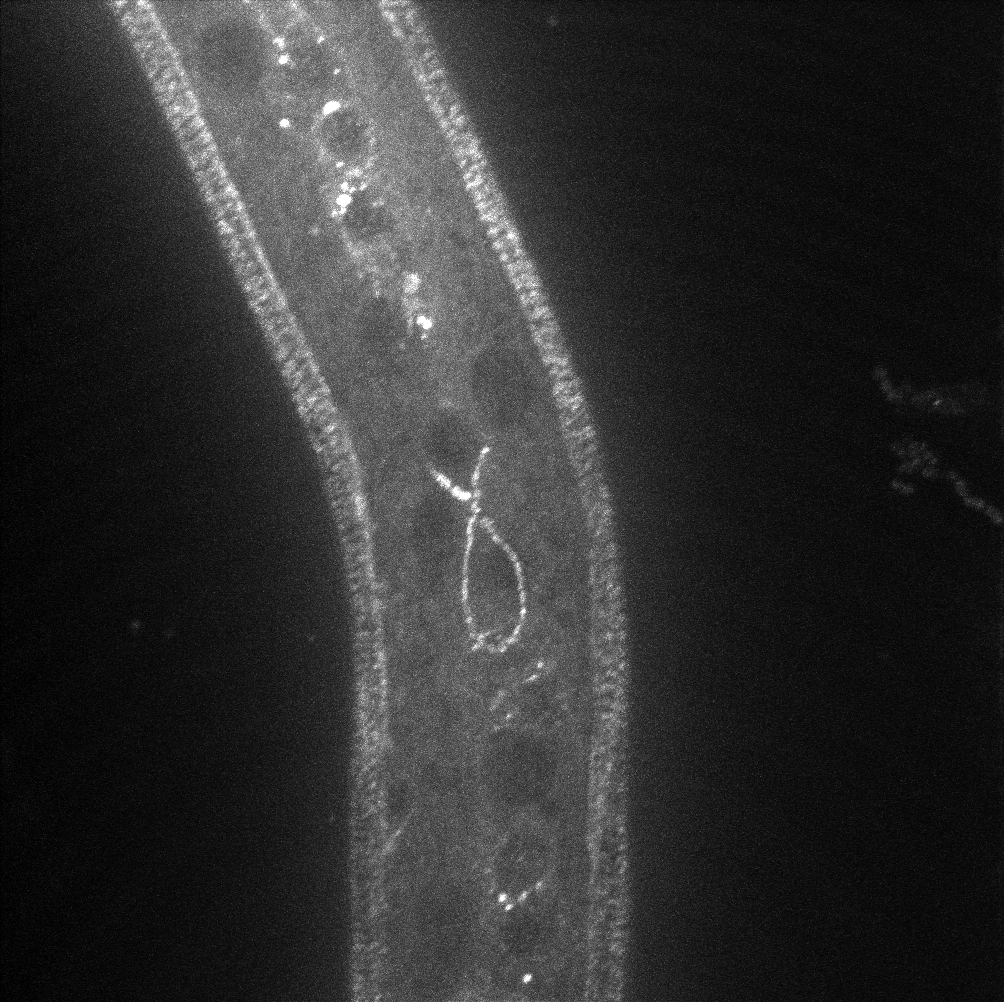

Supplement: Figure 4—source data 1. — This zip archive contains the microscopy images shown in panels A, C, E, and F, and the data graphed in panels B, D. The images are in TIFF and Adobe Photoshop format. The TIFF file is the unadjusted grayscale maximum intensity projection image generated in ImageJ from an image stack. The Photoshop file contains the original image with the adjustment layers used to arrive at the final image displayed in the main figure. The raw graph data are in Microsoft Excel format, and the summary data and actual graph in Graphpad Prism format. [file elife-62067-fig4-data1.zip › Fig 4/C/MAX_200211_pkc3aid_par1gfp_L3_A_24h_7-13-to-17.tif]

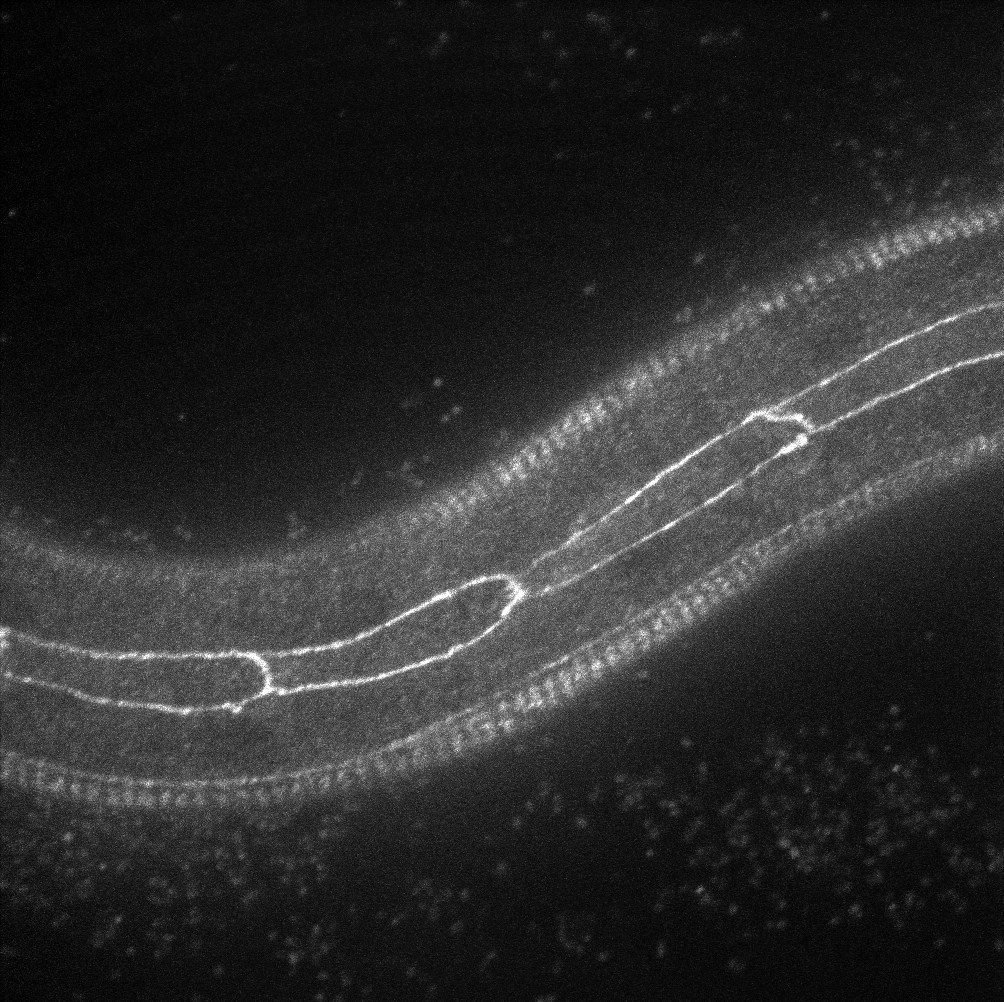

Supplement: Figure 4—source data 1. — This zip archive contains the microscopy images shown in panels A, C, E, and F, and the data graphed in panels B, D. The images are in TIFF and Adobe Photoshop format. The TIFF file is the unadjusted grayscale maximum intensity projection image generated in ImageJ from an image stack. The Photoshop file contains the original image with the adjustment layers used to arrive at the final image displayed in the main figure. The raw graph data are in Microsoft Excel format, and the summary data and actual graph in Graphpad Prism format. [file elife-62067-fig4-data1.zip › Fig 4/C/MAX_200211_pkc3aid_par1gfp_L3_A_7h_3-16-to-18.tif]

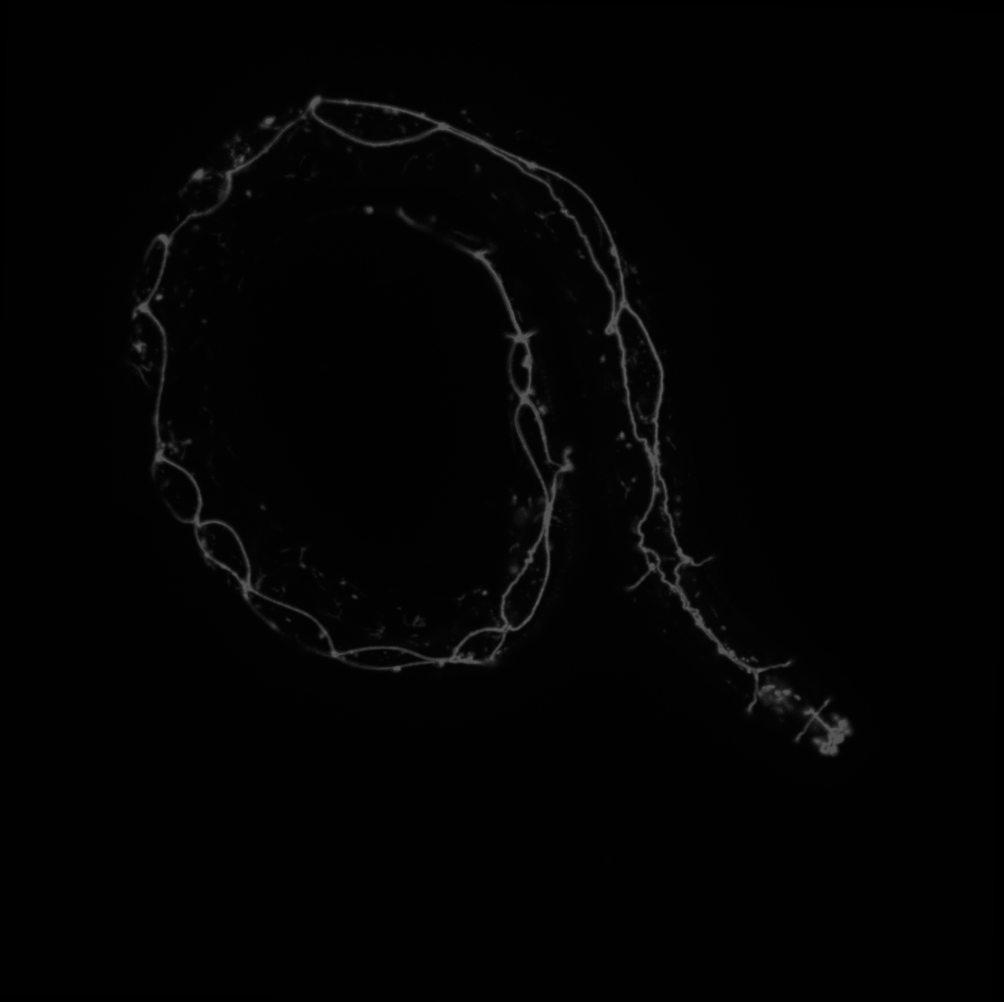

Supplement: Figure 4—source data 1. — This zip archive contains the microscopy images shown in panels A, C, E, and F, and the data graphed in panels B, D. The images are in TIFF and Adobe Photoshop format. The TIFF file is the unadjusted grayscale maximum intensity projection image generated in ImageJ from an image stack. The Photoshop file contains the original image with the adjustment layers used to arrive at the final image displayed in the main figure. The raw graph data are in Microsoft Excel format, and the summary data and actual graph in Graphpad Prism format. [file elife-62067-fig4-data1.zip › Fig 4/E/190812_PKC3_deg_dlg1_mCh_L1_A_27_5-to-6.tif]

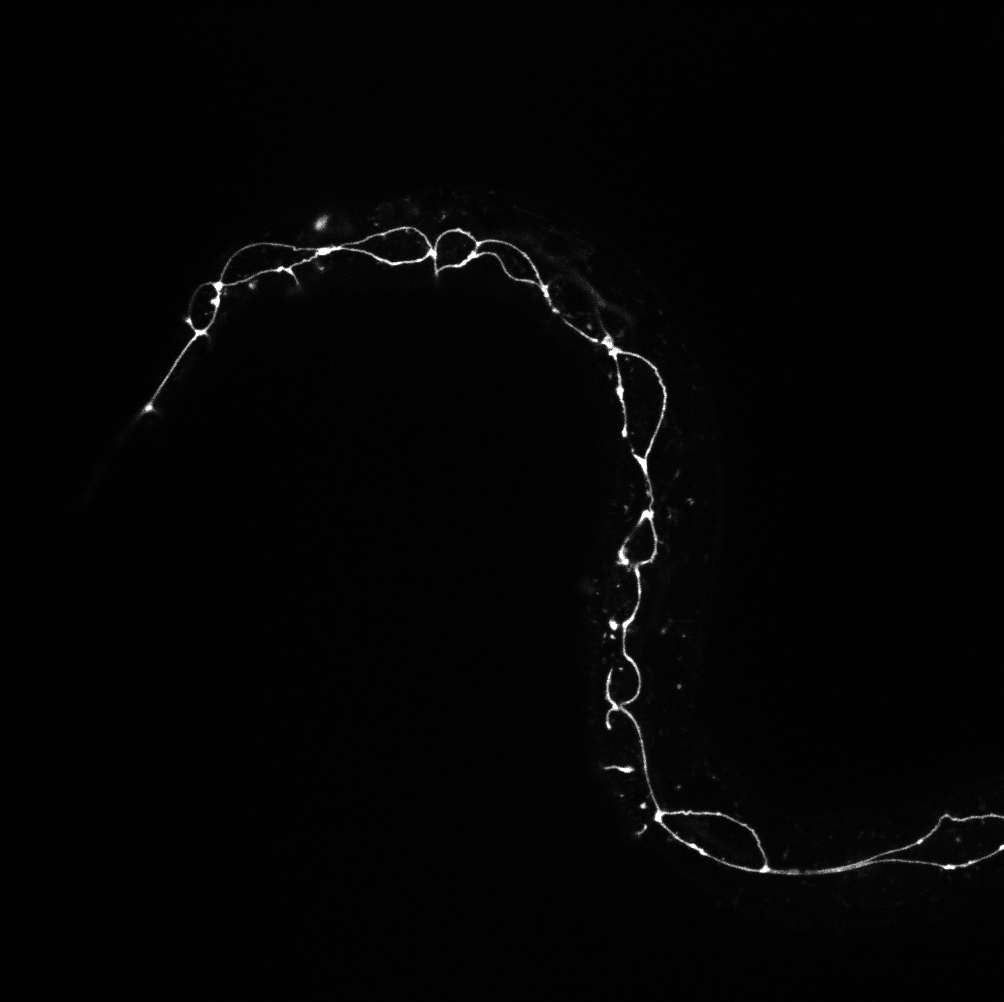

Supplement: Figure 4—source data 1. — This zip archive contains the microscopy images shown in panels A, C, E, and F, and the data graphed in panels B, D. The images are in TIFF and Adobe Photoshop format. The TIFF file is the unadjusted grayscale maximum intensity projection image generated in ImageJ from an image stack. The Photoshop file contains the original image with the adjustment layers used to arrive at the final image displayed in the main figure. The raw graph data are in Microsoft Excel format, and the summary data and actual graph in Graphpad Prism format. [file elife-62067-fig4-data1.zip › Fig 4/E/MAX_280119_P6deg_pwrt2TIR_DLGmCh_A5h_3_w2SpinningDisc - Red-6.tif]

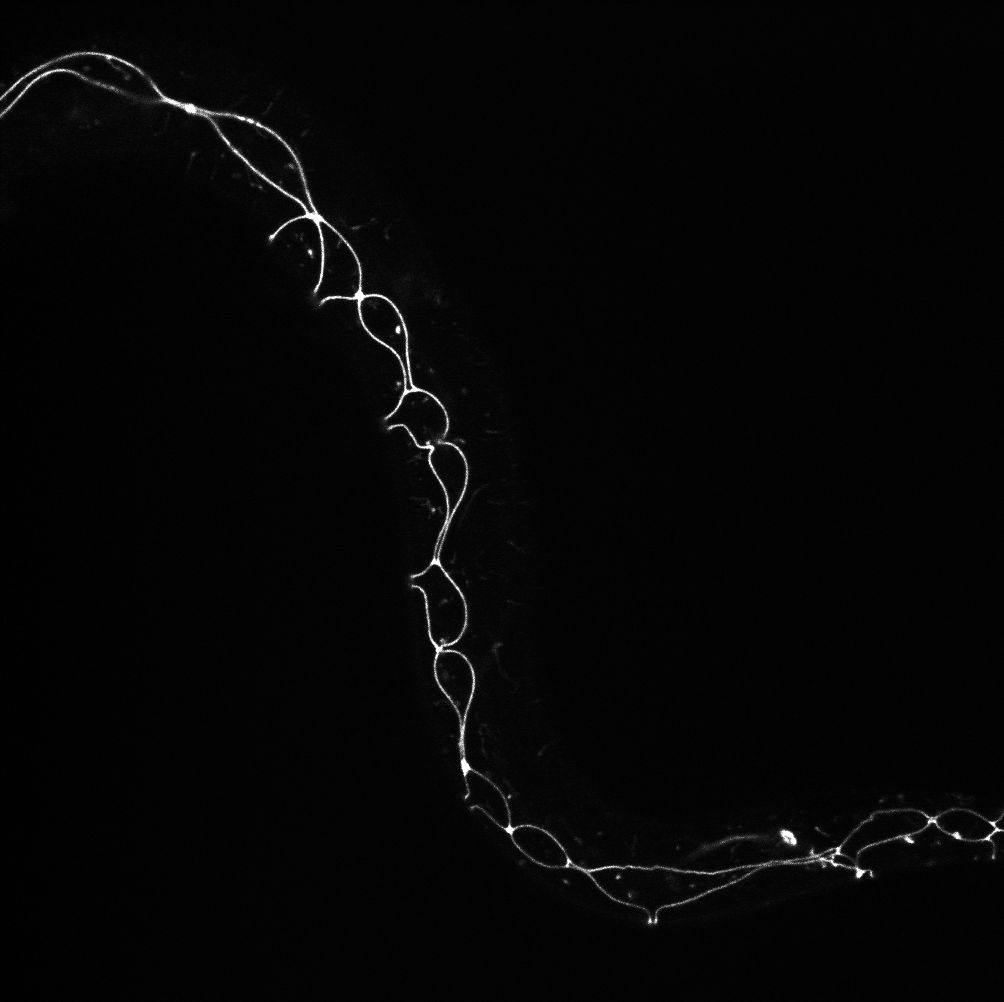

Supplement: Figure 4—source data 1. — This zip archive contains the microscopy images shown in panels A, C, E, and F, and the data graphed in panels B, D. The images are in TIFF and Adobe Photoshop format. The TIFF file is the unadjusted grayscale maximum intensity projection image generated in ImageJ from an image stack. The Photoshop file contains the original image with the adjustment layers used to arrive at the final image displayed in the main figure. The raw graph data are in Microsoft Excel format, and the summary data and actual graph in Graphpad Prism format. [file elife-62067-fig4-data1.zip › Fig 4/E/MAX_280119_P6deg_pwrt2TIR_DLGmCh_C4.30h_16_w2SpinningDisc - Red-7.tif]

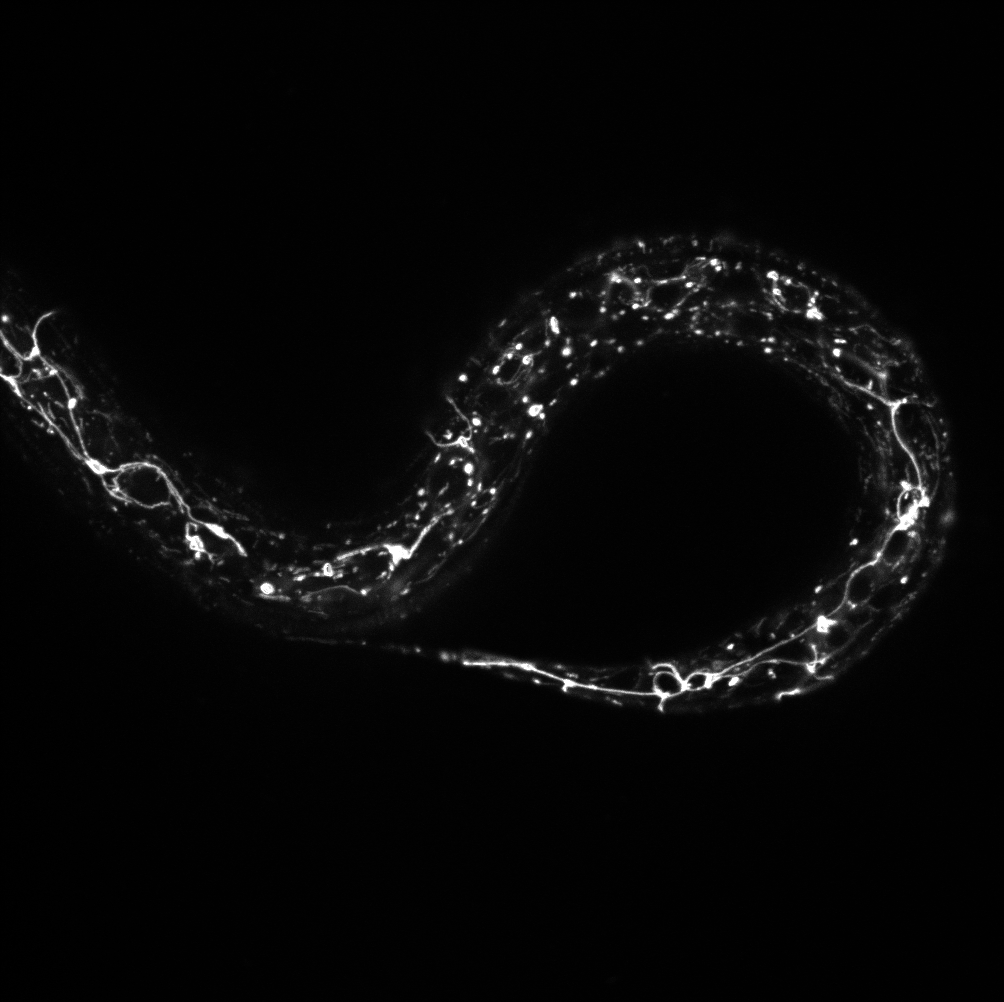

Supplement: Figure 4—source data 1. — This zip archive contains the microscopy images shown in panels A, C, E, and F, and the data graphed in panels B, D. The images are in TIFF and Adobe Photoshop format. The TIFF file is the unadjusted grayscale maximum intensity projection image generated in ImageJ from an image stack. The Photoshop file contains the original image with the adjustment layers used to arrive at the final image displayed in the main figure. The raw graph data are in Microsoft Excel format, and the summary data and actual graph in Graphpad Prism format. [file elife-62067-fig4-data1.zip › Fig 4/F/MAX_190806_PKC3_deg_dlg1_mCh_A_55_w1SpinningDisc - Red-1-to-3.tif]

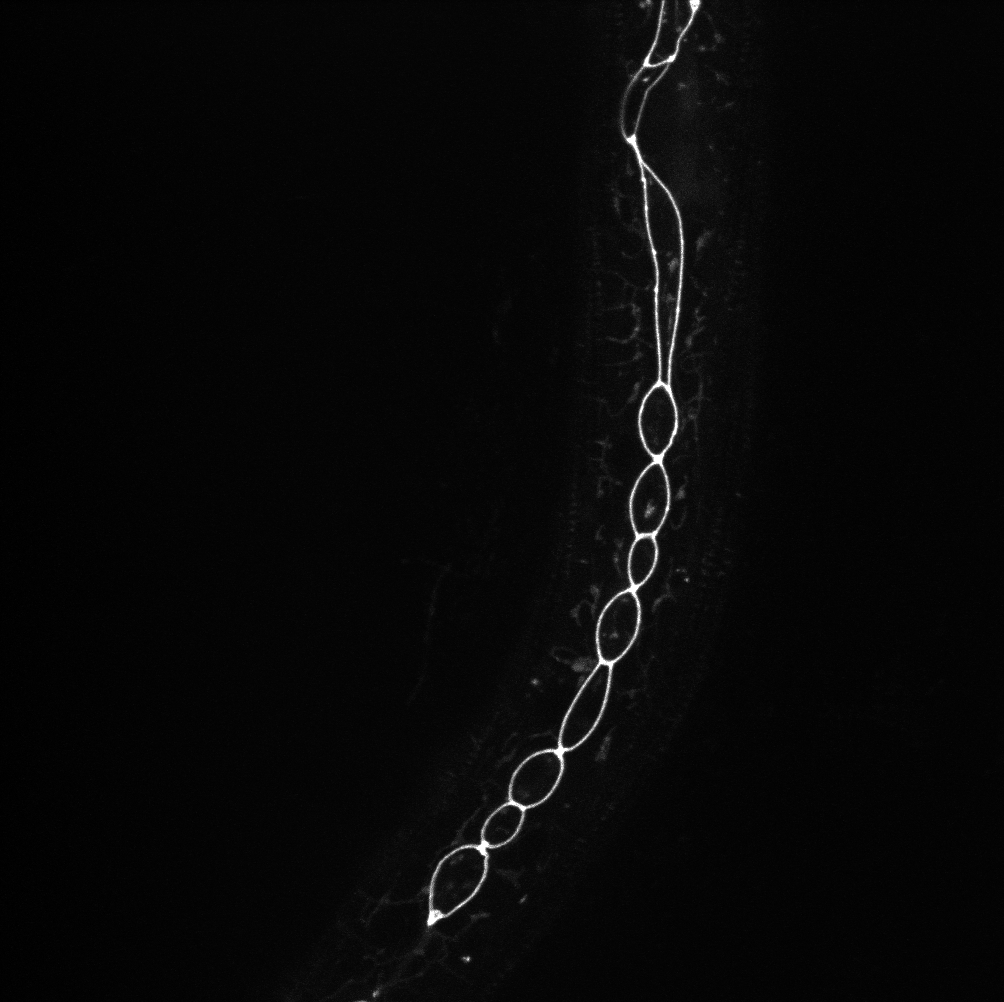

Supplement: Figure 4—source data 1. — This zip archive contains the microscopy images shown in panels A, C, E, and F, and the data graphed in panels B, D. The images are in TIFF and Adobe Photoshop format. The TIFF file is the unadjusted grayscale maximum intensity projection image generated in ImageJ from an image stack. The Photoshop file contains the original image with the adjustment layers used to arrive at the final image displayed in the main figure. The raw graph data are in Microsoft Excel format, and the summary data and actual graph in Graphpad Prism format. [file elife-62067-fig4-data1.zip › Fig 4/F/MAX_300119_P6deg_pwrt2TIR_DLG-1mCh_L2_A15h_31_w2SpinningDisc - Red-4-to-6.tif]

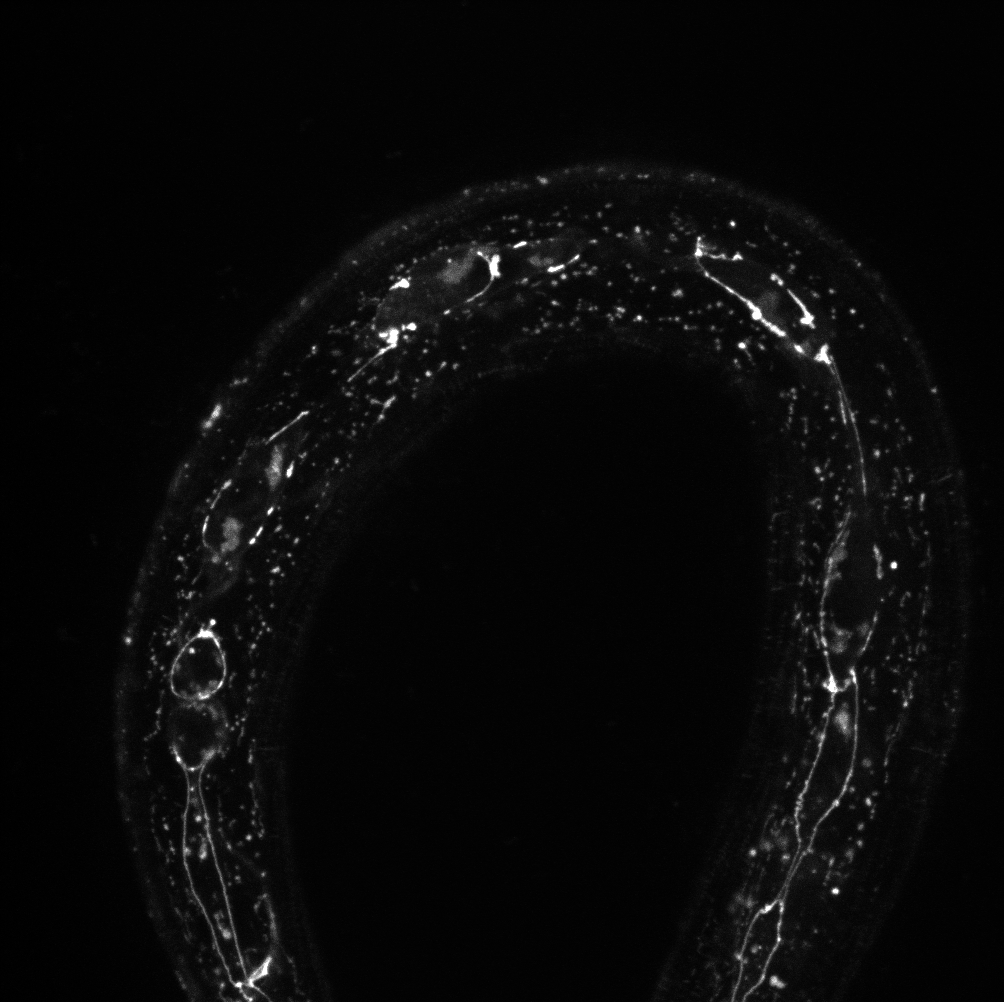

Supplement: Figure 4—source data 1. — This zip archive contains the microscopy images shown in panels A, C, E, and F, and the data graphed in panels B, D. The images are in TIFF and Adobe Photoshop format. The TIFF file is the unadjusted grayscale maximum intensity projection image generated in ImageJ from an image stack. The Photoshop file contains the original image with the adjustment layers used to arrive at the final image displayed in the main figure. The raw graph data are in Microsoft Excel format, and the summary data and actual graph in Graphpad Prism format. [file elife-62067-fig4-data1.zip › Fig 4/F/MAX_300119_P6deg_pwrt2TIR_DLG-1mCh_L2_A21h_10_w2SpinningDisc - Red-7-to-9.tif]

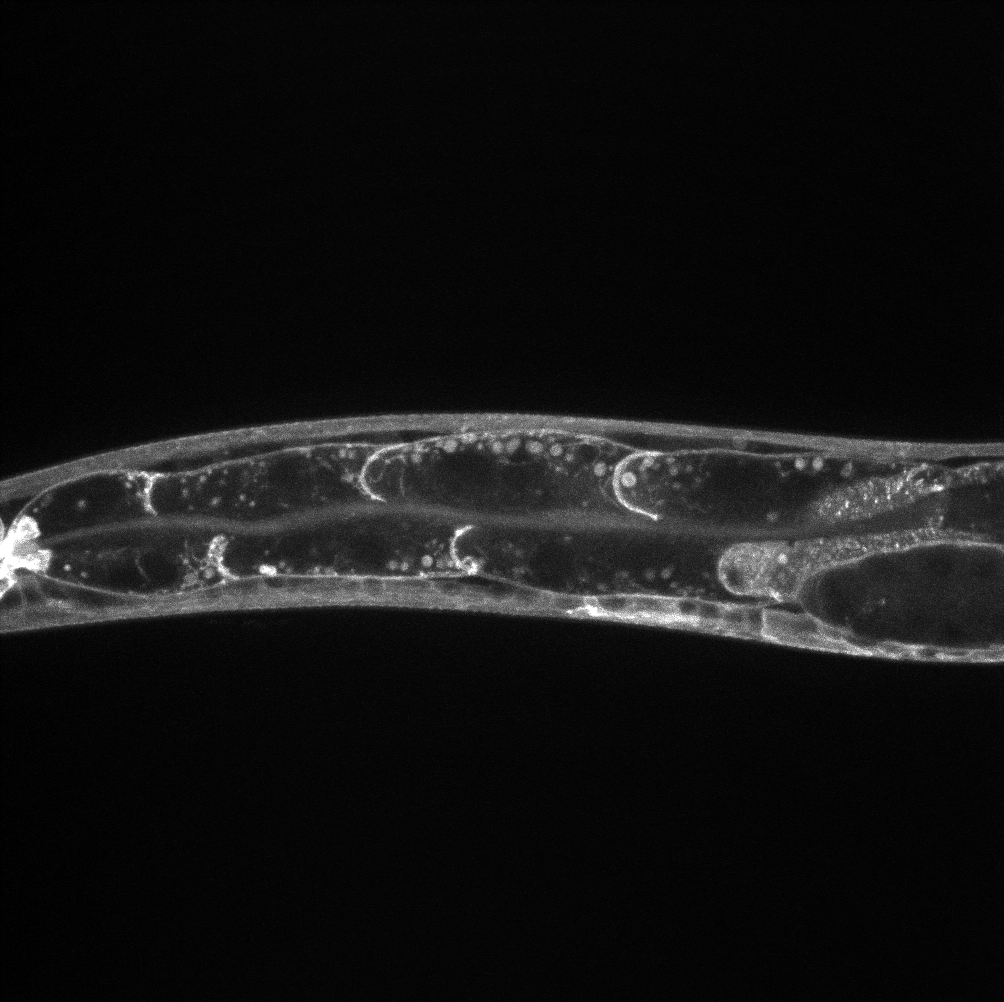

Supplement: Figure 4—figure supplement 1—source data 1. — This zip archive contains the microscopy images shown in panels A and B. The images are in TIFF and Adobe Photoshop format. The TIFF file is the unadjusted grayscale maximum intensity projection image generated in ImageJ from an image stack. The Photoshop file contains the original image with the adjustment layers used to arrive at the final image displayed in the main figure. [file elife-62067-fig4-figsupp1-data1.zip › Fig 4S1/A/MAX_190827_LGLdgpXPKC3deg_aux_26_w1SpinningDisc - Green-22-to-26.tif]

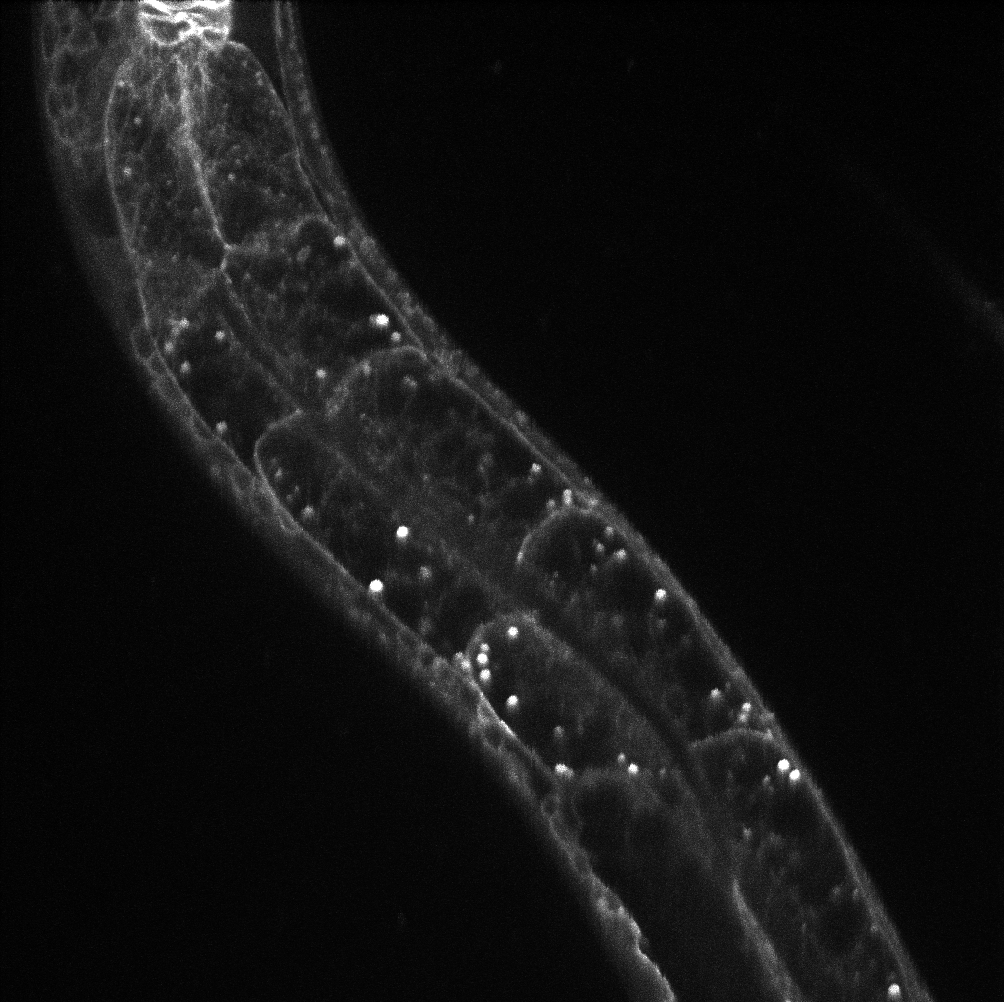

Supplement: Figure 4—figure supplement 1—source data 1. — This zip archive contains the microscopy images shown in panels A and B. The images are in TIFF and Adobe Photoshop format. The TIFF file is the unadjusted grayscale maximum intensity projection image generated in ImageJ from an image stack. The Photoshop file contains the original image with the adjustment layers used to arrive at the final image displayed in the main figure. [file elife-62067-fig4-figsupp1-data1.zip › Fig 4S1/A/MAX_200707_lgl1gfp2.tif]

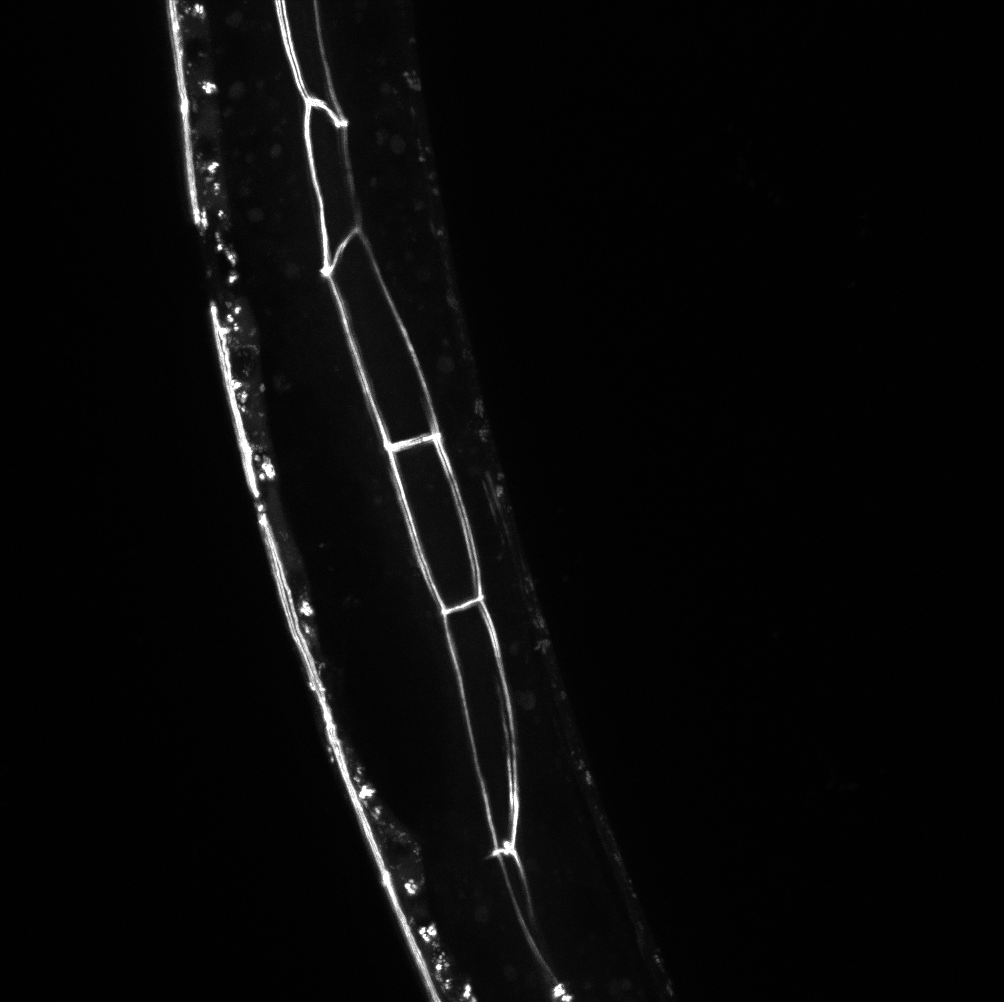

Supplement: Figure 4—figure supplement 1—source data 1. — This zip archive contains the microscopy images shown in panels A and B. The images are in TIFF and Adobe Photoshop format. The TIFF file is the unadjusted grayscale maximum intensity projection image generated in ImageJ from an image stack. The Photoshop file contains the original image with the adjustment layers used to arrive at the final image displayed in the main figure. [file elife-62067-fig4-figsupp1-data1.zip › Fig 4S1/B/MAX_190528_BOX431_Auxin_2_w2SpinningDisc - Red.tif]

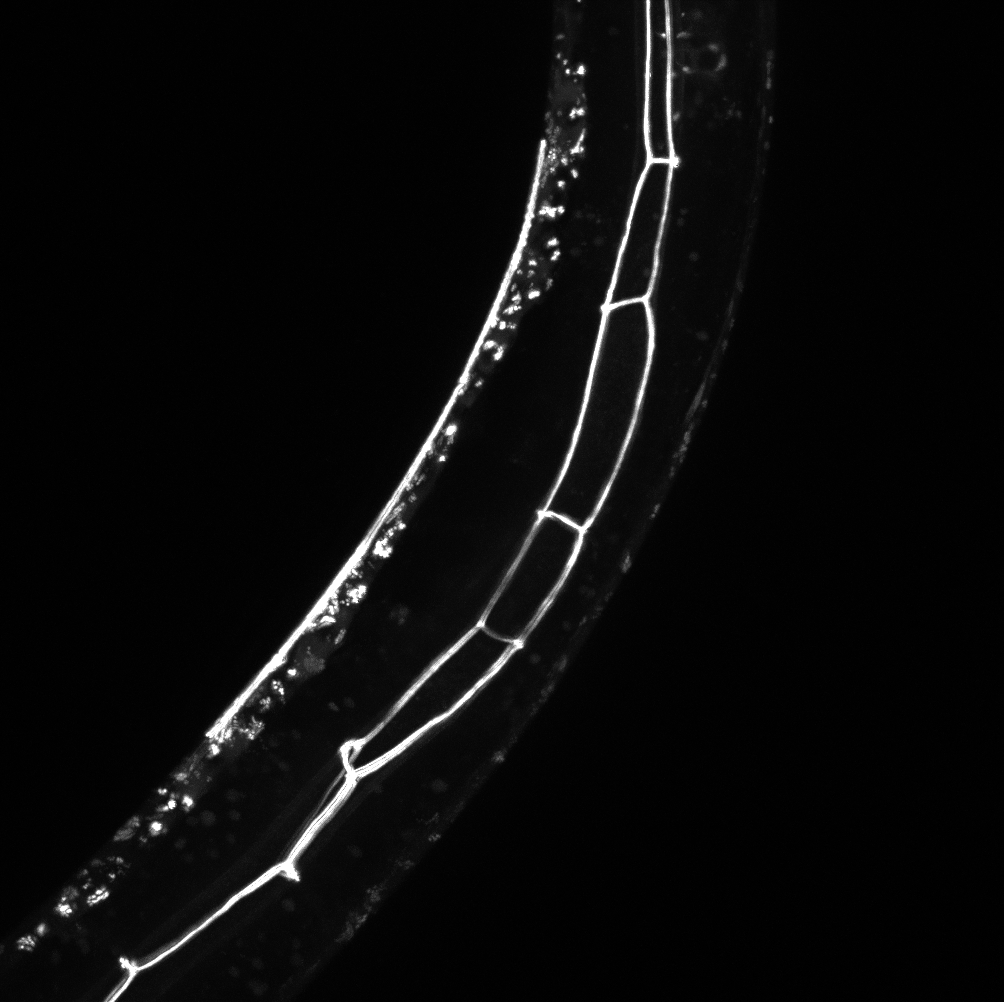

Supplement: Figure 4—figure supplement 1—source data 1. — This zip archive contains the microscopy images shown in panels A and B. The images are in TIFF and Adobe Photoshop format. The TIFF file is the unadjusted grayscale maximum intensity projection image generated in ImageJ from an image stack. The Photoshop file contains the original image with the adjustment layers used to arrive at the final image displayed in the main figure. [file elife-62067-fig4-figsupp1-data1.zip › Fig 4S1/B/MAX_190528_BOX431_Control9_w2SpinningDisc - Red.tif]

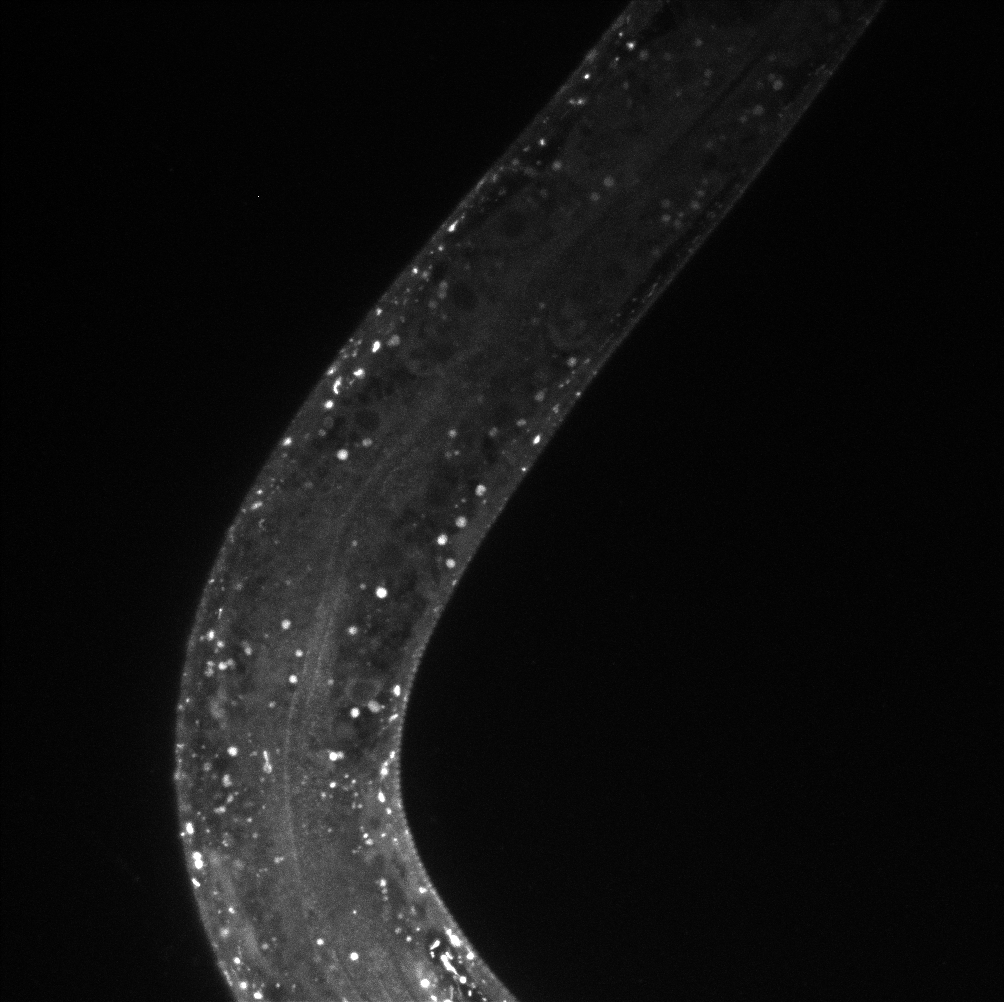

Supplement: Figure 4—figure supplement 2—source data 1. — This zip archive contains the microscopy images and the data graphed in panels A–D. The images are in TIFF and Adobe Photoshop format. The TIFF file is the unadjusted grayscale maximum intensity projection image generated in ImageJ from an image stack. The Photoshop file contains the original image with the adjustment layers used to arrive at the final image displayed in the main figure. The raw graph data are in Microsoft Excel format, and the summary data and actual graph in Graphpad Prism format. [file elife-62067-fig4-figsupp2-data1.zip › Fig 4S2/A/MAX_190117_PKC-3_Par-6_elt2_AUXIN_6_w2SpinningDisc - Red.tif]

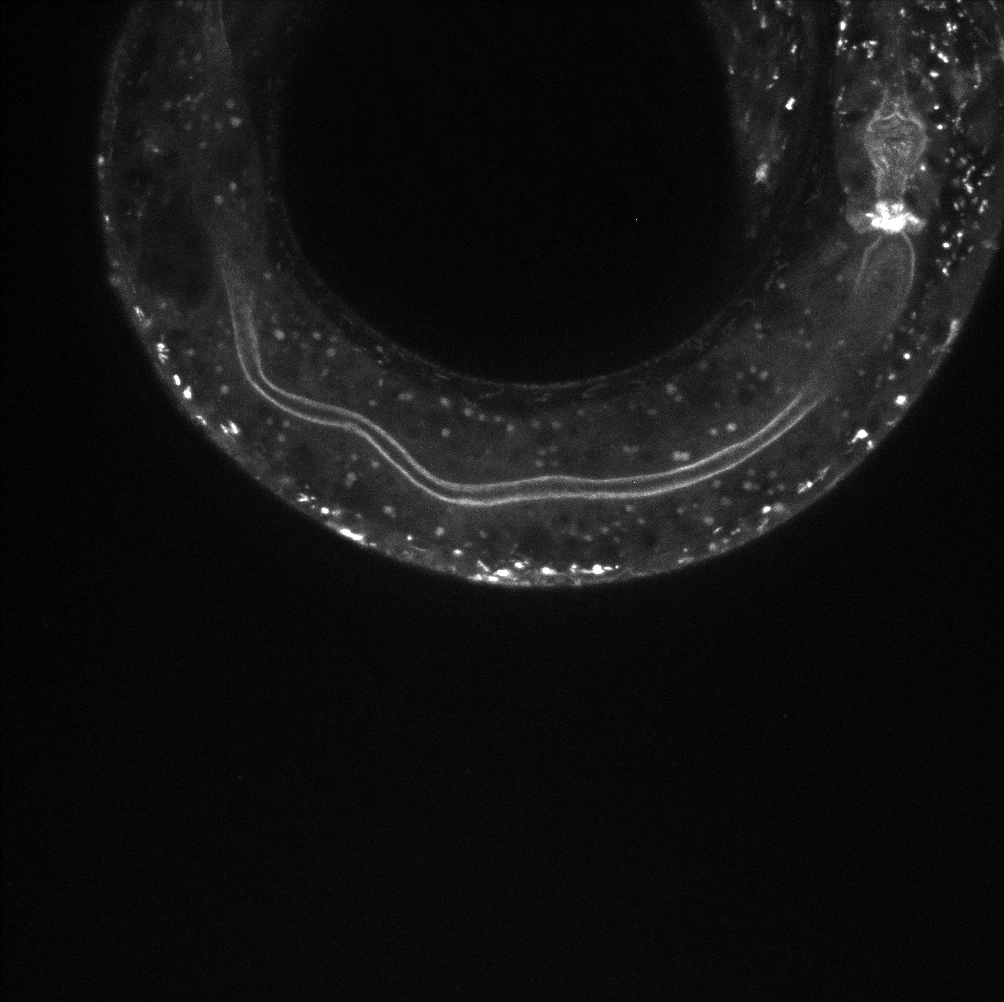

Supplement: Figure 4—figure supplement 2—source data 1. — This zip archive contains the microscopy images and the data graphed in panels A–D. The images are in TIFF and Adobe Photoshop format. The TIFF file is the unadjusted grayscale maximum intensity projection image generated in ImageJ from an image stack. The Photoshop file contains the original image with the adjustment layers used to arrive at the final image displayed in the main figure. The raw graph data are in Microsoft Excel format, and the summary data and actual graph in Graphpad Prism format. [file elife-62067-fig4-figsupp2-data1.zip › Fig 4S2/A/MAX_190117_PKC-3_Par-6_elt2_Control_1_w2SpinningDisc - Red-10-to-15.tif]

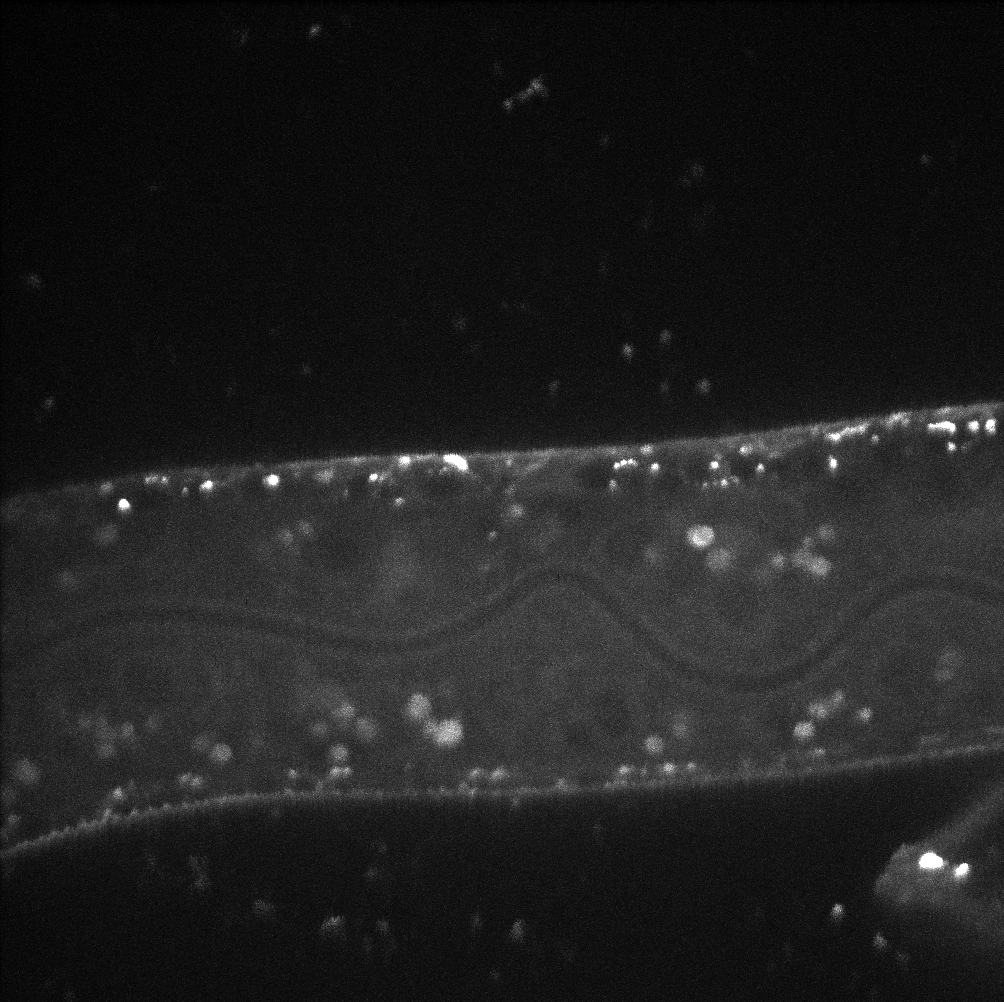

Supplement: Figure 4—figure supplement 2—source data 1. — This zip archive contains the microscopy images and the data graphed in panels A–D. The images are in TIFF and Adobe Photoshop format. The TIFF file is the unadjusted grayscale maximum intensity projection image generated in ImageJ from an image stack. The Photoshop file contains the original image with the adjustment layers used to arrive at the final image displayed in the main figure. The raw graph data are in Microsoft Excel format, and the summary data and actual graph in Graphpad Prism format. [file elife-62067-fig4-figsupp2-data1.zip › Fig 4S2/B/MAX_201027_PKC3aid_P6mch_pelt2_A_2h_8_w1SpinningDisc - Red-4-to-9.tif]

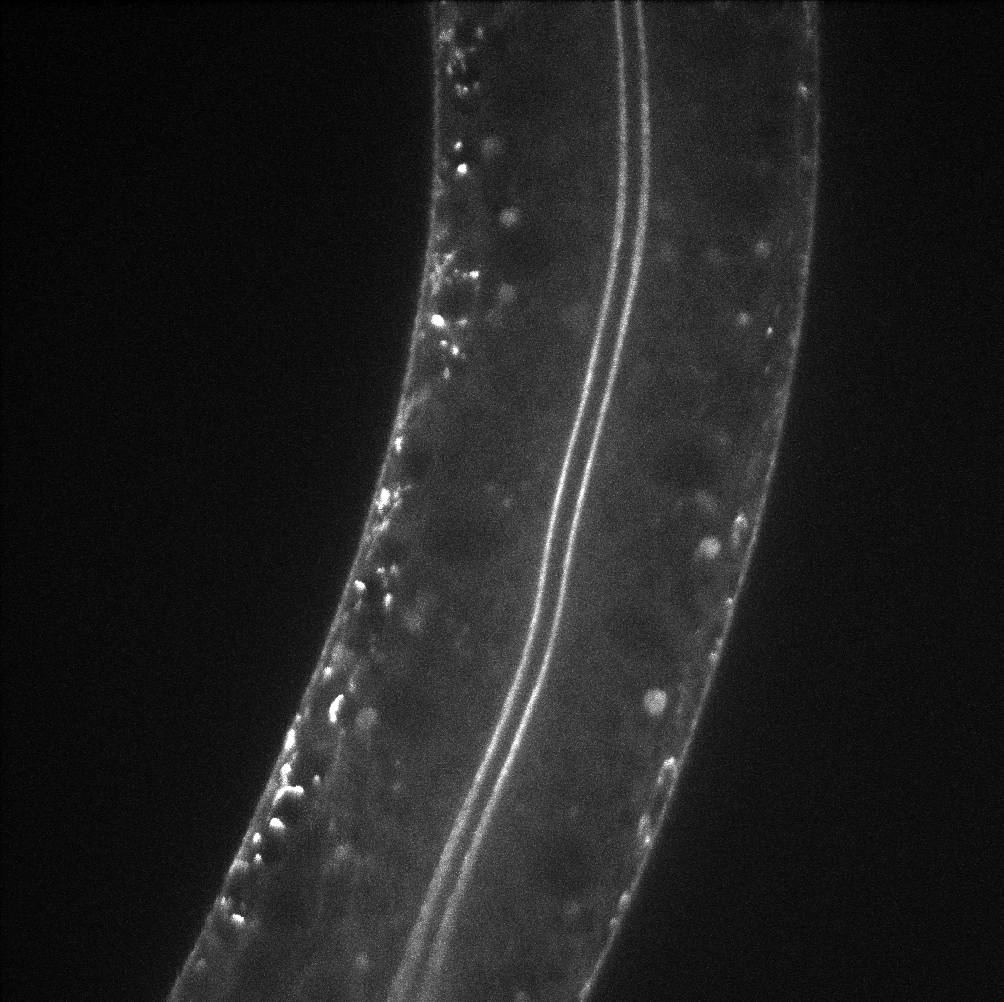

Supplement: Figure 4—figure supplement 2—source data 1. — This zip archive contains the microscopy images and the data graphed in panels A–D. The images are in TIFF and Adobe Photoshop format. The TIFF file is the unadjusted grayscale maximum intensity projection image generated in ImageJ from an image stack. The Photoshop file contains the original image with the adjustment layers used to arrive at the final image displayed in the main figure. The raw graph data are in Microsoft Excel format, and the summary data and actual graph in Graphpad Prism format. [file elife-62067-fig4-figsupp2-data1.zip › Fig 4S2/B/MAX_201027_PKC3aid_P6mch_pelt2_C_2_w1SpinningDisc - Red-1-to-6.tif]

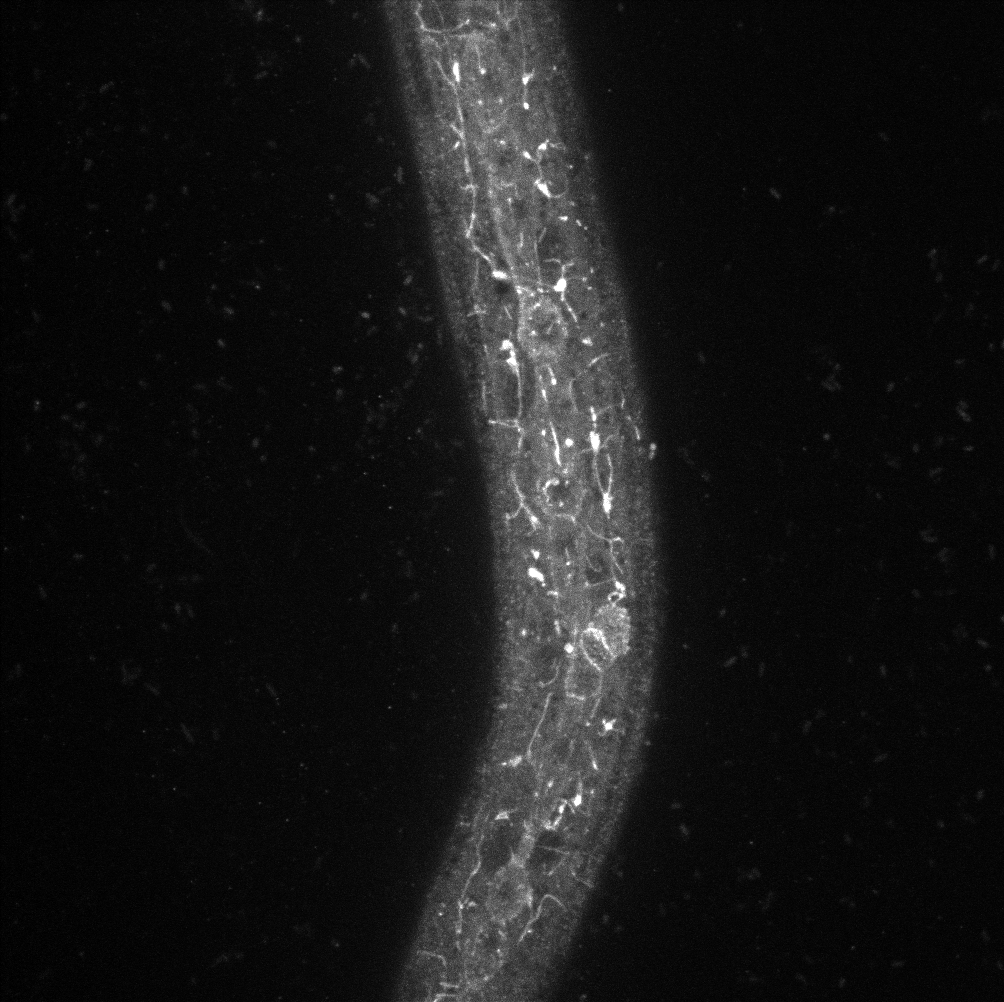

Supplement: Figure 4—figure supplement 2—source data 1. — This zip archive contains the microscopy images and the data graphed in panels A–D. The images are in TIFF and Adobe Photoshop format. The TIFF file is the unadjusted grayscale maximum intensity projection image generated in ImageJ from an image stack. The Photoshop file contains the original image with the adjustment layers used to arrive at the final image displayed in the main figure. The raw graph data are in Microsoft Excel format, and the summary data and actual graph in Graphpad Prism format. [file elife-62067-fig4-figsupp2-data1.zip › Fig 4S2/C/MAX_220119_P6deg_pwrt2TIR_mChPKC3_C_15_w2SpinningDisc - Red-13-to-15.tif]

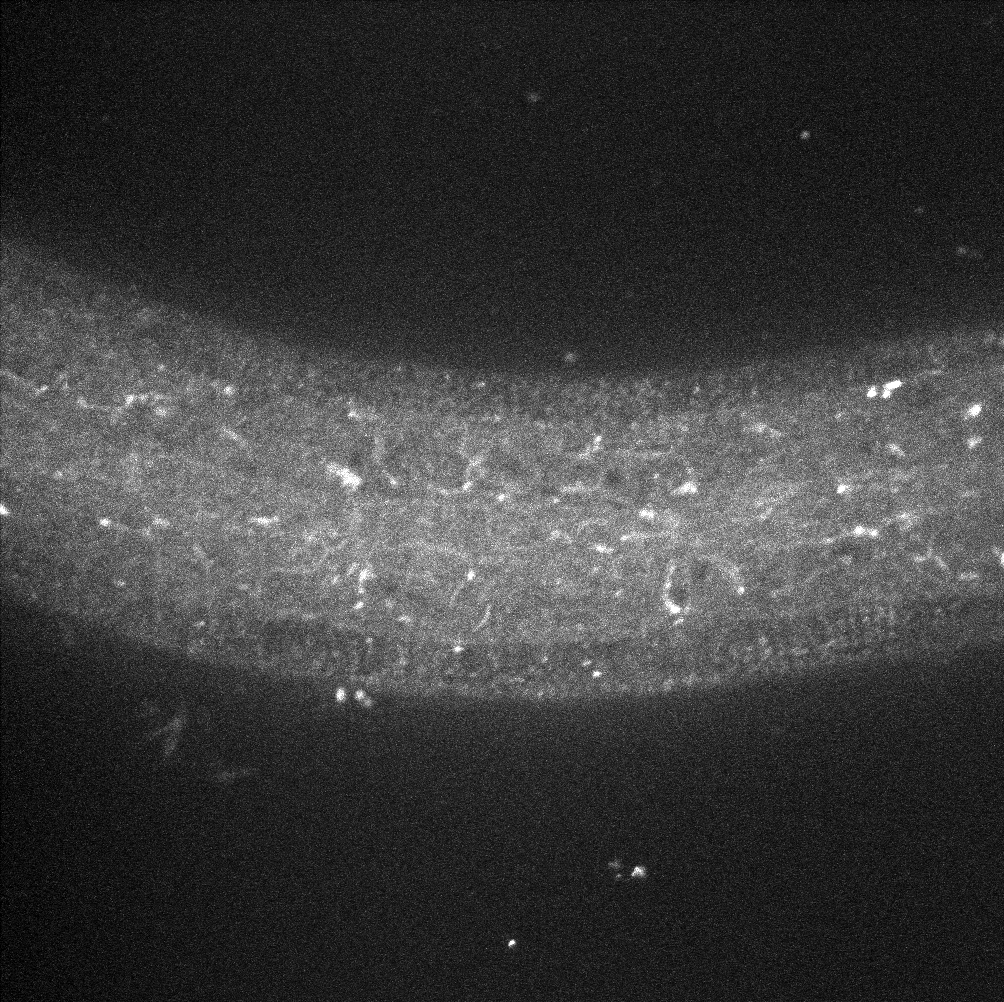

Supplement: Figure 4—figure supplement 2—source data 1. — This zip archive contains the microscopy images and the data graphed in panels A–D. The images are in TIFF and Adobe Photoshop format. The TIFF file is the unadjusted grayscale maximum intensity projection image generated in ImageJ from an image stack. The Photoshop file contains the original image with the adjustment layers used to arrive at the final image displayed in the main figure. The raw graph data are in Microsoft Excel format, and the summary data and actual graph in Graphpad Prism format. [file elife-62067-fig4-figsupp2-data1.zip › Fig 4S2/D/MAX_191030_PKC3AID_P6mCh_L3_A_8_w2SpinningDisc - Red-10-to-12.tif]

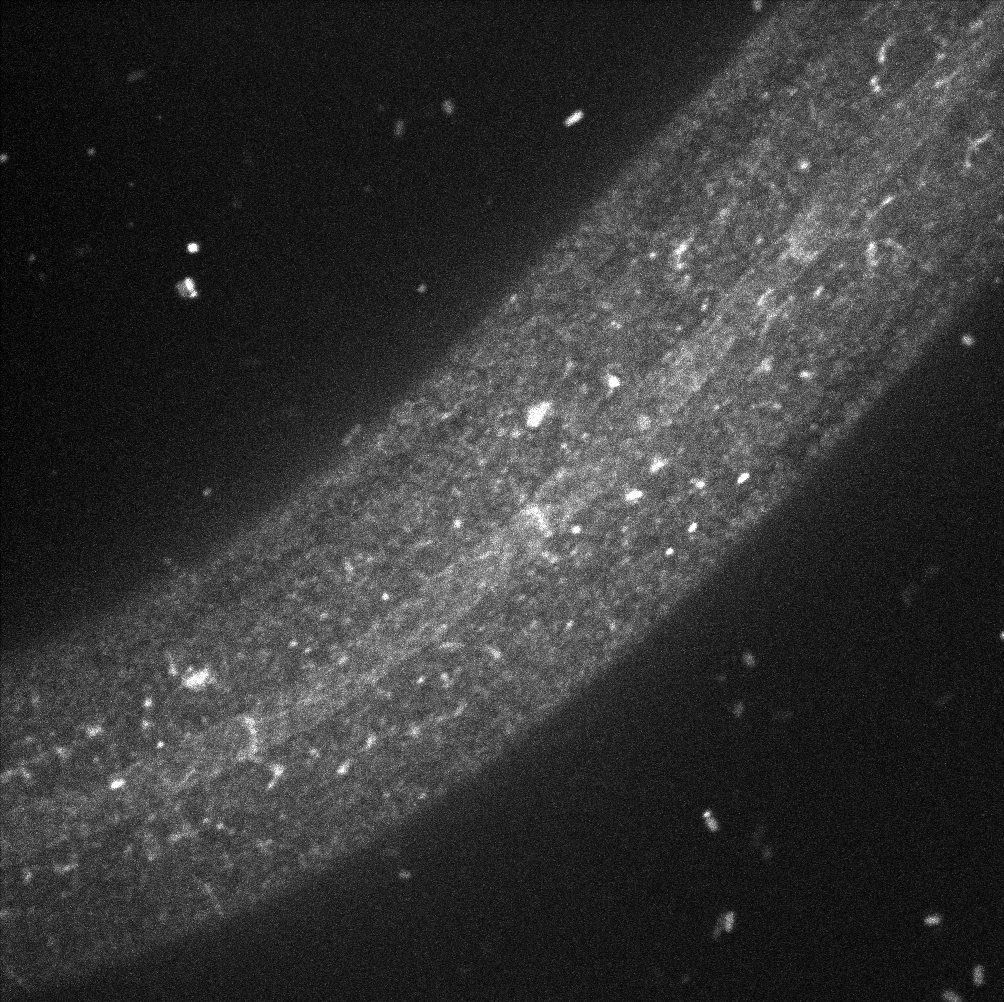

Supplement: Figure 4—figure supplement 2—source data 1. — This zip archive contains the microscopy images and the data graphed in panels A–D. The images are in TIFF and Adobe Photoshop format. The TIFF file is the unadjusted grayscale maximum intensity projection image generated in ImageJ from an image stack. The Photoshop file contains the original image with the adjustment layers used to arrive at the final image displayed in the main figure. The raw graph data are in Microsoft Excel format, and the summary data and actual graph in Graphpad Prism format. [file elife-62067-fig4-figsupp2-data1.zip › Fig 4S2/D/MAX_191030_PKC3AID_P6mCh_L3_C_8_w2SpinningDisc - Red-6-to-8.tif]

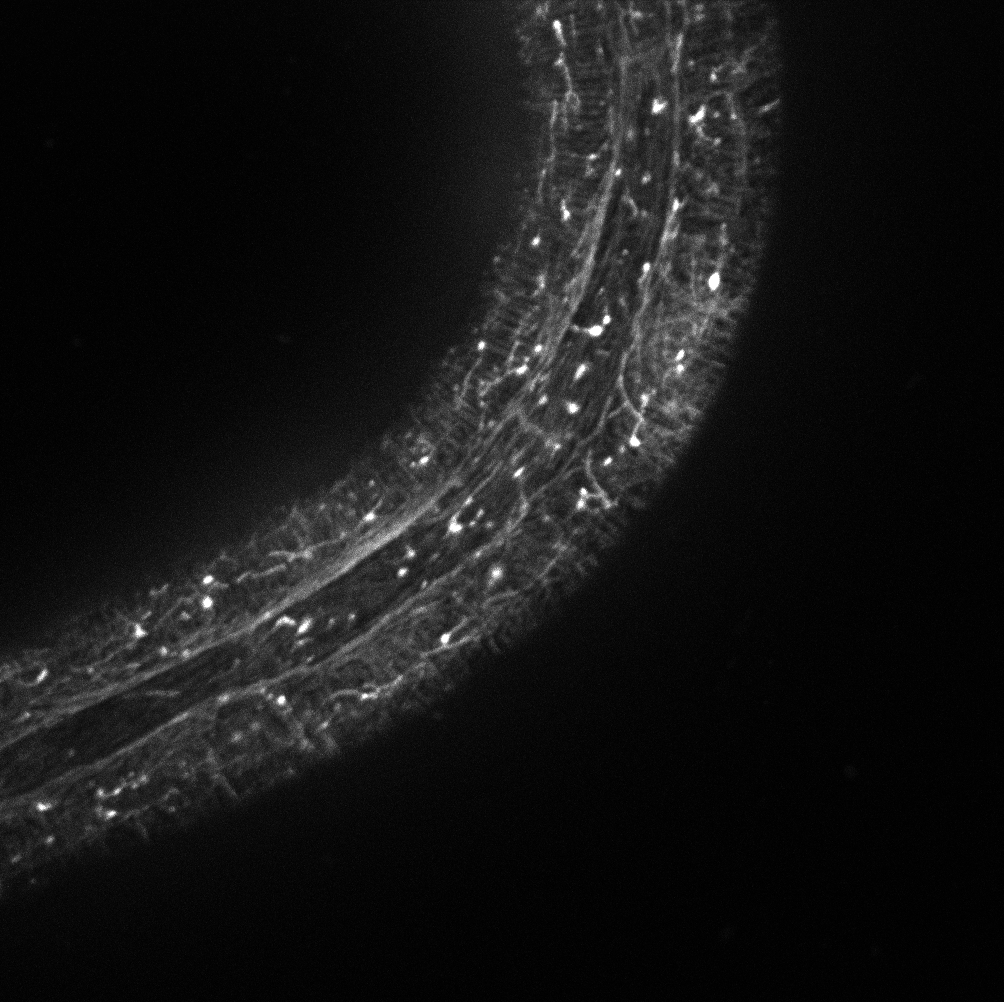

Supplement: Figure 5—source data 1. — This zip archive contains the microscopy images shown in panels A, C, and E, and the data graphed in panels B, D, F, G, and H. The images are in TIFF and Adobe Photoshop format. The TIFF file is the unadjusted grayscale maximum intensity projection image generated in ImageJ from an image stack. The Photoshop file contains the original image with the adjustment layers used to arrive at the final image displayed in the main figure. The raw graph data are in Microsoft Excel format, and the summary data and actual graph in Graphpad Prism format. [file elife-62067-fig5-data1.zip › Fig 5/A/MAX_190605_P6deg_actinSV_A_3_w2SpinningDisc - Red-11-to-13.tif]

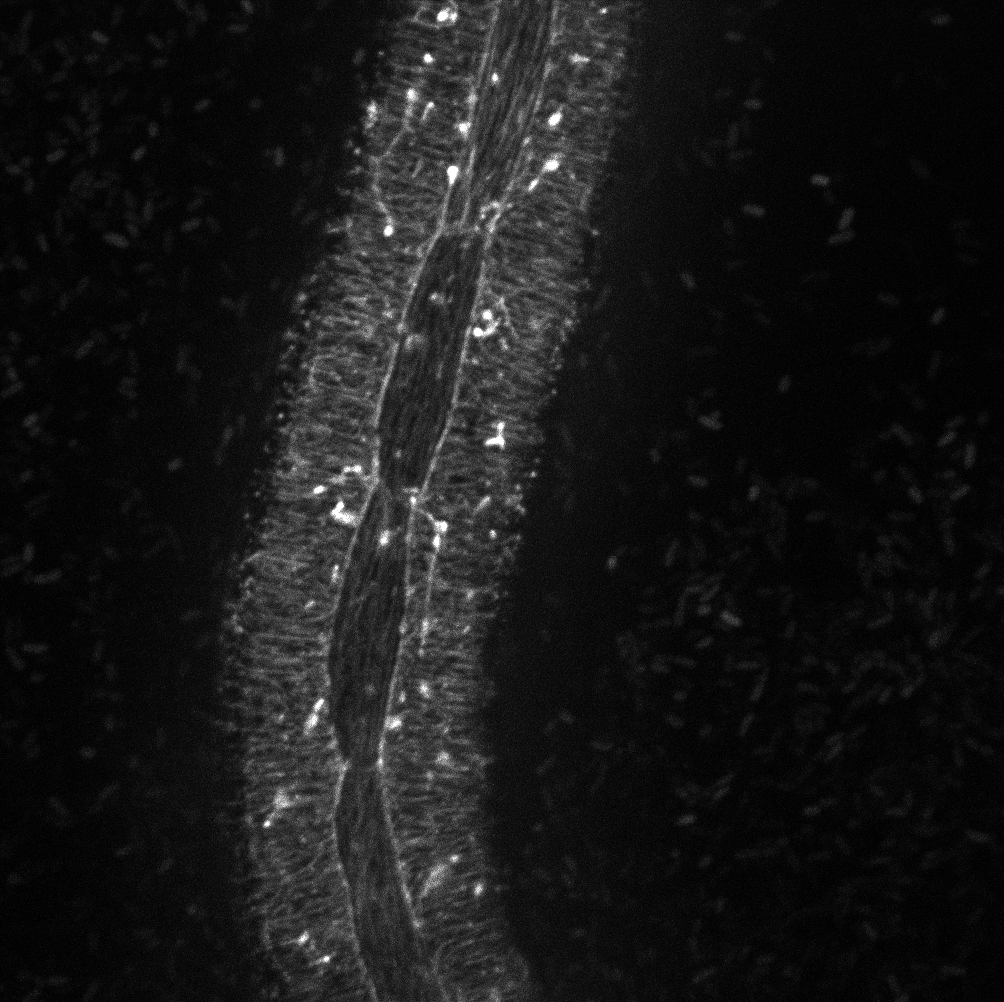

Supplement: Figure 5—source data 1. — This zip archive contains the microscopy images shown in panels A, C, and E, and the data graphed in panels B, D, F, G, and H. The images are in TIFF and Adobe Photoshop format. The TIFF file is the unadjusted grayscale maximum intensity projection image generated in ImageJ from an image stack. The Photoshop file contains the original image with the adjustment layers used to arrive at the final image displayed in the main figure. The raw graph data are in Microsoft Excel format, and the summary data and actual graph in Graphpad Prism format. [file elife-62067-fig5-data1.zip › Fig 5/A/MAX_190605_P6deg_actinSV_L3_C_7_w2SpinningDisc - Red-12-to-14.tif]

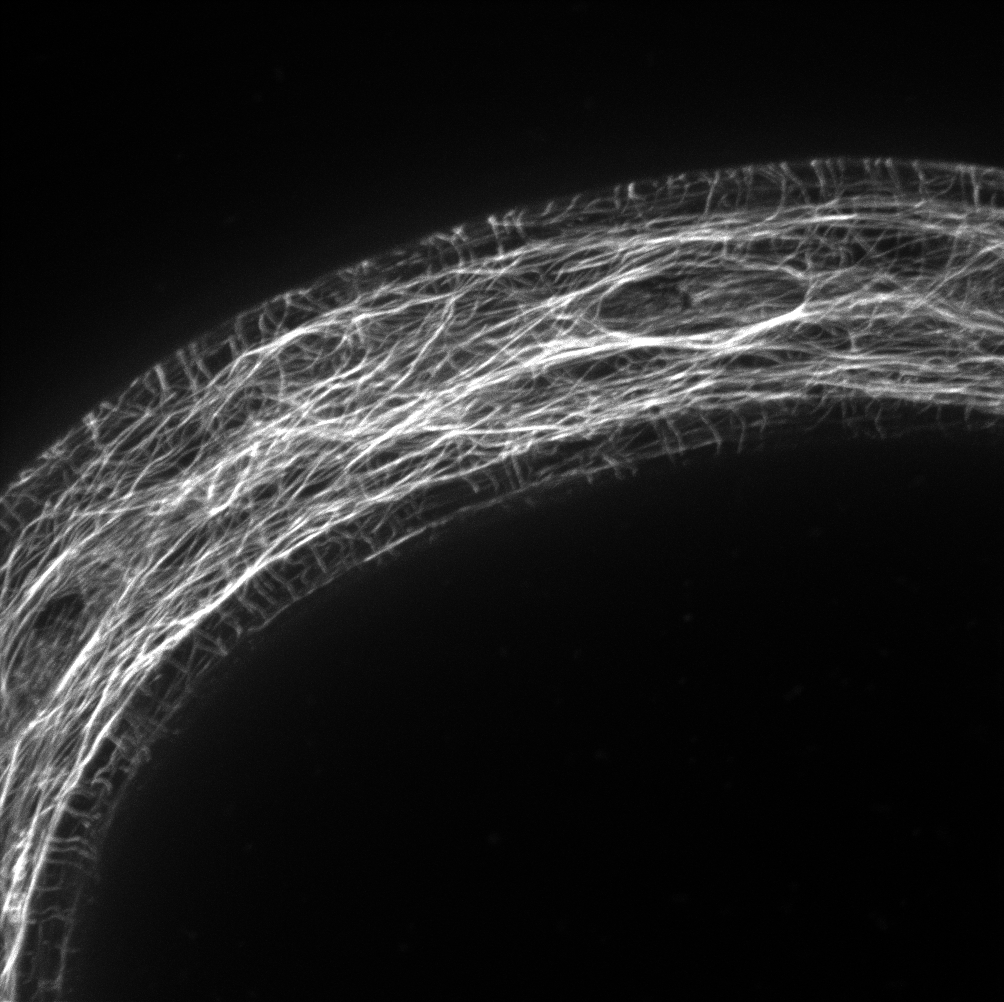

Supplement: Figure 5—source data 1. — This zip archive contains the microscopy images shown in panels A, C, and E, and the data graphed in panels B, D, F, G, and H. The images are in TIFF and Adobe Photoshop format. The TIFF file is the unadjusted grayscale maximum intensity projection image generated in ImageJ from an image stack. The Photoshop file contains the original image with the adjustment layers used to arrive at the final image displayed in the main figure. The raw graph data are in Microsoft Excel format, and the summary data and actual graph in Graphpad Prism format. [file elife-62067-fig5-data1.zip › Fig 5/C/MAX_190605_P6deg_maph1_L3_A_10_w1SpinningDisc - Green-3-to-11.tif]

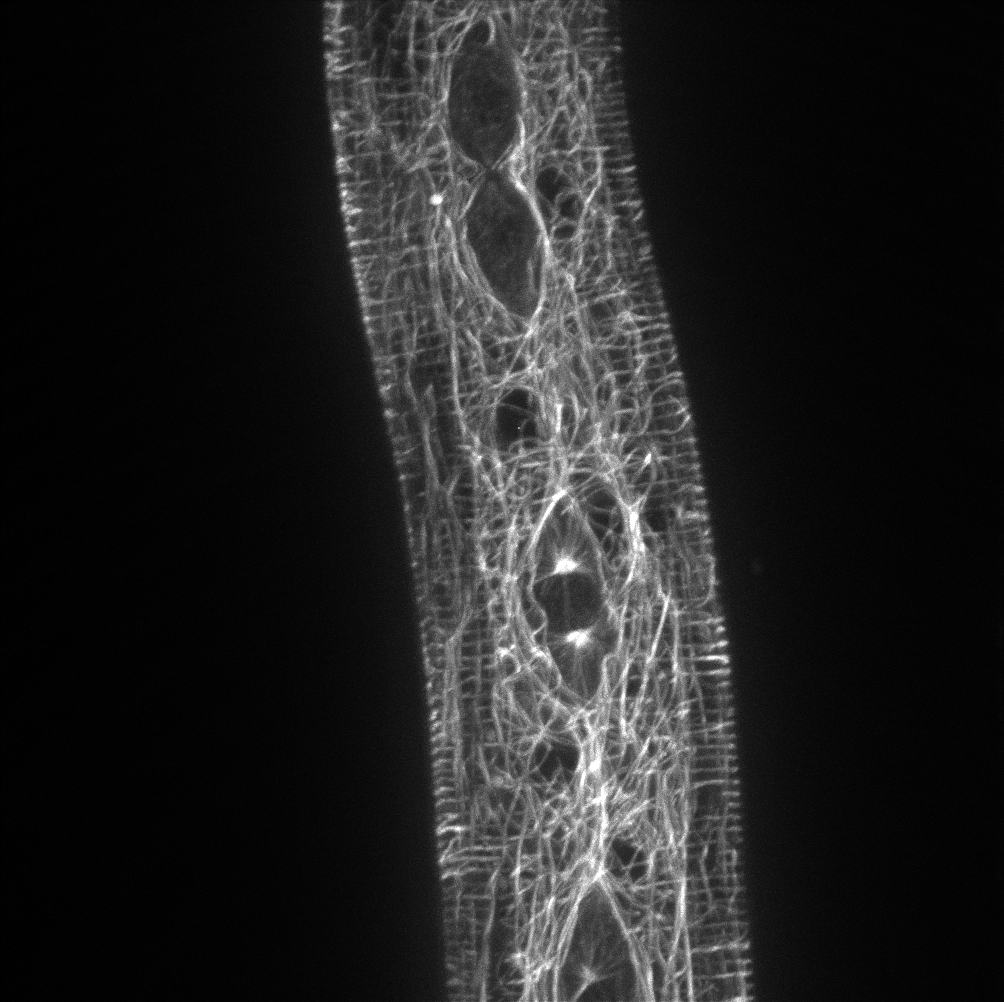

Supplement: Figure 5—source data 1. — This zip archive contains the microscopy images shown in panels A, C, and E, and the data graphed in panels B, D, F, G, and H. The images are in TIFF and Adobe Photoshop format. The TIFF file is the unadjusted grayscale maximum intensity projection image generated in ImageJ from an image stack. The Photoshop file contains the original image with the adjustment layers used to arrive at the final image displayed in the main figure. The raw graph data are in Microsoft Excel format, and the summary data and actual graph in Graphpad Prism format. [file elife-62067-fig5-data1.zip › Fig 5/C/MAX_190605_P6deg_maph1_L3_C_3_w1SpinningDisc - Green-1-to-10.tif]

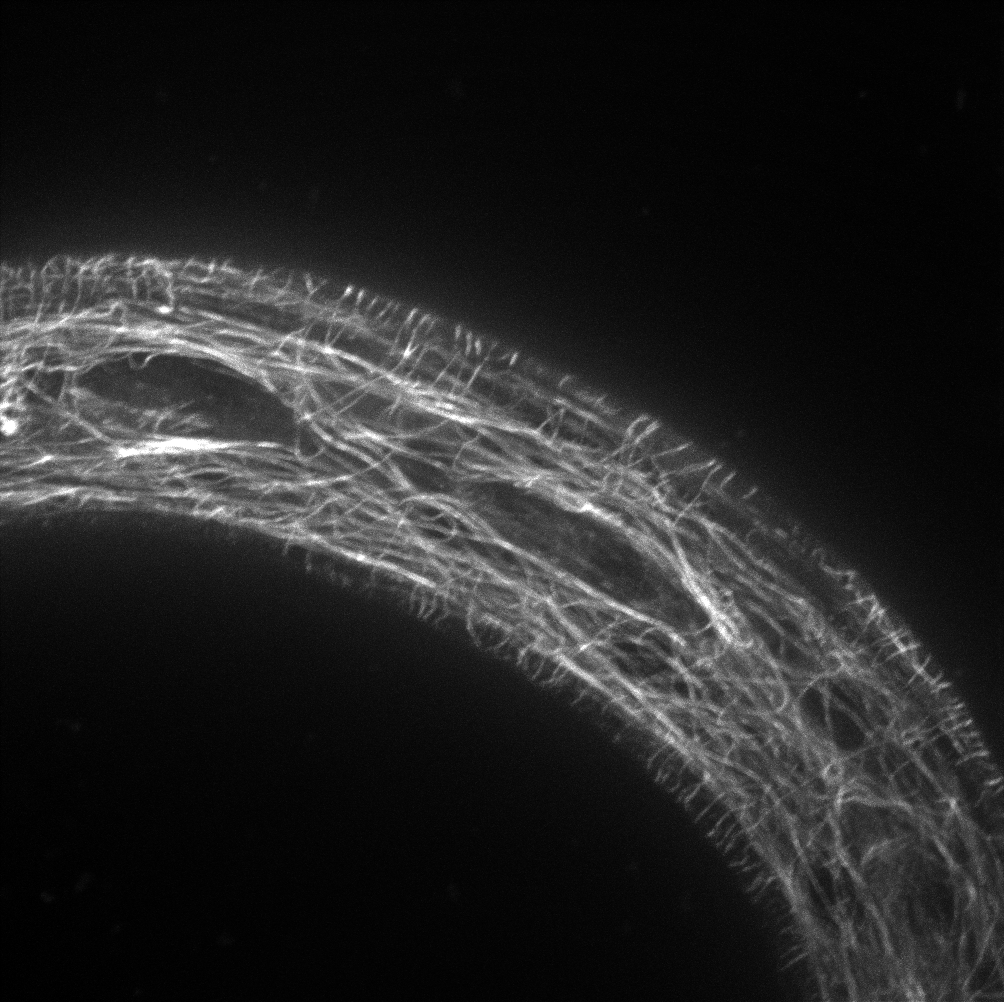

Supplement: Figure 5—source data 1. — This zip archive contains the microscopy images shown in panels A, C, and E, and the data graphed in panels B, D, F, G, and H. The images are in TIFF and Adobe Photoshop format. The TIFF file is the unadjusted grayscale maximum intensity projection image generated in ImageJ from an image stack. The Photoshop file contains the original image with the adjustment layers used to arrive at the final image displayed in the main figure. The raw graph data are in Microsoft Excel format, and the summary data and actual graph in Graphpad Prism format. [file elife-62067-fig5-data1.zip › Fig 5/C/MAX_190905_PKC3AID_maph1_A_15_w1SpinningDisc - Green-4-to-10.tif]

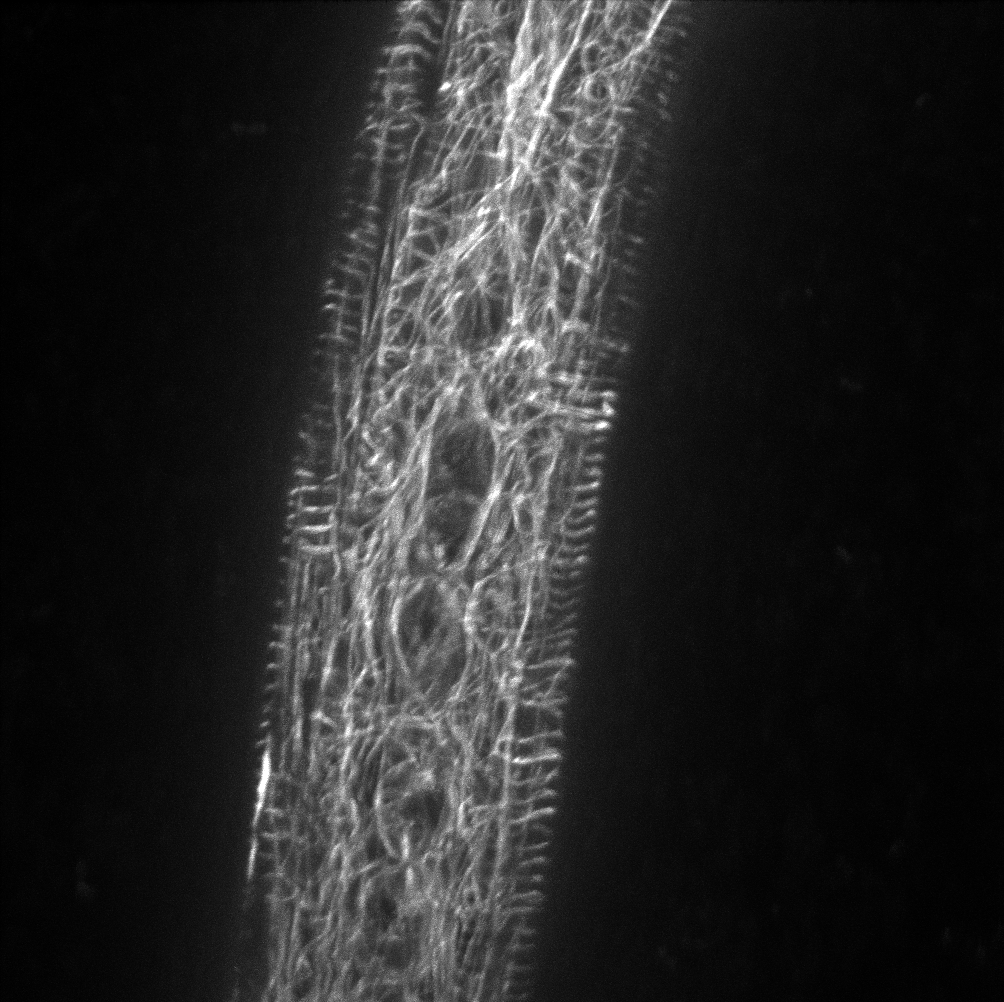

Supplement: Figure 5—source data 1. — This zip archive contains the microscopy images shown in panels A, C, and E, and the data graphed in panels B, D, F, G, and H. The images are in TIFF and Adobe Photoshop format. The TIFF file is the unadjusted grayscale maximum intensity projection image generated in ImageJ from an image stack. The Photoshop file contains the original image with the adjustment layers used to arrive at the final image displayed in the main figure. The raw graph data are in Microsoft Excel format, and the summary data and actual graph in Graphpad Prism format. [file elife-62067-fig5-data1.zip › Fig 5/C/MAX_200326_nocako_maph1_4-6-to-13.tif]

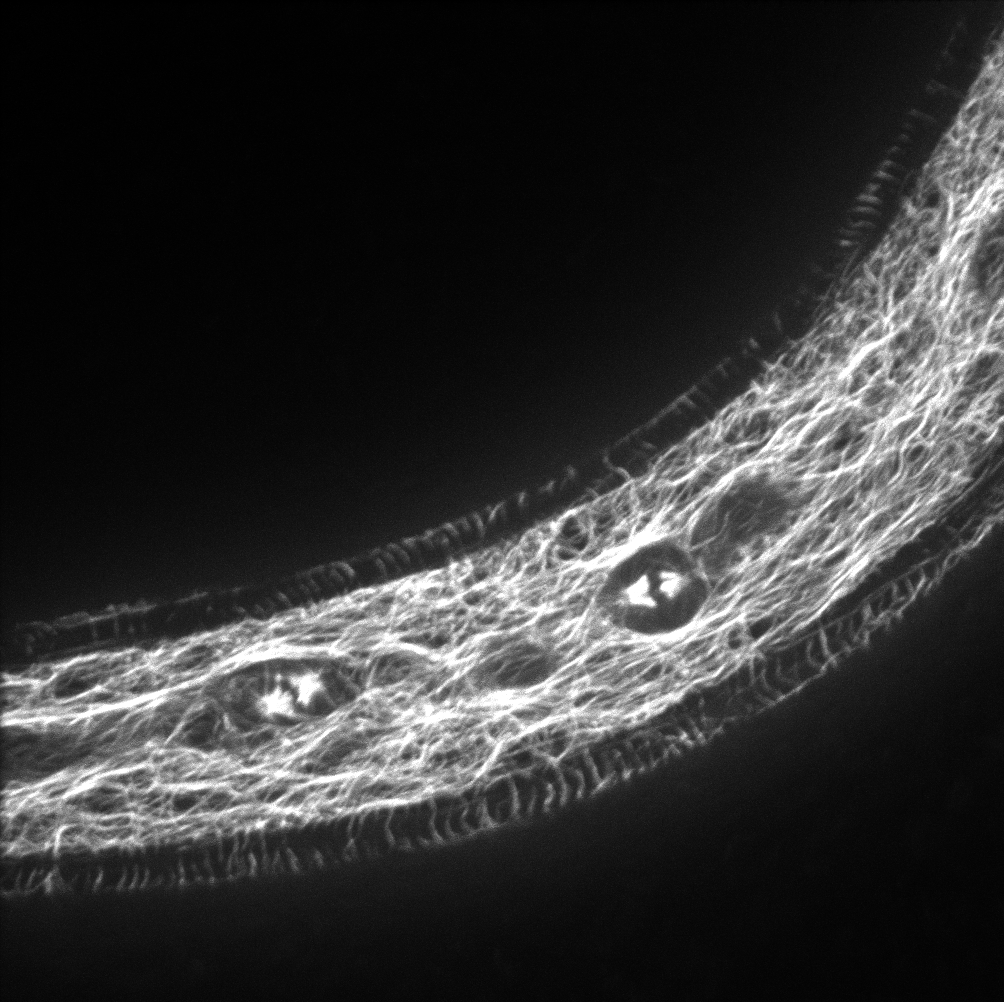

Supplement: Figure 5—source data 1. — This zip archive contains the microscopy images shown in panels A, C, and E, and the data graphed in panels B, D, F, G, and H. The images are in TIFF and Adobe Photoshop format. The TIFF file is the unadjusted grayscale maximum intensity projection image generated in ImageJ from an image stack. The Photoshop file contains the original image with the adjustment layers used to arrive at the final image displayed in the main figure. The raw graph data are in Microsoft Excel format, and the summary data and actual graph in Graphpad Prism format. [file elife-62067-fig5-data1.zip › Fig 5/C/MAX_201103_P6aid_maph1_A_nocako_8-4-to-10.tif]

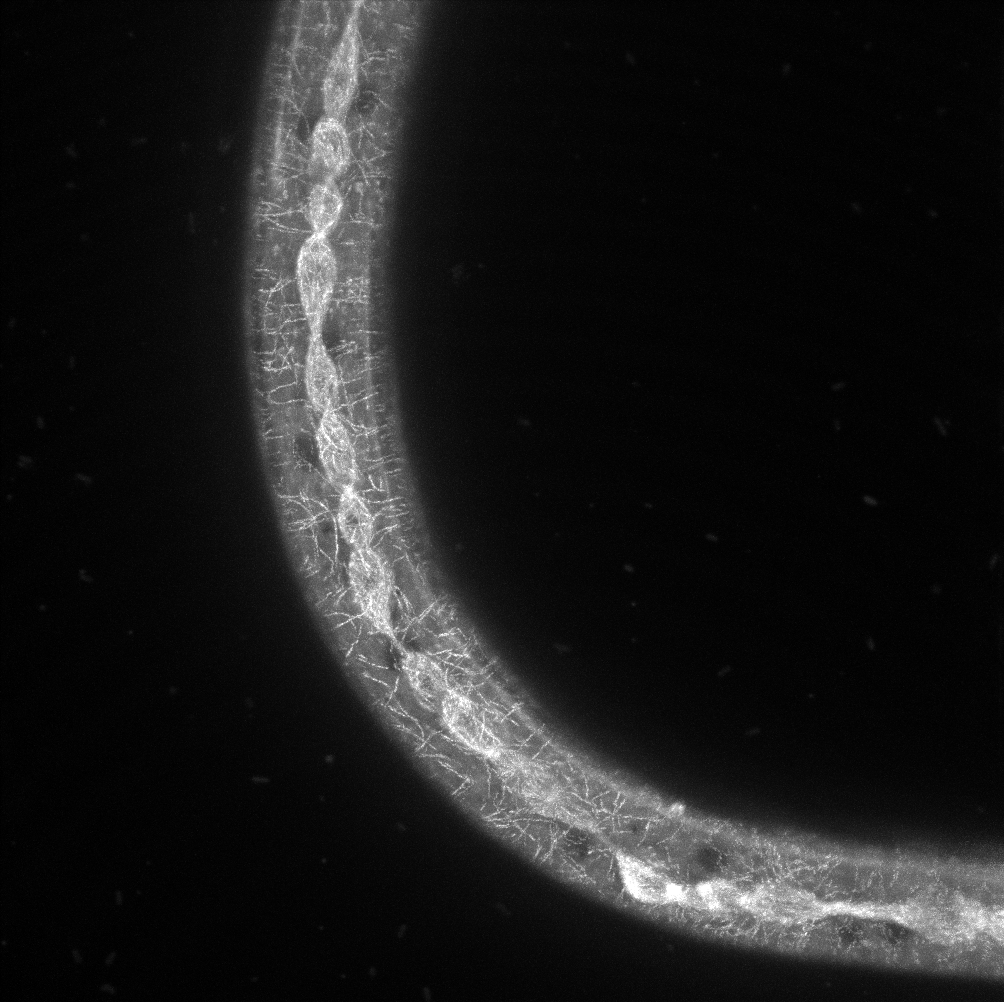

Supplement: Figure 5—source data 1. — This zip archive contains the microscopy images shown in panels A, C, and E, and the data graphed in panels B, D, F, G, and H. The images are in TIFF and Adobe Photoshop format. The TIFF file is the unadjusted grayscale maximum intensity projection image generated in ImageJ from an image stack. The Photoshop file contains the original image with the adjustment layers used to arrive at the final image displayed in the main figure. The raw graph data are in Microsoft Excel format, and the summary data and actual graph in Graphpad Prism format. [file elife-62067-fig5-data1.zip › Fig 5/E/MAX_191002_P6AID_ebp2eGFP_C_L2short_3.tif]

# EB-2 comet density (comets/100 $\mu\text{m}^2$ )

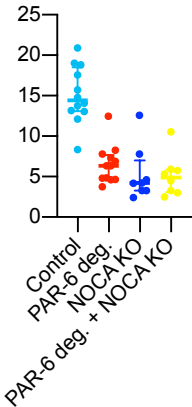

Supplement: Figure 5—source data 1. — This zip archive contains the microscopy images shown in panels A, C, and E, and the data graphed in panels B, D, F, G, and H. The images are in TIFF and Adobe Photoshop format. The TIFF file is the unadjusted grayscale maximum intensity projection image generated in ImageJ from an image stack. The Photoshop file contains the original image with the adjustment layers used to arrive at the final image displayed in the main figure. The raw graph data are in Microsoft Excel format, and the summary data and actual graph in Graphpad Prism format. [file elife-62067-fig5-data1.zip › Fig 5/F/191002 - Comet density hyp7 - L2 - 1 to 2 hours - noca1 - combined.pdf]

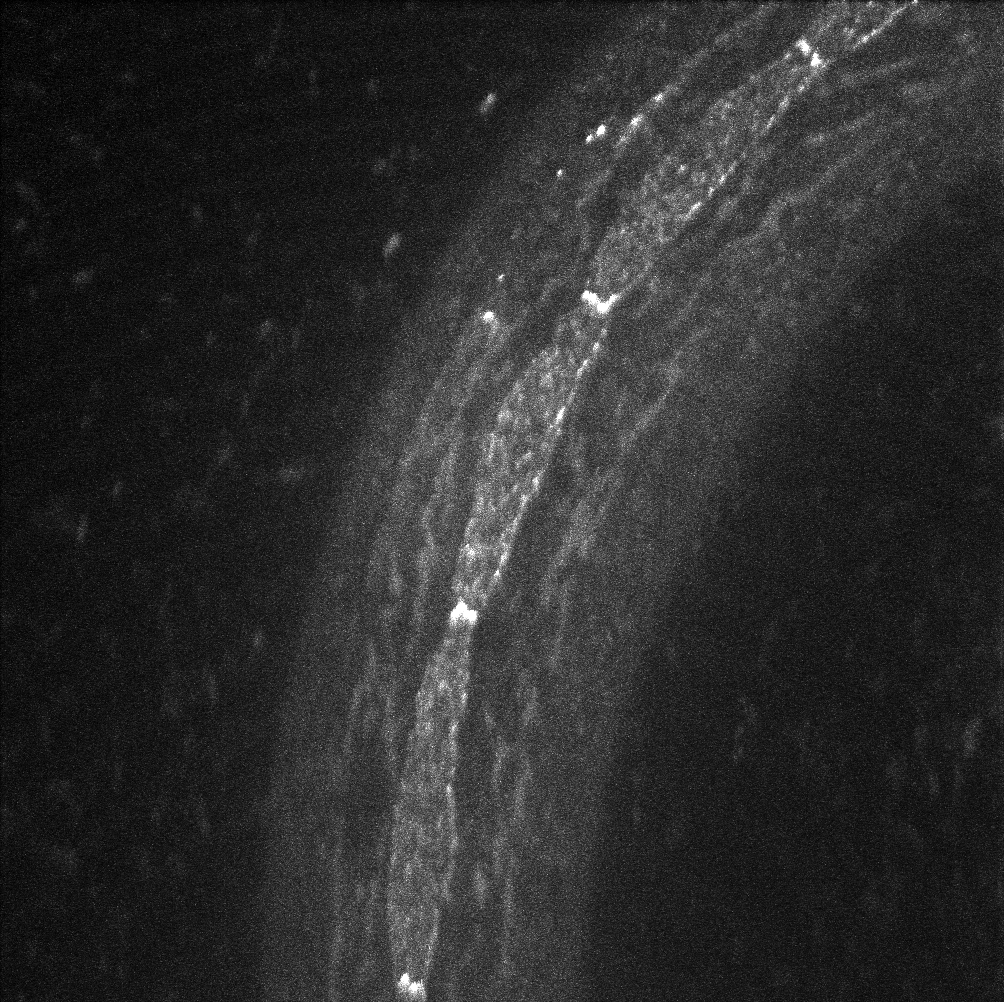

Supplement: Figure 6—source data 1. — This zip archive contains the microscopy images shown in panels A, C, E, G, and I, and the data graphed in panels B, D, F, H, and J. The images are in TIFF and Adobe Photoshop format. The TIFF file is the unadjusted grayscale maximum intensity projection image generated in ImageJ from an image stack. The Photoshop file contains the original image with the adjustment layers used to arrive at the final image displayed in the main figure. The raw graph data are in Microsoft Excel format, and the summary data and actual graph in Graphpad Prism format. [file elife-62067-fig6-data1.zip › Fig 6/A/MAX_200311_par6aid_noca1_C_2-4-to-7.tif]

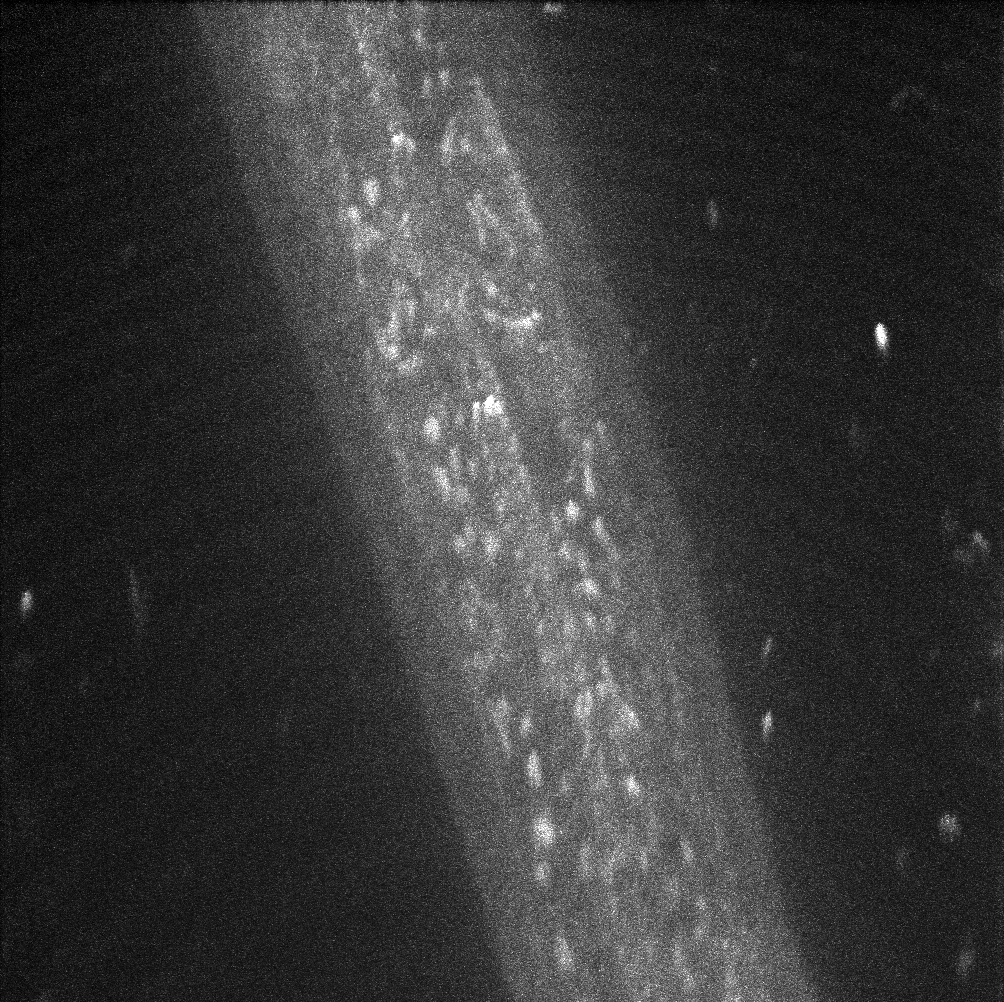

Supplement: Figure 6—source data 1. — This zip archive contains the microscopy images shown in panels A, C, E, G, and I, and the data graphed in panels B, D, F, H, and J. The images are in TIFF and Adobe Photoshop format. The TIFF file is the unadjusted grayscale maximum intensity projection image generated in ImageJ from an image stack. The Photoshop file contains the original image with the adjustment layers used to arrive at the final image displayed in the main figure. The raw graph data are in Microsoft Excel format, and the summary data and actual graph in Graphpad Prism format. [file elife-62067-fig6-data1.zip › Fig 6/A/MAX_200311_pra6aid_noca1_pwrt2_A_9-7-to-10.tif]

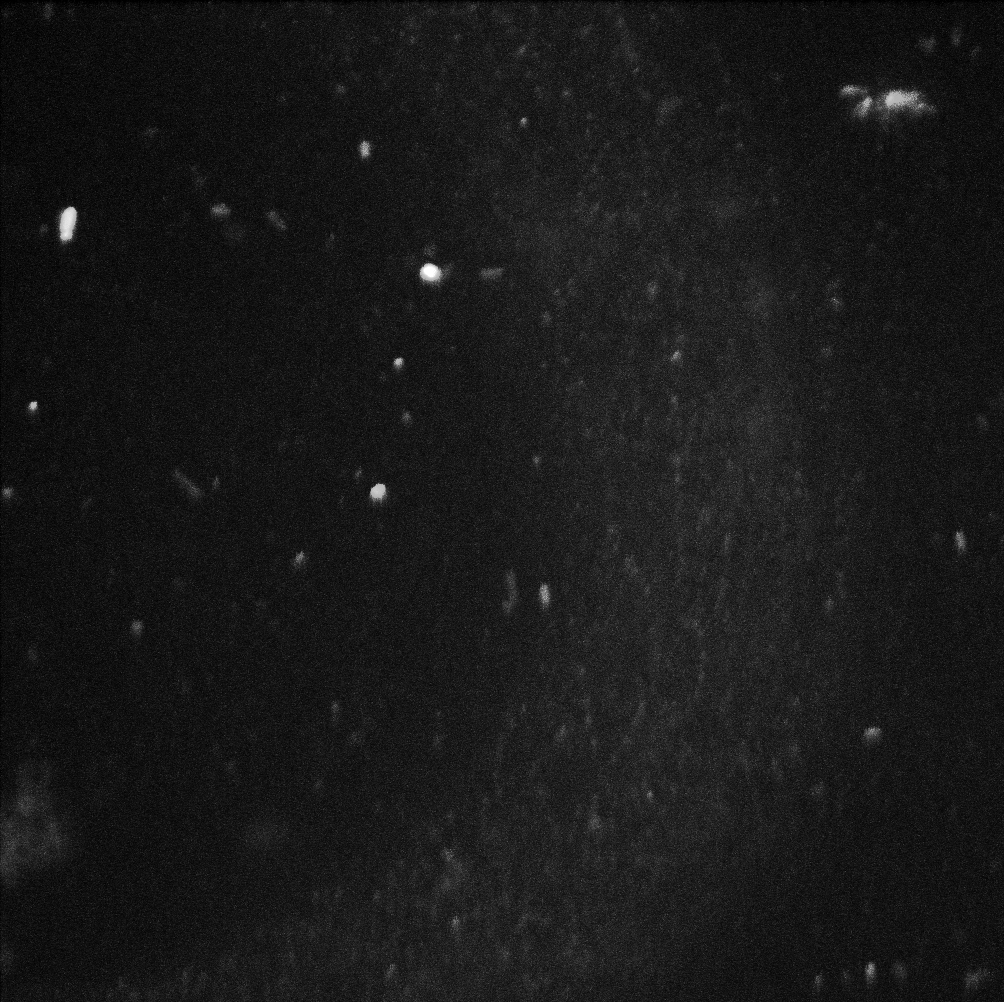

Supplement: Figure 6—source data 1. — This zip archive contains the microscopy images shown in panels A, C, E, G, and I, and the data graphed in panels B, D, F, H, and J. The images are in TIFF and Adobe Photoshop format. The TIFF file is the unadjusted grayscale maximum intensity projection image generated in ImageJ from an image stack. The Photoshop file contains the original image with the adjustment layers used to arrive at the final image displayed in the main figure. The raw graph data are in Microsoft Excel format, and the summary data and actual graph in Graphpad Prism format. [file elife-62067-fig6-data1.zip › Fig 6/C/MAX_200311_par6aid_gip1_A_2_w2SpinningDisc - Red-3-to-6.tif]

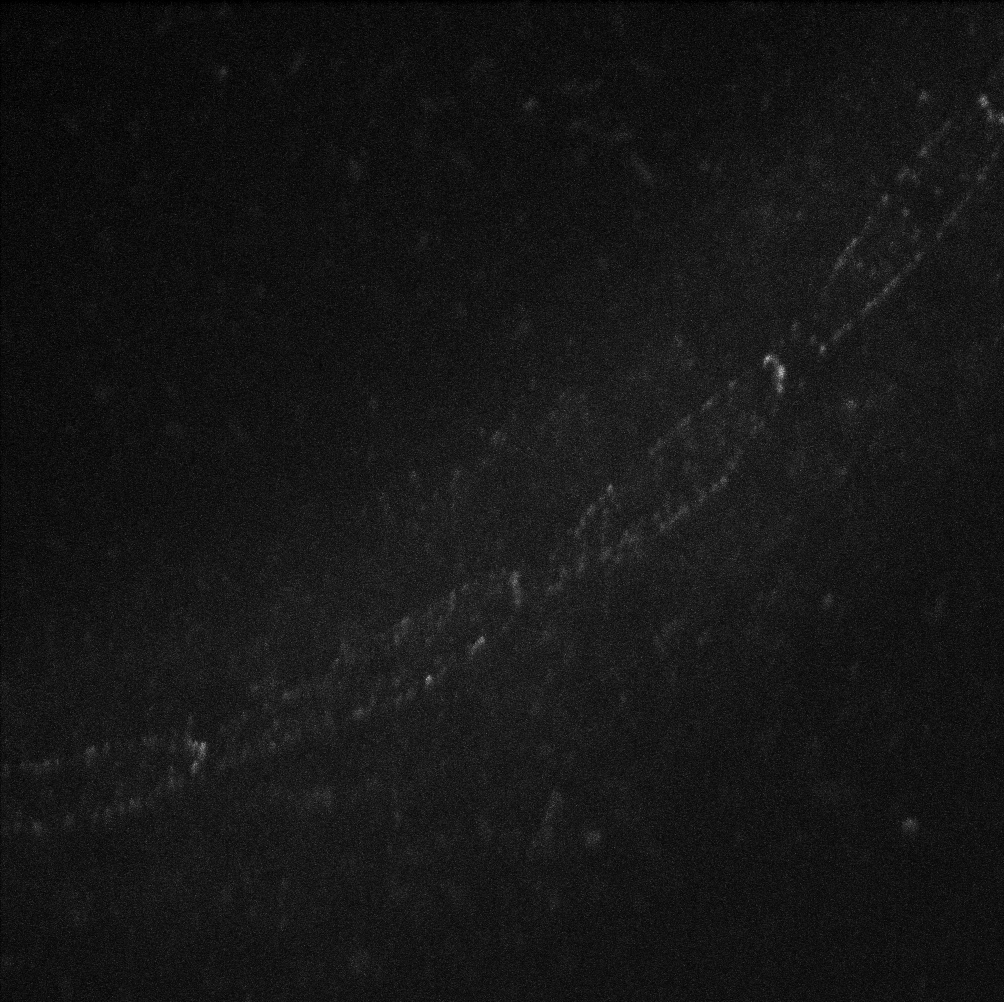

Supplement: Figure 6—source data 1. — This zip archive contains the microscopy images shown in panels A, C, E, G, and I, and the data graphed in panels B, D, F, H, and J. The images are in TIFF and Adobe Photoshop format. The TIFF file is the unadjusted grayscale maximum intensity projection image generated in ImageJ from an image stack. The Photoshop file contains the original image with the adjustment layers used to arrive at the final image displayed in the main figure. The raw graph data are in Microsoft Excel format, and the summary data and actual graph in Graphpad Prism format. [file elife-62067-fig6-data1.zip › Fig 6/C/MAX_200311_par6aid_gip1_C_5_w2SpinningDisc - Red-3-to-6.tif]

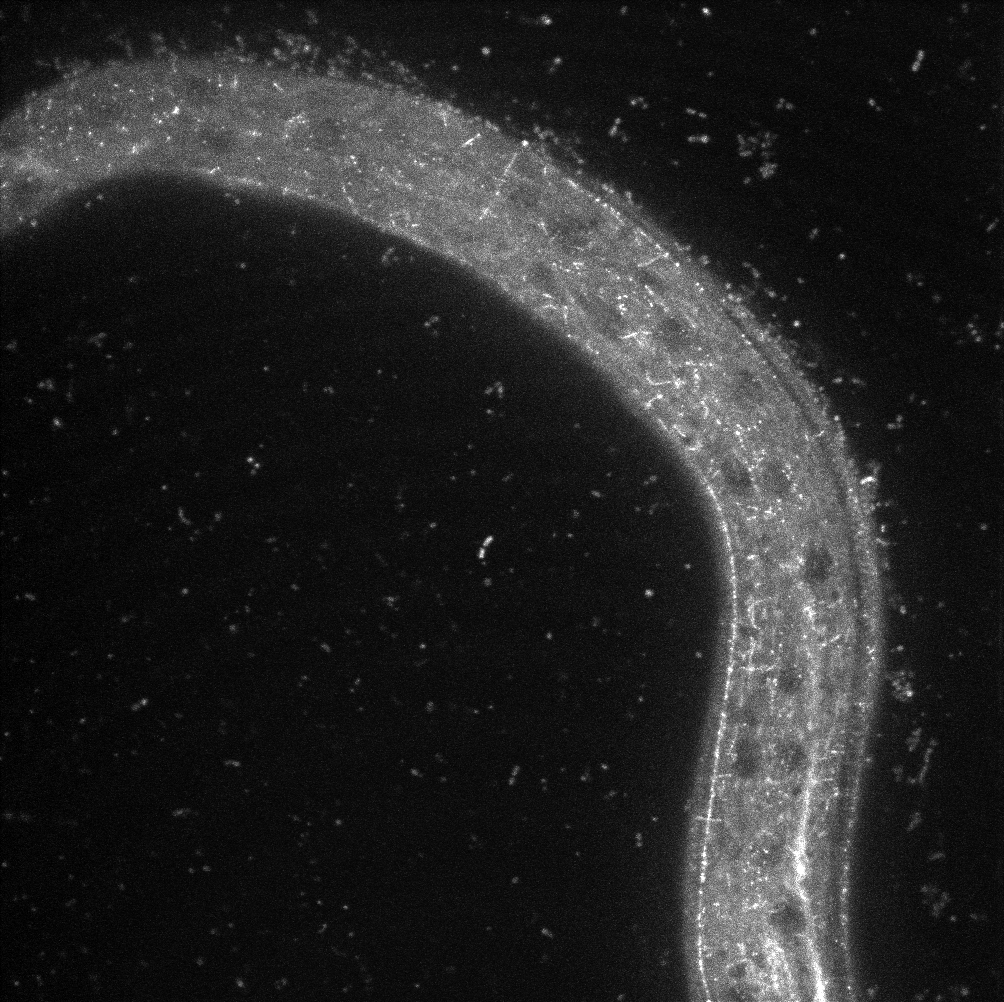

Supplement: Figure 6—source data 1. — This zip archive contains the microscopy images shown in panels A, C, E, G, and I, and the data graphed in panels B, D, F, H, and J. The images are in TIFF and Adobe Photoshop format. The TIFF file is the unadjusted grayscale maximum intensity projection image generated in ImageJ from an image stack. The Photoshop file contains the original image with the adjustment layers used to arrive at the final image displayed in the main figure. The raw graph data are in Microsoft Excel format, and the summary data and actual graph in Graphpad Prism format. [file elife-62067-fig6-data1.zip › Fig 6/E/MAX_190905_P6AID_PTRN_AUXIN_5-6-to-8.tif]

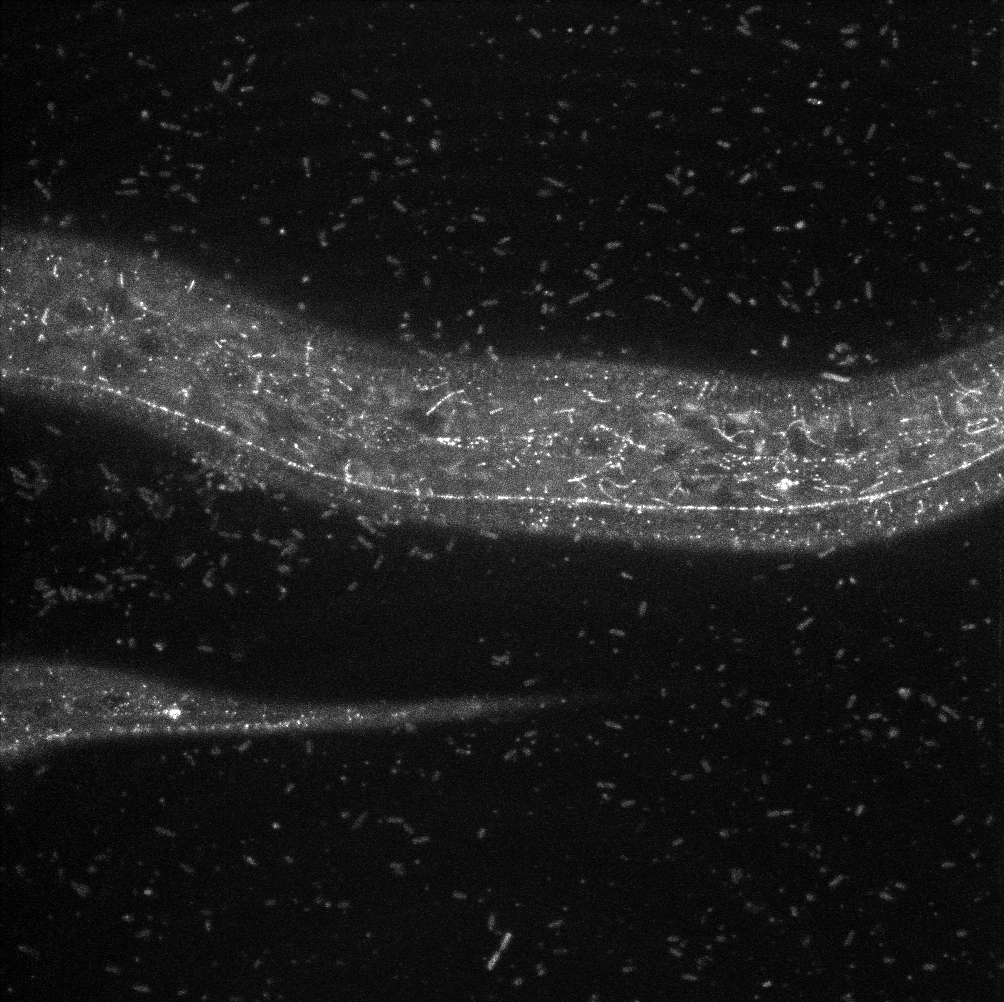

Supplement: Figure 6—source data 1. — This zip archive contains the microscopy images shown in panels A, C, E, G, and I, and the data graphed in panels B, D, F, H, and J. The images are in TIFF and Adobe Photoshop format. The TIFF file is the unadjusted grayscale maximum intensity projection image generated in ImageJ from an image stack. The Photoshop file contains the original image with the adjustment layers used to arrive at the final image displayed in the main figure. The raw graph data are in Microsoft Excel format, and the summary data and actual graph in Graphpad Prism format. [file elife-62067-fig6-data1.zip › Fig 6/E/MAX_190905_wow4_NGM_4-4-to-6.tif]

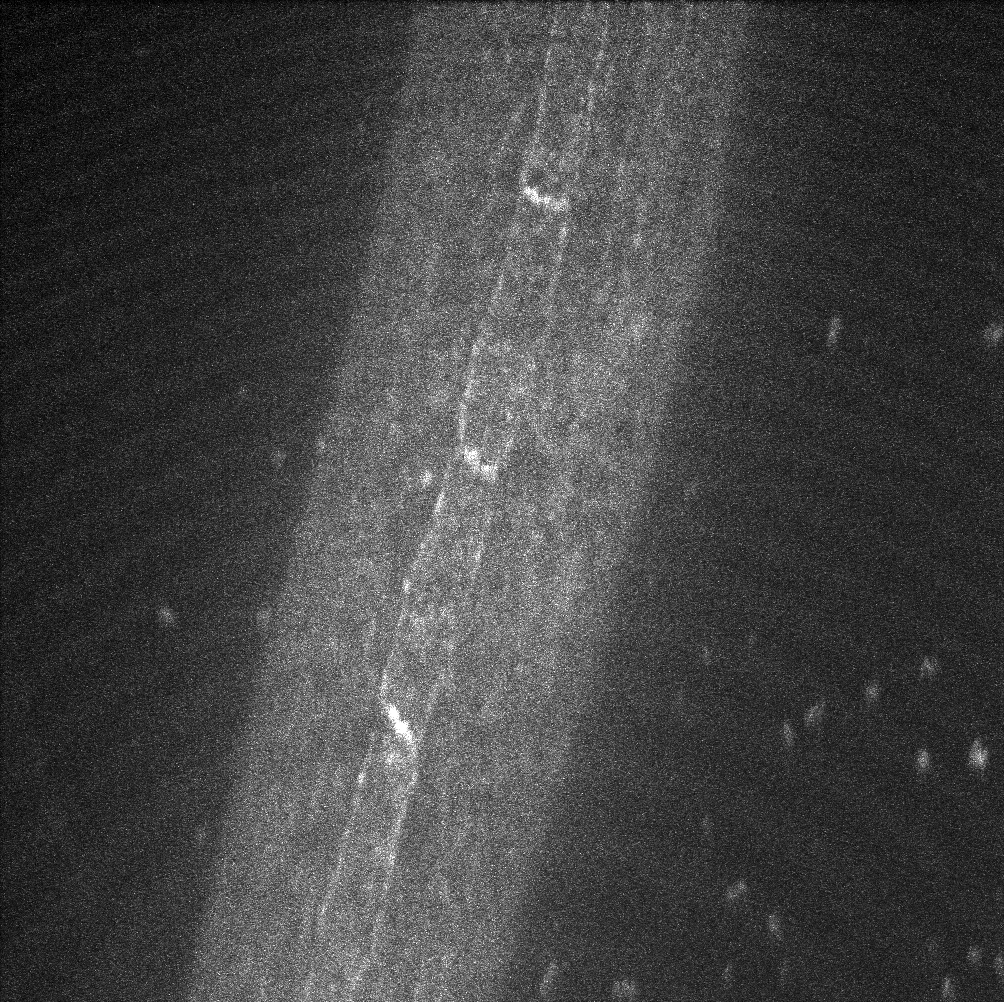

Supplement: Figure 6—source data 1. — This zip archive contains the microscopy images shown in panels A, C, E, G, and I, and the data graphed in panels B, D, F, H, and J. The images are in TIFF and Adobe Photoshop format. The TIFF file is the unadjusted grayscale maximum intensity projection image generated in ImageJ from an image stack. The Photoshop file contains the original image with the adjustment layers used to arrive at the final image displayed in the main figure. The raw graph data are in Microsoft Excel format, and the summary data and actual graph in Graphpad Prism format. [file elife-62067-fig6-data1.zip › Fig 6/G/MAX_201105_noca1degfp_C_5-5-to-7.tif]

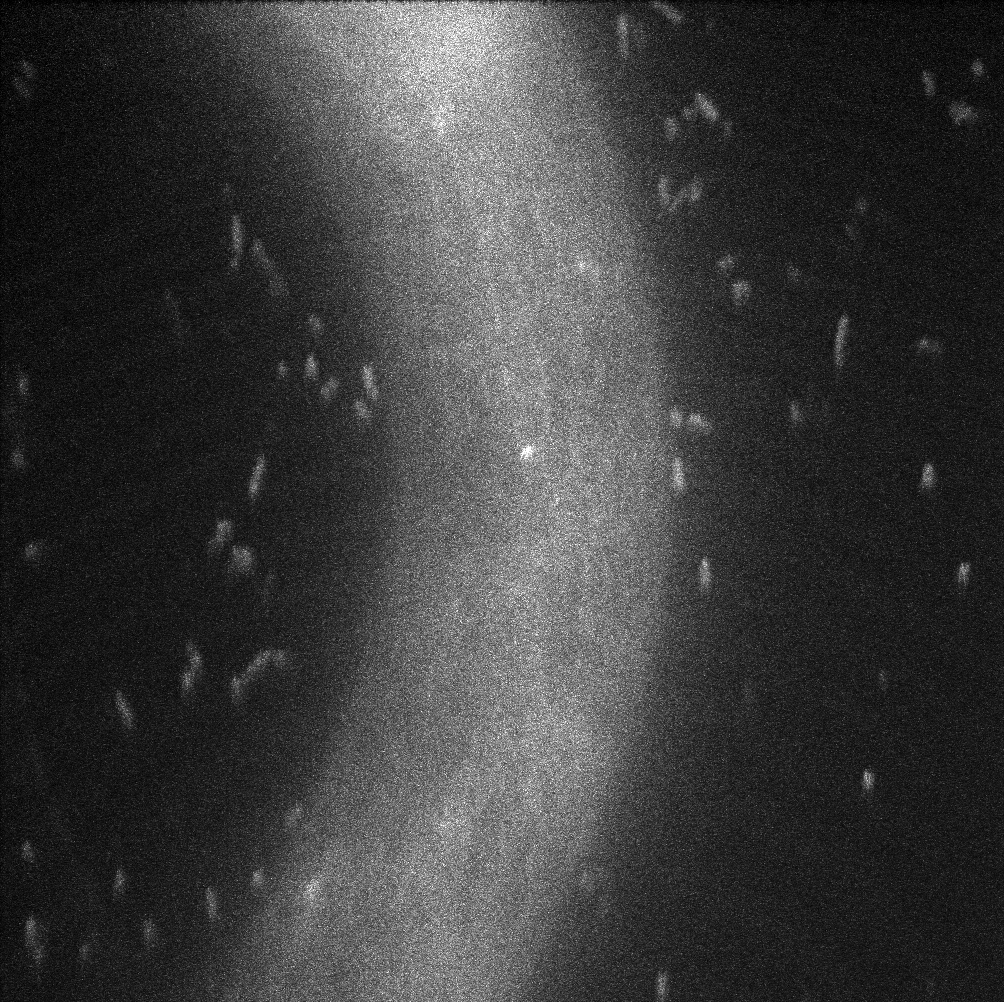

Supplement: Figure 6—source data 1. — This zip archive contains the microscopy images shown in panels A, C, E, G, and I, and the data graphed in panels B, D, F, H, and J. The images are in TIFF and Adobe Photoshop format. The TIFF file is the unadjusted grayscale maximum intensity projection image generated in ImageJ from an image stack. The Photoshop file contains the original image with the adjustment layers used to arrive at the final image displayed in the main figure. The raw graph data are in Microsoft Excel format, and the summary data and actual graph in Graphpad Prism format. [file elife-62067-fig6-data1.zip › Fig 6/G/MAX_201105_pkc3aid_noca1degfp_A_8-6-to-8.tif]

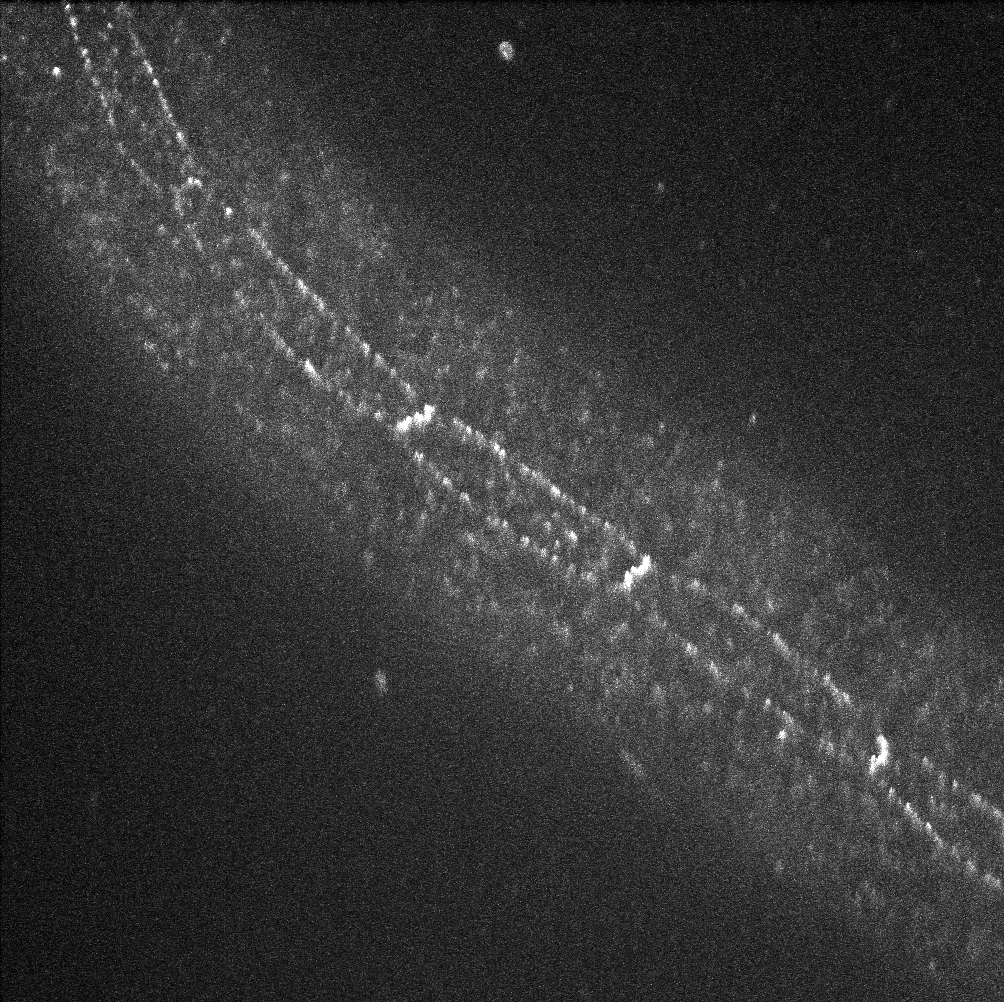

Supplement: Figure 6—source data 1. — This zip archive contains the microscopy images shown in panels A, C, E, G, and I, and the data graphed in panels B, D, F, H, and J. The images are in TIFF and Adobe Photoshop format. The TIFF file is the unadjusted grayscale maximum intensity projection image generated in ImageJ from an image stack. The Photoshop file contains the original image with the adjustment layers used to arrive at the final image displayed in the main figure. The raw graph data are in Microsoft Excel format, and the summary data and actual graph in Graphpad Prism format. [file elife-62067-fig6-data1.zip › Fig 6/I/MAX_200526_gip1_C_13_3_to_6.tif]

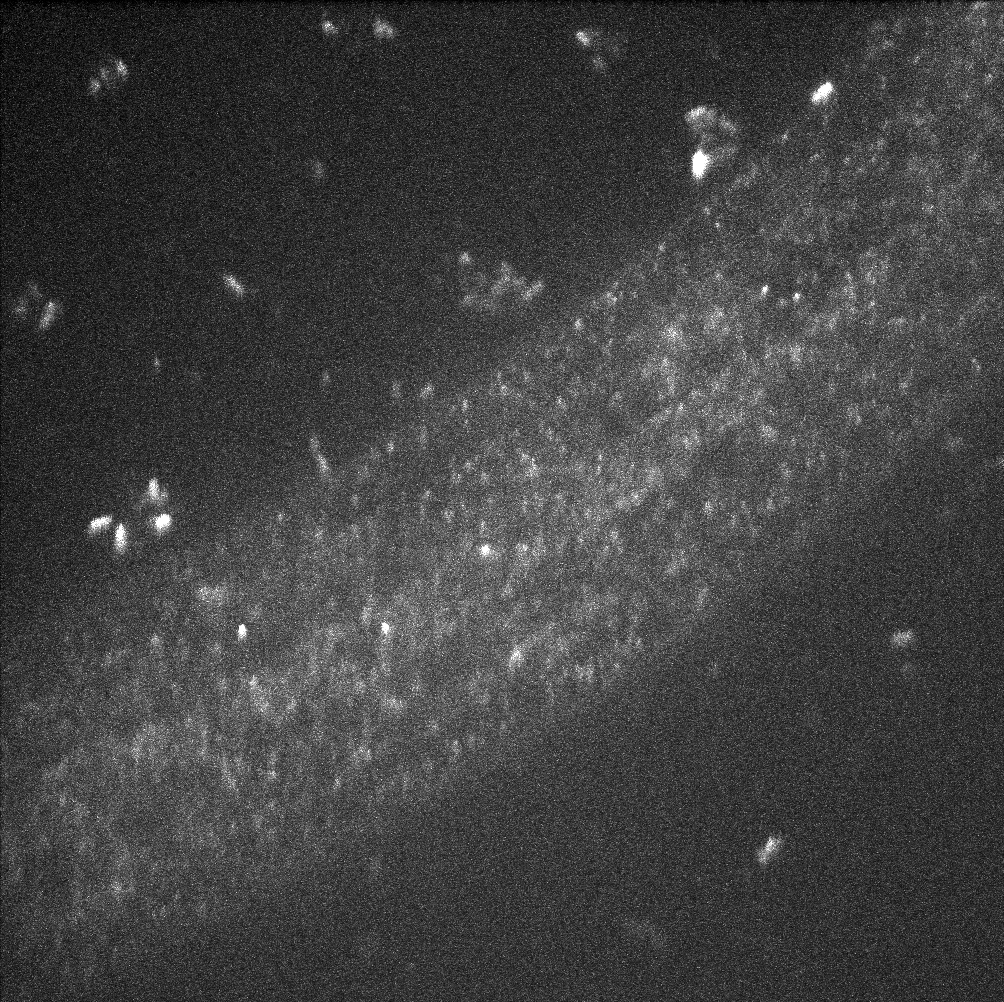

Supplement: Figure 6—source data 1. — This zip archive contains the microscopy images shown in panels A, C, E, G, and I, and the data graphed in panels B, D, F, H, and J. The images are in TIFF and Adobe Photoshop format. The TIFF file is the unadjusted grayscale maximum intensity projection image generated in ImageJ from an image stack. The Photoshop file contains the original image with the adjustment layers used to arrive at the final image displayed in the main figure. The raw graph data are in Microsoft Excel format, and the summary data and actual graph in Graphpad Prism format. [file elife-62067-fig6-data1.zip › Fig 6/I/MAX_200526_nocako_gip1_3_5_to_8.tif]

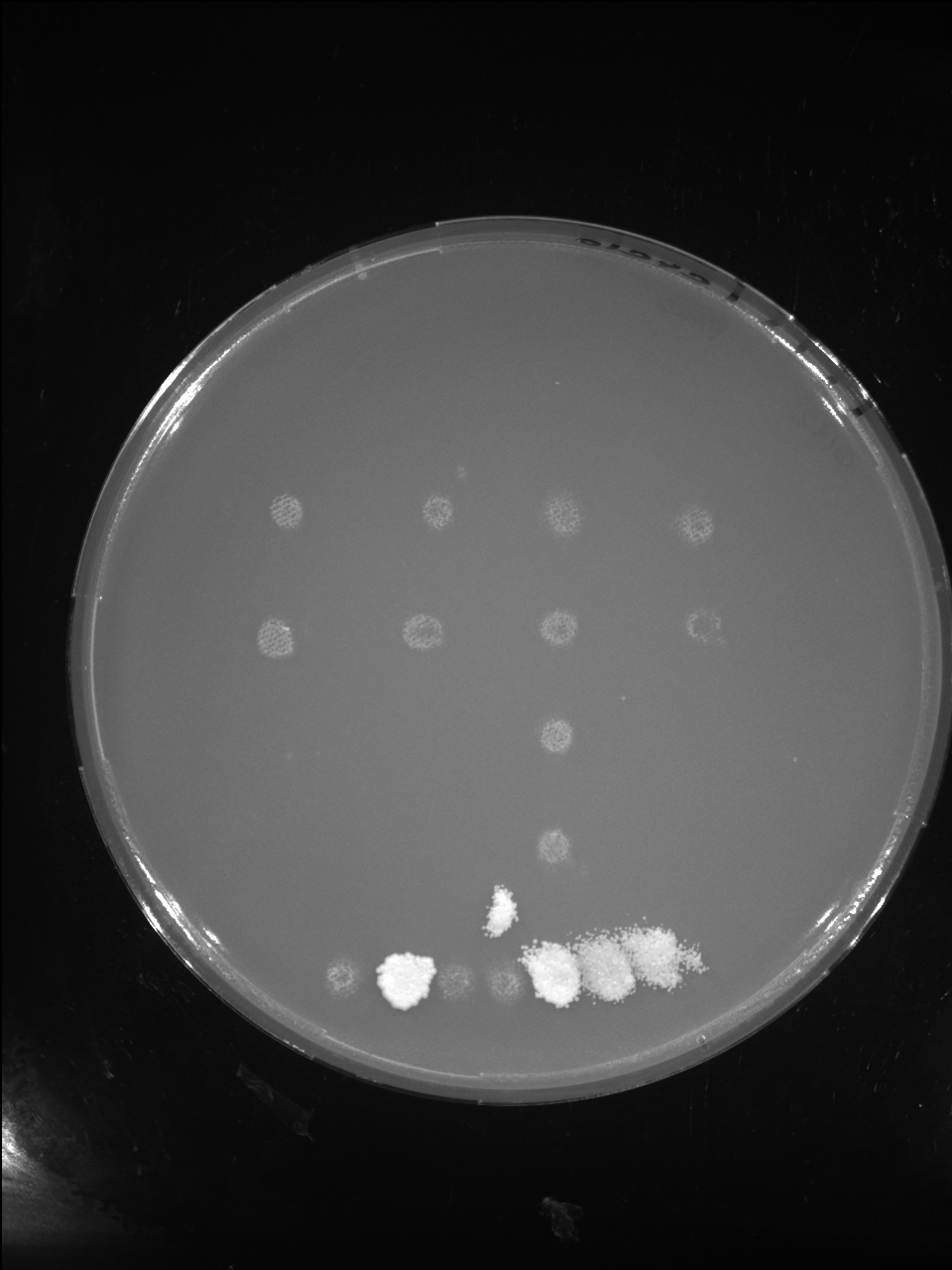

Supplement: Figure 6—figure supplement 1—source data 1. — This zip archive contains the uncropped TIFF images of the yeast growth on the three types of selective plates. [file elife-62067-fig6-figsupp1-data1.zip › Fig 6S1/Y2H mating +cycloheximide.tif]

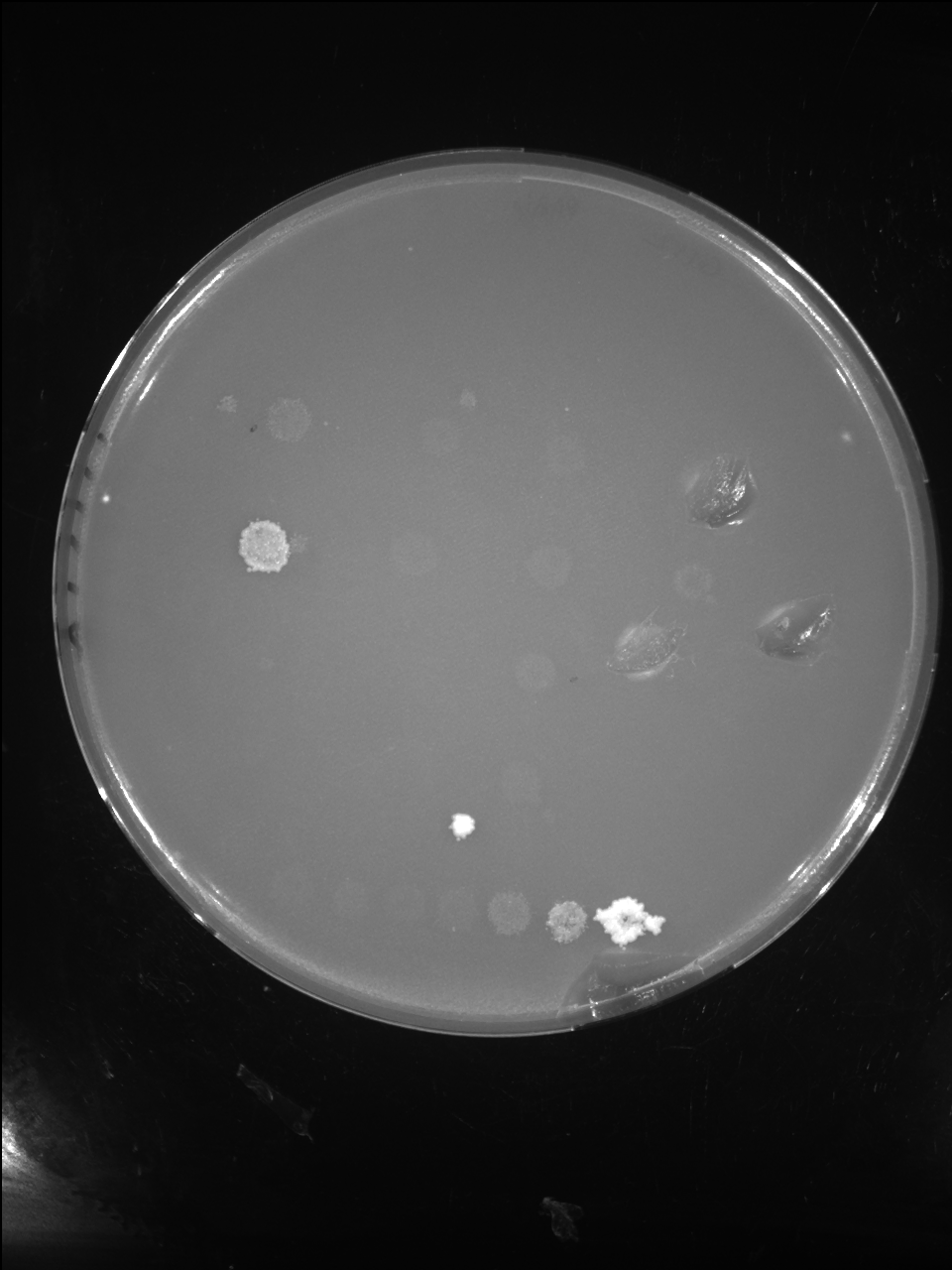

Supplement: Figure 6—figure supplement 1—source data 1. — This zip archive contains the uncropped TIFF images of the yeast growth on the three types of selective plates. [file elife-62067-fig6-figsupp1-data1.zip › Fig 6S1/Y2H mating -Ade.tif]

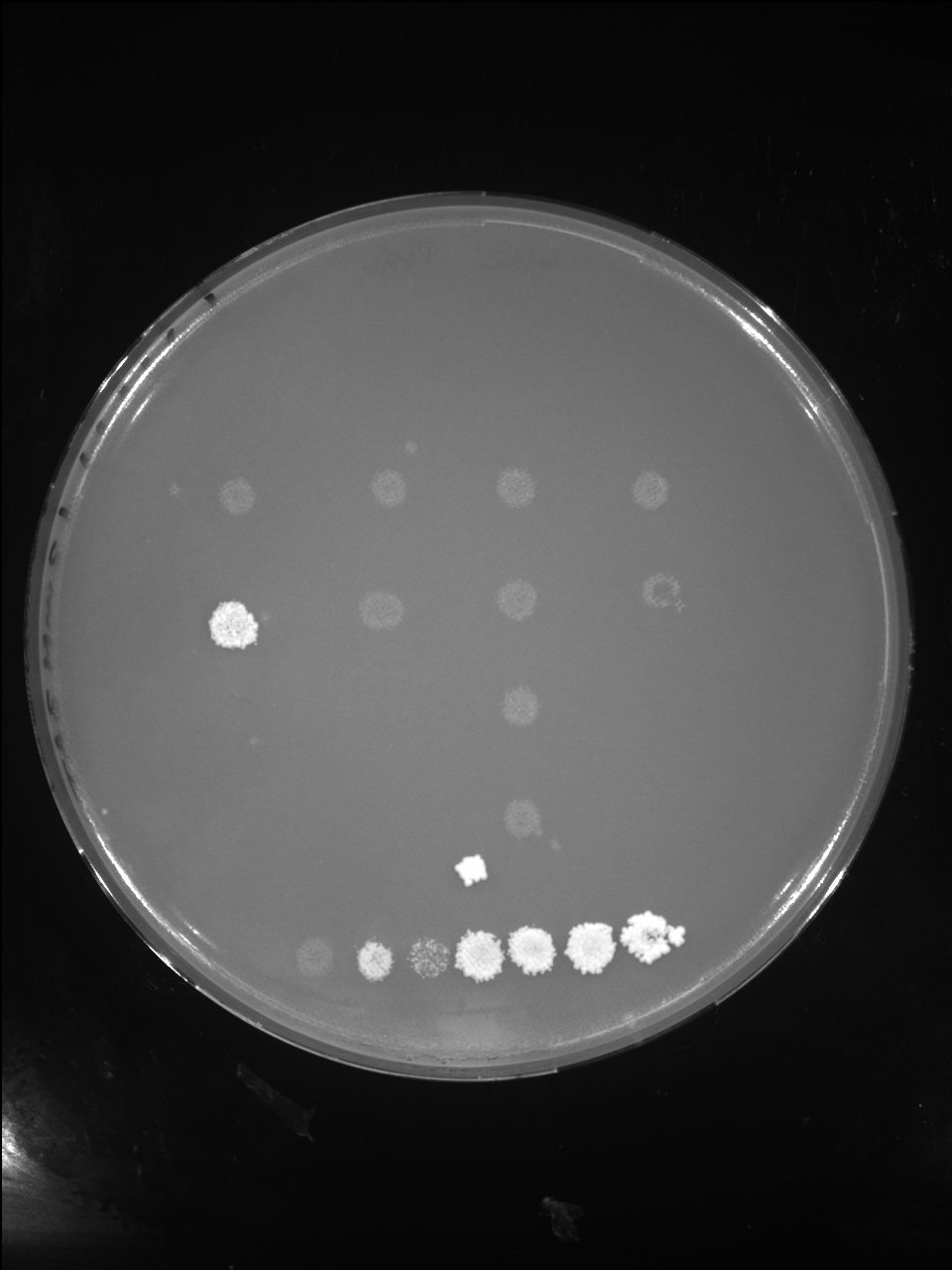

Supplement: Figure 6—figure supplement 1—source data 1. — This zip archive contains the uncropped TIFF images of the yeast growth on the three types of selective plates. [file elife-62067-fig6-figsupp1-data1.zip › Fig 6S1/Y2H mating 2mM 3AT.tif]
